# A Stepped Health Services Intervention to Improve Care for Mental and Neurological Diseases: Protocol for a Prospective Cohort Trial

**DOI:** 10.2196/37569

**Published:** 2023-01-17

**Authors:** Johannes Pollmanns, Karlheinz Großgarten, Julia K Wolff, Hans-Dieter Nolting, Clarissa Graf, Frank Bergmann, Gereon Nelles

**Affiliations:** 1 Association of Statutory Health Insurance Physicians in North Rhine Düsseldorf Germany; 2 IGES Institute GmbH Berlin Germany; 3 Central Research Institute of Ambulatory Health Care in Germany Berlin Germany; 4 Neuromed-Campus Cologne Germany

**Keywords:** mental health, neurology, quality of care, organization of health services, primary care, mental disorder, intervention, neurological disease, healthcare system, accessibility, quality of care, therapy

## Abstract

**Background:**

Mental and neurological disorders cause a large proportion of morbidity burden and require adequate health care structures. However, deficits in the German health care system like long waiting times for access to specialized care and a lack of coordination between health care providers lead to suboptimal quality of care and elevated health care costs.

**Objective:**

To overcome these deficits, we implement and evaluate a unique stepped and coordinated model of care (the Neurologisch-psychiatrische und psychotherapeutische Versorgung [NPPV] program) for patients with mental and neurological diseases.

**Methods:**

Patients included in the program receive an appropriate treatment according to medical needs in a multiprofessional network of ambulatory health care providers. The therapy is coordinated by a managing physician and complemented by additional therapy modules, such as group therapy, internet-based cognitive behavioral therapy, and a case management. Statutory health insurance (SHI) routine data and data from a longitudinal patient survey will be used to compare the program with regular care and evaluate SHI expenditures and patient-related outcomes. A health care provider survey will evaluate the quality of structure and processes and provider satisfaction. Finally, an analysis of ambulatory claims data and drug prescription data will be used to evaluate if health care providers follow a needs-led approach in therapy. Ethics approval for this trial was obtained from the ethics committee of the chamber of physicians in North Rhine (September 13, 2017, reference No. 2017287).

**Results:**

Patient enrollment of NPPV ended in September 2021. Data analysis has been completed in 2022. The results of this study will be disseminated through scientific publications, academic conferences, and a publicly available report to the German Federal Joint Committee, which is expected to be available in the first half of 2023.

**Conclusions:**

The NPPV program is the first intervention to implement a stepped model of care for both mental and neurological diseases in Germany. The analysis of several data sources and a large sample size (more than 14,000 patients) enable a comprehensive evaluation of the NPPV program.

**Trial Registration:**

German Clinical Trials Register DRKS00022754; https://tinyurl.com/3mx9pz5z.

**International Registered Report Identifier (IRRID):**

DERR1-10.2196/37569

## Introduction

Mental and neurological diseases continue to be a challenge for health systems worldwide. When measuring disability-adjusted life years, these diseases make up the largest share of all cause morbidity burden in the European Union [1]. Chronic mental and neurological diseases often lead to a loss of quality of life, disability, and even premature death and go along with significant direct and indirect costs of illness [2-4].

From 1990 to 2017, the 27 countries of the European Union and the United Kingdom showed an increase in prevalence of stroke (+25%), dementia (+62%), Parkinson disease (+72%), and multiple sclerosis (+41%), mainly due to demographic changes in an aging population [2]. According to the models by Brinks and Landwehr [5], the number of persons with dementia might increase 2-3 times by the year 2050. For Germany, Steffen et al [6] found an increase of 26% for depression between 2009 and 2017, especially for men and younger patients. Disease prevalence for depression with mild symptoms is on average higher in Germany as compared to other European countries [7].

The increasing burden of mental and neurological diseases will require robust health care structures in the near future to secure adequate treatment for these patients. However, several deficits in the German health care system prevent patients from getting the best possible care. First, timely access to specialized neuropsychiatric care is challenging for patients with mental and neurological diseases, mostly due to a lack of sufficient outpatient care facilities for neurology, psychiatry, and psychotherapy. Almost 3 quarters of patients with a mental disease in outpatient care are treated exclusively by their general practitioners and not by psychiatrists [8], and around 8.5% of patients fail to get an initial examination from a psychotherapist within 3 months [9]. Less than 55% of all patients with newly diagnosed multiple sclerosis receive specialized care by a neurologist within 6 weeks (dementia: less than 26%) [10]. Second, allocation of care occurs without adequate prioritization. According to Walendzik et al [11], half of the psychotherapists offer an appointment to the next inquiring patient without considering clinical needs. Third, treatments do not always follow medical guidelines. Even when excluding mild cases, about 50% of patients with depression do not receive the recommended standard of care at all or an adequate treatment with antidepressants or psychotherapy [12]. Fourth, the Advisory Council on the Assessment of Developments in the Health Care System in Germany criticized a lack of coordination and cooperation between health care sectors and professionals in the treatment of mental diseases and pointed out the need for a primary professional who coordinates the treatment of patients along clinical pathways [13].

To overcome these deficits and improve treatment of people with mental and neurological diseases, we developed a stepped and coordinated care program called “neurological-psychiatric and psychotherapeutic care” (Neurologisch-psychiatrische und psychotherapeutische Versorgung [NPPV]) for patients in the region of North Rhine, Germany. We hypothesize that our model of care will reduce direct and indirect costs of illness, improve quality of care and adherence with medical guidelines, enable a low-threshold access to care and increase health-related quality of life in patients as well as satisfaction with health care situation of patients and health care providers.

## Methods

### Intervention: The NPPV Program

The NPPV program is a stepped and coordinated model of care for mental and neurological diseases. The program aims at increasing efficiency of care, improve patients’ health outcomes, and reduce direct and indirect health costs. Each patient in the program receives an early evaluation of the patients’ needs for care followed by a timely and appropriate treatment in a multiprofessional network of outpatient health care providers. The network includes specialists from neurology and psychiatry as well as psychotherapists. To plan, coordinate, and ensure an individual treatment according to medical guidelines, every patient is assigned to a managing physician or therapist. These physicians/therapists are the primary contacts for the patients and guide the patient within the network and health care system in general. They also provide special consultation hours, cooperate with other health care professionals in the network and self-help organizations, and may choose from a wide range of additional therapy modules in the program. These modules include group therapy, internet-based cognitive behavioral therapy (ICBT), and an outpatient case management for patients carried out by the NPPV coordination office.

Group therapies are of particular importance for the treatment of mental and neurological diseases and are part of national medical guidelines for depression, posttraumatic stress disorder, or other severe mental diseases [14-16]. In the NPPV program, group therapies may be offered by all qualified health care providers in the network. Possible types of group therapy include (but are not limited to) psychoeducation, neuroeducation and training, strengthening of personal resources, and family counseling. These therapies are supported by the NPPV coordination office, for example, with regard to making appointments.

ICBT may be applied as an alternative or in addition to psychotherapeutic and psychiatric care. These therapies are low-threshold psychosocial interventions and are effective in reducing symptoms, especially for depression, anxiety disorders, panic disorders, and social phobia, and are highly accepted by patients [17-19]. In NPPV, we integrated the digital mental health program called Novego, which has been found to be effective in the reduction of anxiety and depressive symptoms in general and in patients with schizophrenia [20-22]. Novego offers 3 different courses with a focus on depression, burnout, or anxiety. The managing physician or psychotherapist can generate an access code to these courses for a patient. With these codes, patients are able to follow the courses on the internet in their own pace. The administrative tasks related to the courses are carried out by the NPPV coordination office.

Beside the central coordination office, health care providers in the NPPV network are supported by regional network managers. Tasks of the network managers are recruitment of new network members, implementation of trainings for the professionals, organization of regular quality circles, administrative support for professionals, and quality management. Additionally, physicians and psychotherapists are connected with a common web-based infrastructure (“IVPnet”) for patient administration, treatment management, and communication.

The program is funded by the Innovation Fund (“Innovationsfonds”), which was established to support innovative health care projects in Germany in 2015, and financed by the statutory health insurance (SHI) system.

### Study Design and Research Questions

The NPPV study is a prospective cohort study with a pragmatic design [23]. Beside the intervention group (participating patients), 2 control groups are drawn by matching techniques. Patients in the intervention group have immediate access to a stepped, coordinated, and comprehensive care program according to medical needs, whereas patients in the control groups receive regular care. In addition, health care providers are participating in a longitudinal survey.

We aim to answer the following research questions: first, does the intervention reduce SHI expenditures per patient? Second, are patients in the intervention group more satisfied with their treatment and do they show a higher quality of life than patients in the control group? Third, can the NPPV structures and processes be established as intended and do they lead to a higher satisfaction among health care providers? Fourth, can the NPPV program induce a more unmet-needs–based approach in the treatment of mental and neurological diseases (eg, do physicians and therapists focus on patient with a higher demand for care and do they follow medical guidelines more closely)?

### Study Population and Recruitment

We plan to recruit up to 14,000 patients for the intervention group in the region of North Rhine, Germany. The enrollment period begins in December 2017 and ends in June 2021. Patients eligible for participation in the NPPV program will be identified by their regular outpatient or inpatient health care providers or by their nursing home. They will be referred to a physician or therapist within the NPPV network, who does an entry examination to check eligibility. Inclusion criteria are as follows: age≥18 years, insurance in one of the participating health insurance companies (“AOK Rheinland/Hamburg,” “Continentale BKK,” and “BKK Deutsche Bank”), and 1 or more medical condition of 7 indication groups as outlined in Table 1. Participation in the NPPV program is voluntary. All patients need to give written informed consent to participate in the program and accompanied study.

**Table 1:** Medical inclusion and exclusion criteria for indication groups.

Indication group	ICD-10-GM-Codes^a^	Additional criteria
Affective disorders (including anxiety and depression, excluding mania and bipolar disorder)	F30-F39, F41.2	Inclusion when more than 3 weeks unable to work OR at least 2 hospitalizations in 2 years OR minimum score in Mini-ICF Rating for mental disorders
Psychosis (including mania and bipolar disorder)	F20- F31	Inclusion of all patients
Posttraumatic stress or adjustment disorder	F43	Inclusion of all patients
Dementia	F00, F01, G30	Inclusion of all patients
Multiple sclerosis	G35	Inclusion of all patients
Parkinson disease	G20-G22	Inclusion of all patients except vascular parkinsonism (G21.4)
Stroke	I63-I64	Inclusion of all patients with dysfunctions or disabilities, for example, spasticity or cognitive impairment

^a^ICD-10-GM: International Statistical Classification of Diseases and Related Health Problems.

### Study Outcomes and Data Sources

#### Overview

The primary study outcome is the overall SHI expenditures per patient in 12 months after inclusion. Secondary outcomes include SHI expenditures for psychiatric and neurological treatments, hospitalizations (frequency and length of stay), patient-related outcomes (ie, health-related quality of life, satisfaction with health care, and use of intervention modules), and provider-related outcomes (ie, satisfaction, structure and process quality, and needs-led approach).

Data to evaluate the outcomes stem from SHI routine data, outpatient claims data, drug prescription data, a patient survey, and a provider survey. All outcomes and the corresponding data sources are summarized in Table 2.

**Table 2:** Study outcomes and data sources.

Outcomes			Data source
**Primary outcome**			
	SHI^a^ expenditures per patient over 12 months	SHI routine data	
**Secondary outcomes**			
	1. SHI expenditures for psychiatric and neurological treatments per patient over 12 months	SHI routine data	
	2. Frequency of hospitalizations and length of stay over 12 months	SHI routine data	
	3. Frequency of hospitalizations with ICD^b^ codes from inclusion criteria and length of stay over 12 months	SHI routine data	
	4. Discontinuation of medical treatment over 12 months	SHI routine data	
	5. Number of days with incapacity for work over 12 months	SHI routine data	
	6. Health-related quality of life	Patient survey	
	7. Satisfaction with provided health care	Patient survey	
	8. Utilization of intervention modules	Patient survey	
	9. Health care provider satisfaction	Provider survey	
	10. Structure and process quality	Provider survey	
	11. Needs-led approach at health care provider level	Ambulatory claims data and drug prescription data	

^a^SHI: statutory health insurance.

^b^ICD: International Statistical Classification of Diseases and Related Health Problems.

#### SHI Routine Data

The participating health insurance companies (“AOK Rheinland/Hamburg,” “Continentale BKK,” and “BKK Deutsche Bank”) provide claims data on inpatient treatment, outpatient treatment, rehabilitation, drug prescriptions, and incapacity to work as well as basic personal information (eg, age, gender, and insurance periods) for participants as well as for 2 pools of control patients. Selection criteria for the first pool of control patients are ICD code of inclusion diagnosis (see Table 2; secured diagnosis of an outpatient health care provider that participates in the NPPV program), age≥18 years at time of diagnosis, and no participation in other special treatment contracts regarding the inclusion diagnoses (this is a pool to generate propensity scores for matching). The second pool consists of patients with the same selection criteria as the first pool, except that these patients should not be treated by a health care provider that is participating in the NPPV program (this is the pool to draw the control group via propensity score matching).

Data are provided at the end of the study and will include data from 4 quarters before inclusion date until March 2021 on each participant that started participation at least in March 2020 and control patients. Inclusion dates for potential control patients are determined by date of inclusion diagnosis. The permission to use the routine data of the participating insurance companies was granted by the State Ministry of Employment, Health and Social Affairs in North Rhine Westphalia.

#### Patient Survey

Patient-related outcomes are assessed in a longitudinal web-based patient survey. A control group is drawn by direct matching according to specific patient characteristics by the health insurance companies. Patients in the intervention group and the matched control group receive a link to a web-based survey from their respective health insurance company at 4 time points: immediately after inclusion in the intervention group (control group: after matching) and 3, 6, and 12 months after inclusion.

The questionnaire for the intervention and control group consists of several items on health care usage, satisfaction with health care (eg, waiting times and coordination of care), and health-related quality of life (see Multimedia Appendix 1). The latter is assessed using the WHOQOL BREF [24] in all indication groups complemented by indication-specific instruments on quality of life (Q-LES-Q 18 for affective disorders, psychosis and posttraumatic stress, or adjustment disorder [25]; DEMQoL for dementia [26]; MSIS for multiple sclerosis [27], PDQ-39 for Parkinson disease [28]; SA-SIP30 for stroke [29]). In addition, sociodemographic information (eg, age and gender) and data on general health status (eg, hospitalizations and comorbidities) are assessed.

#### Provider Survey

The quality of structure and processes and provider satisfaction is evaluated with a health care provider survey. All physicians, therapists, and case managers in the NPPV network are invited to participate in a survey carried out by the Central Research Institute of Ambulatory Health Care in Germany (Zi). The Zi developed a 2-part paper questionnaire (see Multimedia Appendix 2). Part A of the baseline survey contains 72 items and asks for an evaluation of structures and processes (eg, coordination and networking in NPPV and IT structures), provider satisfaction, and finally for an assessment of health care quality and workload. Part B of the baseline survey uses 10 questions to gather information about personal data such as age, gender, and details of health profession and health services. The anonymized questionnaire collects data every year to assess changes over time (4 times during the study period). In order to make comparisons, the structure of the surveys that followed the baseline survey remains the same. There were only additions that reflect current developments, for example, information on health care services during the COVID-19 pandemic.

#### Ambulatory Claims Data and Drug Prescription Data

An analysis of ambulatory claims data and drug prescription data from the Association of Statutory Health Insurance Physicians in North Rhine aims at evaluating if physicians and therapist focus on patients with high needs. As first source, the claims data contain, among others, outpatient diagnoses coded according to the German modification of the International Classification of Diseases (ICD-10-GM) from the first quarter of 2016 to the third quarter of 2020 for statutory healthinsured individuals in North Rhine. The second data source includes pharmacologic treatments information on the prescribed medication. The 2 data sources were linked together for the analysis. No data were available for residents with private health insurance.

### Sample Size Calculation

We calculated minimum sample size for the primary outcome (overall SHI expenditures per patient in 12 months) for which we expect a reduction by 10% in the intervention group as compared to the control group (assuming a coefficient of variation of 0.96). To identify this effect with a statistical power of 80% (α=.05), a minimum sample size of 1456 patients is required in the intervention group and the control group, respectively.

For analyses of secondary outcomes, an effect size of Cohen *d*=0.3 is expected for patient’s quality of life (mean difference of 5.38 points on the WHOQOL BREF scale between the intervention group and the control group with SD=17.92 [24]). The sample size required to identify this mean difference with a power of 80% (α=.05) is 175 patients per group.

### Data Analyses

#### Overview

Patient data (SHI routine data and patient survey) are analyzed for the total sample and for different subgroups based on indication groups. There are 2 main subgroups: mental diseases (affective disorders, psychosis and posttraumatic stress, or adjustment disorder) and neurological diseases (dementia, multiple sclerosis, Parkinson disease, and stroke). If sample size is sufficient, single indications groups will be analyzed separately. There will be no linkage between the SHI routine data and patient survey. For all analyses, significance level is set to 5%.

#### Analyses in SHI Routine Data

SHI routine data are used to evaluate the primary outcome and the secondary outcomes 1-5 (see Table 2). For all outcomes, NPPV participants are compared to a control group that is matched by propensity score matching [30]. Propensity scores are calculated using the data of the first pool of control patients and the participants in a multiple regression model estimating probability of participation in the NPPV program by personal (eg, age and gender) and health characteristics in the 12 months before inclusion date (eg, hospitalizations and frequency of out-patient treatments). The control group consists of matched persons of the second pool of control patients using the estimated propensity scores. To evaluate the outcomes, multiple regression models are estimated, controlling for relevant confounders. If necessary (high intraclass correlation coefficient), multilevel models are used to account for the hierarchical data structure (patients nested in outpatient health care providers that participate in NPPV).

#### Patient Survey Data

For the analyses of the secondary outcomes 6-8, the data of the longitudinal survey of participants and a control group are used. The control group is drawn by direct matching with the following matching variables: inclusion diagnosis from a health care provider that is not participating in the NPPV program, 10 years age groups, gender, no resident in a care facility, no legal guardian, and grouped number of inhabitants of place of residence. To evaluate the outcomes, descriptive statistics and regression analyses are conducted using longitudinal multilevel modeling techniques.

#### Provider Survey Data

The evaluation of the developed structures and processes of the project and the health care provider’s satisfaction (secondary outcomes 9 and 10 in Table 2) is based on provider survey data. These data were collected by interviewing all participants with a paper questionnaire. The aim is to evaluate if the processes and structures have been established as planned from the point of view of the participating providers. In this context, factors that promote or hinder the implementation of the project should be identified. All data will be collected completely anonymously, so that no conclusions can be drawn about the results of individual providers. Only an overall picture can be collected and compared. This analysis is part of the quality assurance and further development of the project.

#### Ambulatory Claims Data and Drug Prescription Data

To analyze secondary outcome 11, we use ambulatory claims data and drug prescription data. Ambulatory claims data are used to analyze the frequency of contact to ambulatory health providers. Therefore, the number of treatment cases will be compared to the need of a treatment. The question is if patients with more need of a treatment have higher numbers of treatment cases. A treatment case is defined as the sum of all medical services delivered by 1 physician during 1 quarter. Important diagnostic and therapeutic services for the NPPV patients will be defined and analyzed by checking if the patients receive these selected services and by computing the costs of ambulatory care. In addition, the analysis proves if the patients receive pharmacological care. It is analyzed whether they are under- or oversupplied. Within these analyses, patients who are part of the NPPV program are compared with patients who are not part of the NPPV program. The identification of patients who are part of the NPPV program is done by searching providers in the data who have billed NPPV services. When patients visit these providers, they are classified as an NPPV patient. The control group will be determined by using propensity score matching. The goal is to determine whether the treatment of the 2 patient groups differs and whether the program can improve the care of certain patient groups.

### Ethics Approval

Ethics approval for this trial was obtained from the ethics committee of the chamber of physicians in North Rhine (September 13, 2017, reference No 2017287).

## Results

Enrollment of physicians, therapists, and patients is completed. Data analysis was completed in the first half of 2022, and the results will be disseminated through scientific publications, academic conferences, and particularly a publicly available report to the German Federal Joint Committee. This report consists of a comprehensive summary over the study and a detailed description of all evaluation methods and corresponding results. The report is expected to be available after review by the German Federal Joint Committee in the first half of 2023 and can be found on the internet [31].

## Discussion

### Overview and Perspectives

This paper describes the protocol for a prospective cohort trial to evaluate the effects of the NPPV program. This program is unique and the first intervention to offer a low-threshold access for both mental and neurological diseases, implement a stepped model of care organized according to patients’ medical needs, and it builds up on a strong network of multiprofessional health care providers in Germany. We expect that NPPV will in particular reduce costs of illness, improve quality of care, and increase patient and provider satisfaction.

The Act to strengthen care in SHI (“GKV-Versorgungsstärkungsgesetz”) from 2015 included a structural reform of outpatient psychotherapy, which came into effect in 2017 [32]. While this reform improved the access to outpatient psychotherapeutic care [9], several evidence-practice gaps in the treatment of mental and neurological diseases prevail. We expect the consequences of the COVID-19 pandemic to further increase the medical and socioeconomic burden of both mental and neurological diseases and enhance the pressure on health care systems [33]. The NPPV program is designed to face these challenges and improve access to and quality of care. While there are several projects funded by Innovation Fund, which focus on mental or neurological diseases, most of them focus on selected settings, specific diseases, health care sectors, or treatment methods [13]. Section 92 of Book V of the German Code of Social Law (§92 SGB V) specifies that regulations on a multiprofessional, coordinated, and structured care for patients with severe mental diseases are to be established in order to advance the German health care system. Several elements of the NPPV program are part of this advancement.

### Strengths and Limitations

Our study has some strengths. We include 14,000 patients over 7 indication groups and analyze several different data sources (both primary and routine data). We also consider perspectives of patients, providers, and SHI in our analysis. Taken together, this enables a comprehensive evaluation of the NPPV program for patients with a wide range of mental and neurological diseases. However, our study also has some limitations. First, although we use matched control groups for the patient survey and analysis of routine data, our evaluation has a nonrandomized design. Second, we cannot completely rule out differences in disease severity between intervention and control groups. While an acutely higher disease severity increases the likelihood of being included in the program, this characteristic is not reflected in routine data used for our matching procedure. Third, we may face a selection bias in the patient survey as patients who are younger, healthier, and more educated are more likely to be willing to participate in our study. Finally, long-term effects (>24 months) on costs and patient outcomes cannot be evaluated in our analyses.

## Appendix

Multimedia Appendix 1 Questionnaires patient survey.

Multimedia Appendix 2 Questionnaires provider survey.

Multimedia Appendix 3 Confirmation government funding and assessment corresponding to a peer-review.

## Abbreviations

GBA: German Federal Joint Committee
ICBT: internet-based cognitive behavioral therapy
ICD: International Statistical Classification of Diseases and Related Health Problems
NPPV: Neurologisch-psychiatrische und psychotherapeutische Versorgung
SHI: statutory health insurance

## Data Availability

The data sets generated or analyzed during the current study are available from the corresponding author on reasonable request.
