# Supplementary material for: A Stepped Health Services Intervention to Improve Care for Mental and Neurological Diseases: Protocol for a Prospective Cohort Trial
Source: JMIR Res Protoc. 2023 Jan 17;12:e37569. doi: 10.2196/37569 (PMC9890347; doi:10.2196/37569)
Supplement: Multimedia Appendix 1 [file resprot_v12i1e37569_app1.pdf]

Hilfsvariablen zur Programmierung

Indikation = 1-7
Kodierung: 1 = Depression, 2 = Schizophrenie, schizotype oder wahnhafte Störungen, bipolare Störungen, 3 = Demenz; 4 = komplexe Traumafolgestörungen, 5 = Multiple Sklerose, 6 = Parkinson, 7 = Schlaganfall

Gruppe = 1;2
Kodierung: 1 = IG; 2 = KG

Einschub in Text beruhend auf Ausfülldatum:
[Monate] – die letzten 3 vollen Kalendermonate (z. B. „Januar, Februar und März"; wenn Ausfülldatum der 16.4.)
[Monat bis Monat] die letzten 6 vollen Kalendermonate (z. B. „Januar bis Juni"; wenn Ausfülldatum 13.7.)

Missings zulassen und kodieren: NULL
Kodiert im Pseudonym wird Gruppe und Indikation: Liste mit Pseudonymen + Indikation + Gruppe + Kennwort / Token
Speichern der Eingaben bei Abbruch: ja
Möglichkeit der Unterbrechung der Befragung: ja

Startseite

PROG: Gruppe = 1 (www.nppv-iges.de)

Herzlich Willkommen

Schön, dass Sie an der Befragung zur Qualität Ihrer Behandlung teilnehmen möchten. Sie helfen uns damit die Versorgung von Betroffenen zu verbessern.
Die Befragung findet im Rahmen der Versorgung Ihrer Erkrankung im Projekt zur Verbesserung der neurologisch-psychiatrischen und psychotherapeutischen Versorgung statt. Insgesamt werden Sie im Verlauf eines Jahres für 4 Befragungen kontaktiert.

Informationen zum Datenschutz

Ihre Daten werden streng nach den gesetzlichen Datenschutzbestimmungen verarbeitet. Es wird sichergestellt, dass die Daten der Befragung nicht mit Ihrer Person in Verbindung gebracht werden können. Zur Verknüpfung der vier Befragungszeitpunkte wird Ihnen ein Pseudonym zugewiesen. Nach Ende der letzten Befragung werden die Daten anonymisiert, so dass kein Personenbezug mehr herstellbar ist. Die Daten werden nur anonymisiert und in aggregierter Form veröffentlicht. Es finden keine Auswertungen für einzelne Personen statt. Nach Ablauf des Projektes werden die Daten unter Berücksichtigung der gesetzlichen Bestimmungen gelöscht.

Freiwilligkeit der Teilnahme

Ihre Teilnahme an der Befragung ist freiwillig und kann jederzeit ohne Angabe von Gründen beendet werden. Auch können Sie bis zum Zeitpunkt der Anonymisierung nach der letzten Befragung verlangen, dass alle von Ihnen erhobenen Daten gelöscht werden.
Weitere Informationen zur Teilnahme und zum Datenschutz finden Sie auch im Anschreiben Ihrer Krankenkasse.

Wenn Sie an der Befragung teilnehmen möchten und der beschriebenen Verarbeitung Ihrer Daten zustimmen, geben Sie bitte hier Ihr Passwort ein, das Sie von Ihrer Krankenkasse im Anschreiben erhalten haben:

PROG: Gruppe 2 (www.nppv-iges.de/KG)

Herzlich Willkommen

Schön, dass Sie an der Befragung zur Qualität Ihrer Behandlung teilnehmen möchten. Sie helfen uns damit die Versorgung von Betroffenen zu verbessern.
In dieser Befragung geht es um die Versorgung Ihrer seelischen und/oder neurologischen Erkrankung. Zurzeit werden Sie aufgrund dieser Erkrankung nach dem aktuellen Standard der Regelversorgung behandelt.

Um die Versorgung hierfür zu verbessern, fördert der Gesetzgeber neue Versorgungsprojekte im Rahmen des sogenannten *Innovationsfonds*. Eines dieser Projekte ist die Verbesserung der neurologischen-psychiatrischen und psychotherapeutischen Versorgung (NPPV).
Ziel von NPPV ist es, die Behandlung von psychischen und neurologischen Krankheiten zu verbessern. Dazu wird dem/r Patienten/in ein Bezugsarzt oder -therapeut bzw. eine Bezugsärztin oder -therapeutin an die Seite gestellt, der/die die verschiedenen Behandler/innen vernetzt und die Versorgung koordiniert.
Wir möchten herausfinden, ob NPPV die Versorgung spürbar verbessern kann und benötigen dazu Ihre Hilfe: Sie sind eingeladen an dieser Befragung teilzunehmen und durch Ihre Erfahrungen mit der bisherigen Regelversorgung einen wichtigen Beitrag zur Beurteilung der Versorgungsformen beizusteuern.
Die Ergebnisse dieser Befragungen können dazu führen, dass zukünftig alle gesetzlich Krankenversicherten von neuen Versorgungsformen profitieren.

Einverständniserklärung zur Datenverarbeitung
Für den Landesteil Nordrhein wird ein Projekt zur Verbesserung der neurologisch-psychiatrischen und psychotherapeutischen Versorgung (NPPV-Projekt) durchgeführt.
Ich nehme an dem NPPV-Projekt nicht teil. Ich bin aber durch das ausführliche Anschreiben meiner Krankenkasse eingehend darüber informiert worden, dass für alle gesetzlich krankenversicherten Patienten mit entsprechender Erkrankung ein Nutzen aus dem NPPV-Projekt nur festgestellt werden kann, wenn Daten von teilnehmenden Patienten mit Daten von nicht teilnehmenden Patienten (sog. Kontrollgruppe) verglichen werden.
Ich komme für eine Befragung in der sog. Kontrollgruppe in Betracht und stehe für diese zur Verfügung.
Ich habe darüber hinaus den Text der nachfolgenden Datenschutzerklärung gelesen und verstanden.
Datenschutzerklärung
Mir ist bekannt, dass personenbezogene Daten, d. h. Stammdaten (Name, Anschrift, Geburtsdatum etc.) sowie erforderliche Gesundheitsdaten (medizinische Behandlungsdaten) über mich bei meiner Krankenkasse erhoben, gespeichert und verarbeitet werden. Die Verwendung der Daten erfolgt nach gesetzlichen Bestimmungen. Ich erteile hiermit ausdrücklich die folgenden datenschutzrechtlichen Einwilligungserklärungen.
Ich erkläre mich damit einverstanden, dass meine Krankenkasse mir einen Fragebogen zur wissenschaftlichen Evaluation (Auswertung) zur Verfügung stellt, den ich online ausfülle und dessen Antworten pseudonymisiert (d. h. mit einer Kennung, aber ohne meinen Namen, Kontaktdaten oder sonstigen Angaben) bei der mit der wissenschaftlichen Evaluation beauftragten IGES Institut GmbH, Friedrichstraße 180, 10117 Berlin – vertretungsberechtigte Geschäftsführer: Prof. Dr. Bertram Häussler (Vorsitzender der Geschäftsführung), Dr. Martin Albrecht, Christoph Gipp, Hans-Dieter Nolting – gespeichert und verarbeitet werden. Die Befragung erfolgt ausschließlich bezogen auf eventuell bei mir vorliegende Krankheitsbilder, die auch Gegenstand des NPPV-Projekts sind; Rückschlüsse auf meine Person sind ausgeschlossen.

Ich bin darüber aufgeklärt worden, dass ich jederzeit meine Einwilligung zur Datenverarbeitung widerrufen kann und bis zum Zeitpunkt der Anonymisierung (nach Abschluss der letzten Befragung) verlangen kann, dass alle von mir erhobenen Daten gelöscht werden.

Für gesetzliche Vertreter/innen, Betreuer/innen bzw. Vorsorgebevollmächtigte:
Ich habe die obigen Ausführungen zur Einverständniserklärung zur Datenverarbeitung sowie die Datenschutzerklärung sorgfältig gelesen. Hiermit erkläre ich die Einwilligung des von mir betreuten bzw. vertretenen Patienten.

Wenn Sie an der Befragung teilnehmen möchten und der beschriebenen Verarbeitung Ihrer Daten zustimmen, geben Sie bitte hier Ihr Passwort ein, das Sie von Ihrer Krankenkasse im Anschreiben erhalten haben:

Übersicht über Ihre Befragungen

PROG: kein Filter

Insgesamt wird es vier Online-Befragungen geben, zu denen Sie jeweils von Ihrer Krankenkasse eingeladen werden. Die zweite Befragung findet ca. 3 Monate, die dritte ca. 6 Monate und die vierte ca. 12 Monate nach der ersten Befragung statt.
Bitte klicken Sie auf die entsprechende Befragung, um mit dem Ausfüllen des Fragebogens zu beginnen.

Befragung 1
Befragung 2
Befragung 3
Befragung 4

Einleitungstext

PROG: Gruppe = 1

	Lieber Teilnehmer, liebe Teilnehmerin,<br>Wir freuen uns sehr, dass Sie sich entschieden haben, an dem neuen Versorgungsmodell für Menschen mit psychischen und neurologischen Erkrankungen (NPPV) teilzunehmen. Um herauszufinden, wie gut Ihnen die neue Versorgungsform hilft und wie Sie diese bewerten, werden wir Ihnen viermal im Verlauf eines Jahres Fragen zu Ihrer Person, Ihrem Gesundheitszustand und Ihren Erfahrungen mit der neuen Versorgungsform stellen.<br><br>Das Ausfüllen wird etwa 35 Minuten in Anspruch nehmen. Sie können die Befragung jederzeit unterbrechen und zu einem späteren Zeitpunkt mit demselben Passwort fortsetzen. Sie können selbst entscheiden, ob Sie den Fragebogen alleine oder gemeinsam mit einer Bezugsperson ausfüllen. Die Bezugsperson kann die Beantwortung der Fragen auch vollständig übernehmen. Bitte geben Sie am Ende der Befragung an, wer den Fragebogen vorwiegend ausgefüllt hat.<br><br>Danke, dass Sie sich die Zeit nehmen die Fragen zu beantworten.<br><br>Bei Fragen zu der Befragung wenden Sie sich bitte an:<br>Tobias Woköck<br>Tel:<br>Email:

PROG: Gruppe = 2

	Lieber Teilnehmer, liebe Teilnehmerin,<br>Wir freuen uns sehr, dass Sie sich bereit erklären, diesen Fragebogen zu der Versorgung Ihrer Erkrankung auszufüllen. Sie helfen uns damit, die Versorgung ein Stück besser zu machen.<br>Um herauszufinden, wie gut Ihnen Ihre Versorgung hilft und wie Sie diese bewerten, werden wir Ihnen viermal im Verlauf eines Jahres Fragen zu Ihrer Person, Ihrem Gesundheitszustand und Ihren Erfahrungen mit der Versorgung Ihrer Erkrankung stellen.<br><br>Das Ausfüllen wird etwa 35 Minuten in Anspruch nehmen. Sie können die Befragung jederzeit unterbrechen und zu einem späteren Zeitpunkt mit demselben Passwort fortsetzen. Sie können selbst entscheiden, ob Sie den Fragebogen alleine oder gemeinsam mit einer Bezugsperson ausfüllen. Die Bezugsperson kann die Beantwortung der Fragen auch vollständig übernehmen. Bitte geben Sie am Ende der Befragung an, wer den Fragebogen vorwiegend ausgefüllt hat.

	Danke, dass Sie sich die Zeit nehmen die Fragen zu beantworten.<br><br>Bei Fragen zur Befragung wenden Sie sich bitte an:<br>Tobias Woköck<br>Tel:<br>Email:

PROG: Datum und Zeitstempel erfassen

1. Soziodemographie I

Seitenüberschrift: Angaben zur Person

PROG: kein Filter

Zum Beginn möchten wir Sie um einige kurze Angaben zu Ihrer Person bitten.

Falls Sie den Fragebogen stellvertretend für jemanden ausfüllen, beantworten Sie bitte alle Fragen aus Sicht der erkrankten Person.

001 Wie ist Ihr Geburtsjahr?

______ (numerisch, 4-stellig, min 1900)

002 Welches ist Ihr Geschlecht? ❑ männlich (1) ❑ weiblich (2)

003 Wie ist Ihr Familienstand?

❑ verheiratet, mit Ehepartner zusammenlebend (1)
❑ verheiratet, von Ehepartner dauerhaft getrennt lebend (2)
❑ in fester Partnerschaft (3)
❑ geschieden (4)
❑ verwitwet (5)
❑ ledig (6)

2. Gesundheitsbezogene Lebensqualität (generisch)

Seitenüberschrift: Gesundheit & Lebensqualität

WHOQOL-BREF (26 Items):
Quelle: Hogrefe
Lizenz: gekauft

PROG: kein Filter

Die folgenden Fragen beschäftigen sich mit Ihrer Lebensqualität, Ihrer Gesundheit und anderen Bereichen Ihres Lebens. Wir möchten möglichst genau erfahren, wie es Ihnen im Allgemeinen und mit spezifischen Erkrankungen geht.
Bitte denken Sie daran, falls Sie den Fragebogen stellvertretend für jemanden ausfüllen, alle Fragen aus Sicht der erkrankten Person zu beantworten.

Bitte lesen Sie jede Frage, überlegen Sie, wie Sie sich in den letzten zwei Wochen gefühlt haben und geben Sie die Antwort an, die am ehesten auf Sie zutrifft.

		sehr schlecht	schlecht	mittelmäßig	gut	sehr gut
101	Wie würden Sie Ihre Lebensqualität beurteilen?	1	2	3	4	5
		sehr unzufrieden	unzufrieden	weder zufrieden noch	zufrieden	sehr zufrieden

			unzufrieden		
**102**	Wie zufrieden sind Sie mit Ihrer Gesundheit?	1	2	3	4	5
	**In den folgenden Fragen geht es darum, wie stark Sie <u>während der letzten zwei Wochen</u> bestimmte Dinge erlebt haben.**					
		überhaupt nicht	ein wenig	mittelmäßig	ziemlich	äußerst
**103**	Wie stark werden Sie durch Schmerzen daran gehindert, notwendige Dinge zu tun?	1	2	3	4	5
**104**	Wie sehr sind Sie auf medizinische Behandlung angewiesen, um das tägliche Leben zu meistern?	1	2	3	4	5
**105**	Wie gut können Sie Ihr Leben genießen?	1	2	3	4	5
**106**	Betrachten Sie Ihr Leben als sinnvoll?	1	2	3	4	5
**107**	Wie gut können Sie sich konzentrieren?	1	2	3	4	5
**108**	Wie sicher fühlen Sie sich in Ihrem täglichen Leben?	1	2	3	4	5
**109**	Wie gesund sind die Umweltbedingungen in Ihrem Wohngebiet?	1	2	3	4	5
	**In den folgenden Fragen geht es darum, in welchem Umfang Sie <u>während der letzten zwei Wochen</u> bestimmte Dinge erlebt haben oder in der Lage waren, bestimmte Dinge zu tun.**					
		überhaupt nicht	eher nicht	halbwegs	überwiegend	völlig
**110**	Haben Sie genug Energie für das tägliche Leben?	1	2	3	4	5
**111**	Können Sie Ihr Aussehen akzeptieren?	1	2	3	4	5
**112**	Haben Sie genug Geld, um Ihre Bedürfnisse erfüllen zu können?	1	2	3	4	5
**113**	Haben Sie Zugang zu den Informationen, die Sie für das tägliche Leben brauchen?	1	2	3	4	5
**114**	Haben Sie ausreichend Möglichkeiten zu Freizeitaktivitäten?	1	2	3	4	5
		sehr schlecht	schlecht	mittelmäßig	gut	sehr gut

**115**	Wie gut können Sie sich fortbewegen?	1	2	3	4	5
	**In den folgenden Fragen geht es darum, wie zufrieden, glücklich oder gut Sie sich <u>während der letzten zwei Wochen</u> hinsichtlich verschiedener Aspekte Ihres Lebens gefühlt haben.**					
		sehr unzufrieden	unzufrieden	weder zufrieden noch unzufrieden	zufrieden	sehr zufrieden
**116**	Wie zufrieden sind Sie mit Ihrem Schlaf?	1	2	3	4	5
**117**	Wie zufrieden sind Sie mit Ihrer Fähigkeit, alltägliche Dinge erledigen zu können?	1	2	3	4	5
**118**	Wie zufrieden sind Sie mit Ihrer Arbeitsfähigkeit?	1	2	3	4	5
**119**	Wie zufrieden sind Sie mit sich selbst?	1	2	3	4	5
**120**	Wie zufrieden sind Sie mit Ihren persönlichen Beziehungen?	1	2	3	4	5
**121**	Wie zufrieden sind Sie mit Ihrem Sexualleben?	1	2	3	4	5
**122**	Wie zufrieden sind Sie mit der Unterstützung durch Ihre Freunde?	1	2	3	4	5
**123**	Wie zufrieden sind Sie mit Ihren Wohnbedingungen?	1	2	3	4	5
**124**	Wie zufrieden sind Sie mit Ihren Möglichkeiten, Gesundheitsdienste in Anspruch nehmen zu können?	1	2	3	4	5
**125**	Wie zufrieden sind Sie mit den Beförderungsmitteln, die Ihnen zur Verfügung stehen?	1	2	3	4	5
	**Bei der folgenden Frage geht es darum, wie oft sich <u>während der letzten zwei Wochen</u> bei Ihnen negative Gefühle eingestellt haben, wie zum Beispiel Angst oder Traurigkeit.**					
		niemals	nicht oft	zeitweilig	oftmals	immer
**126**	Wie häufig haben Sie negative Gefühle wie Traurigkeit, Verzweiflung, Angst oder Depression?	1	2	3	4	5
	PROG: Auf jeder Seite mit diesen Items<br>Hogrefe Copyright					

3. Gesundheitliche Situation (generisch)

Seitenüberschrift: Gesundheit & Lebensqualität

Stationäre Aufenthalte (generisch)
Quelle: eigen

PROG: kein Filter

Bitte geben Sie uns noch ein paar weitere Angaben zu Ihrem Gesundheitszustand.

Bitte denken Sie daran, falls Sie den Fragebogen stellvertretend für jemanden ausfüllen, alle Fragen aus Sicht der erkrankten Person zu beantworten.

201 **Wie viele Nächte waren Sie in den vergangenen 12 Monaten zur stationären Behandlung in einem Krankenhaus?**

____ Nächte (PROG: Wertebereich 0-366)

Komorbiditäten
Quelle: DEAS + Ergänzung um psychische Störungen

PROG: kein Filter

	**Hat Ihnen ein Arzt oder eine Ärztin schon einmal gesagt, dass Sie unter einer der hier aufgeführten Krankheiten leiden?**		
	**Erkrankung**	**ja**	**nein**
**203**	Diabetes, hohe Blutzuckerwerte	1	0
**204**	Herz- oder Kreislauferkrankung (z. B. Bluthochdruck, Herzinfarkt)	1	0
**205**	Schlaganfall	1	0
**206**	Durchblutungsstörungen	1	0
**207**	Gelenk-, Knochen-, Bandscheiben- oder Rückenleiden (z. B. Arthrose, Arthritis oder Rheuma)	1	0
**208**	Chronische Lungenerkrankung (z. B. Asthma, chronische Bronchitis, Lungenemphysem)	1	0
**209**	Krebserkrankung, bösartiger Tumor (einschließlich Leukämie)	1	0
**210**	Magen- oder Darmerkrankung	1	0
**211**	Blasenleiden	1	0
**212**	Gallen-, Leber- oder Nierenleiden	1	0
**213**	Parkinsonerkrankung	1	0
**214**	Demenz	1	0
**215**	Depression	1	0
**216**	Schizophrenie	1	0
**217**	Angststörung	1	0

**218**	Suchterkrankung	1	0

4. Gesundheitliche Situation (indikationsspezifisch)

Seitenüberschrift: Gesundheit & Lebensqualität

Fragen zur Erkrankung

Quelle: eigen

PROG:

Teilweise Filter nach Indikation und Gruppe

[Monate]: die letzten 3 vollen Kalendermonate einfügen

PROG: Extra Seite mit folgendem Text, nicht fett und größere Schrift

PROG: Gruppe = 1 & Indikation = 1 | 2 | 4
Sie nehmen aufgrund einer seelischen Erkrankung an dem neuen Versorgungsmodell (NPPV) teil. Wir möchten im Folgenden gern mehr über Ihre Erfahrungen mit dieser Erkrankung erfahren.

Es geht darum, wie Sie Ihre Krankheit erleben und wie es Ihnen mit Ihrer seelischen Erkrankung im Alltag geht. Uns ist dabei Ihre persönliche Einschätzung wichtig. Es gibt keine richtigen oder falschen Angaben.

PROG: Gruppe = 1 & Indikation = 3 | 5 | 6 | 7
Sie nehmen aufgrund einer Erkrankung des Nervensystems an dem neuen Versorgungsmodell (NPPV) teil. Wir möchten im Folgenden gern mehr über Ihre Erfahrungen mit dieser Erkrankung erfahren.

Es geht darum, wie Sie Ihre Krankheit erleben und wie es Ihnen mit Ihrer Erkrankung des Nervensystems im Alltag geht. Uns ist dabei Ihre persönliche Einschätzung wichtig. Es gibt keine richtigen oder falschen Angaben.

PROG: Gruppe = 2 & Indikation = 1 | 2 | 4
Wir haben Sie aufgrund einer seelischen Erkrankung zu dieser Befragung eingeladen. Wir möchten im Folgenden gern mehr über Ihre Erfahrungen mit dieser Erkrankung erfahren.

Es geht darum, wie Sie Ihre Krankheit erleben und wie es Ihnen mit Ihrer seelischen Erkrankung im Alltag geht. Uns ist dabei Ihre persönliche Einschätzung wichtig. Es gibt keine richtigen oder falschen Angaben.

PROG: Gruppe = 2 & Indikation = 3 | 5 | 6 | 7
Wir haben Sie aufgrund einer Erkrankung des Nervensystems zu dieser Befragung eingeladen. Wir möchten im Folgenden gern mehr über Ihre Erfahrungen mit dieser Erkrankung erfahren.

Es geht darum, wie Sie Ihre Krankheit erleben und wie es Ihnen mit Ihrer Erkrankung des Nervensystems im Alltag geht. Uns ist dabei Ihre persönliche Einschätzung wichtig. Es gibt keine richtigen oder falschen Angaben.

PROG: Gruppe 1 & Indikation = 1 | 2 | 4

Sie nehmen aufgrund einer seelischen Erkrankung an dem neuen Versorgungsmodell (NPPV) teil. Die folgenden Fragen beziehen sich daher auf Ihre Erfahrungen mit dieser Erkrankung.

PROG: Gruppe 1 & Indikation = 3 | 5 | 6 | 7

Sie nehmen aufgrund einer Erkrankung des Nervensystems an dem neuen Versorgungsmodell (NPPV) teil. Die folgenden Fragen beziehen sich daher auf Ihre Erfahrungen mit dieser Erkrankung.

PROG: Gruppe 2 & Indikation = 1 | 2 | 4

Wir haben Sie aufgrund einer seelischen Erkrankung zu dieser Befragung eingeladen. Die folgenden Fragen beziehen sich daher auf Ihre Erfahrungen mit dieser Erkrankung.

PROG: Gruppe 1 & Indikation = 3 | 5 | 6 | 7

Wir haben Sie aufgrund einer Erkrankung des Nervensystems zu dieser Befragung eingeladen. Die folgenden Fragen beziehen sich daher auf Ihre Erfahrungen mit dieser Erkrankung.

301 PROG: Indikation = 1 | 2 | 4

Wie lange leiden Sie bereits unter dieser seelischen Erkrankung?

PROG: Indikation = 3 | 5 | 6 | 7

Wie lange leiden Sie bereits unter dieser Erkrankung des Nervensystems?

_____ Jahr(e) _____ Monat(e) (PROG: Wertebereich 0-11)

302 PROG: kein Filter

Inwiefern hatten Sie <u>in den letzten 2 Wochen</u> aufgrund dieser Erkrankung körperliche und/oder seelische Beschwerden?

keine	leichte	mittlere	große
1	2	3	4

303 **Wie sehr fühlten Sie sich <u>in den letzten 2 Wochen</u> durch diese Erkrankung in Ihrem Alltag belastet?**

überhaupt nicht	kaum	etwas	ziemlich	sehr
1	2	3	4	5

304 **Wir möchten gern von Ihnen wissen, wie sich Ihre Erkrankung in der letzten Zeit verändert hat. Die folgenden Fragen beziehen sich daher auf <u>die letzten 3 Monate</u>, das heißt auf** [PROG: Monate]**.**

Hat sich Ihre Erkrankung <u>in den letzten 3 Monaten</u> verbessert, verschlechtert oder ist sie gleich geblieben?

deutlich verschlechtert	verschlechtert	gleich geblieben	verbessert	deutlich verbessert
1	2	3	4	5

305 PROG: Indikation = 1 | 2 | 4

Nehmen Sie zurzeit Medikamente gegen die seelische Erkrankung ein?

PROG: Indikation = 3 | 5 | 6 | 7

Nehmen Sie zurzeit Medikamente gegen die Erkrankung des Nervensystems ein?

❑... ja (1) ❑... nein (0)

5. Lebensqualität (indikationsspezifisch)

Seitenüberschrift: Gesundheit & Lebensqualität

Q-LES-Q 18 (Depression; Schizophrenie, schizotype oder wahnhafte Störungen, bipolare Störungen; Traumafolgestörungen)

Quelle: Ritsner, M., Kurs, R., Gibel, A., Ratner, Y. & Endicott, J. (2005). Validity of an abbreviated Quality of Life Enjoyment and Satisfaction Questionnaire (Q-LES-Q-18) for schizophrenia, schizoaffective,and mood disorder patients. Quality of Life research, 14, 1693-1703.

Endicott J, Nee J, Harrison W, Blumenthal R. Quality of Life Enjoyment and Satisfaction Questionnaire: a new measure. Psychopharmacol Bull. 1993;29(2):321-6

Rohenkohl, A., Ruppelt, F., Gallinat, J., Karow, A., Lüdecke, D., Nawara, L. A., ... Lambert, M. (2015). Erfassung der Lebensqualität bei PsychosepatientInnen – psychometrische Analyse des Q-LES-Q-18 Fragebogens

Lizenzfrei in Ritsner publiziert

Deutsche Übersetzung mit Rückübersetzung selbst gemacht

PROG: Indikation = 1 | 2 | 4 (Depression; Schizophrenie, schizotype oder wahnhafte Störungen, bipolare Störungen; Traumafolgestörungen)

PROG: Indikation = 1 | 2 | 4

In den folgenden Fragen geht es darum, wie es Ihnen <u>in den letzten zwei Wochen</u> in verschiedenen Bereichen Ihres Lebens ergangen ist.

	**Wie häufig…**	nie	selten	manch mal	häufig	immer
**1401**	… waren Sie bei sehr guter körperlicher Gesundheit?	1	2	3	4	5
**1402**	… waren Sie frei von Sorgen über Ihre körperliche Gesundheit?	1	2	3	4	5
**1403**	… fühlten Sie sich körperlich gut?	1	2	3	4	5
**1404**	… fühlten Sie sich voller Energie und Vitalität?	1	2	3	4	5
**1405**	… waren Sie zufrieden mit Ihrem Leben?	1	2	3	4	5
**1406**	… fühlten Sie sich glücklich oder fröhlich?	1	2	3	4	5
**1407**	… fühlten Sie sich in der Lage mit anderen zu kommunizieren?	1	2	3	4	5
**1408**	… fühlten Sie sich in der Lage sich zu Fuß, mit dem Auto, Bus, Bahn oder Fahrrad fortzubewegen, um Erledigungen zu machen?	1	2	3	4	5
**1409**	… fühlten Sie sich in der Lage, sich um sich selbst zu kümmern?	1	2	3	4	5
**1410**	… sind Sie Ihren Freizeitaktivitäten nachgegangen?	1	2	3	4	5

**1411**	… haben Sie sich auf die Freizeitaktivitäten konzentriert und ihnen Aufmerksamkeit geschenkt?	1	2	3	4	5
**1412**	Wenn bei Ihren Freizeitaktivitäten ein Problem auftauchte, wie oft konnten Sie es lösen oder damit ohne übermäßigen Stress umgehen?	1	2	3	4	5
**1413**	… haben Sie sich auf ein Zusammentreffen mit Freunden oder Verwandten gefreut?	1	2	3	4	5
**1414**	… hat es Ihnen Spaß gemacht, mit Kollegen oder Nachbarn zu sprechen?	1	2	3	4	5
**1415**	… haben Sie Zuneigung gegenüber einer oder mehrerer Personen gespürt?	1	2	3	4	5
**1416**	… haben Sie mit anderen Menschen gescherzt oder gelacht?	1	2	3	4	5
**1417**	… waren Sie für Ihre Freunde oder Verwandten da, wenn diese Sie gebraucht haben?	1	2	3	4	5

DEMQoL (Demenz)

Quelle: Smith, S. C., Lamping, D. L., Banerjee, S., Harwood, R., Foley, B., Smith, P., ... & Mann, A. (2005). Measurement of health-related quality of life for people with dementia: development of a new instrument (DEMQOL) and an evaluation of current methodology. Health Technology Assessment (Winchester, England), 9(10), 1-93.

Berwig, M., Leicht, H., & Gertz, H. J. (2009). Critical evaluation of self-rated quality of life in mild cognitive impairment and Alzheimer's disease — Further evidence for the impact of anosognosia and global cognitive impairment. JNHA - The Journal of Nutrition, Health and Aging, 13(3), 226–230. doi:10.1007/s12603-009-0063-4

Berwig, M., Leicht, H., Hartwig, K., & Gertz, H. J. (2011). Self-rated quality of life in mild cognitive impairment and Alzheimer's disease: The problem of affective distortion. GeroPsych: The Journal of Gerontopsychology and Geriatric Psychiatry, 24(1), 45–51. doi:http://dx.doi.org/10.1024/1662-9647/a000029

Lizenzfrei, deutsche Version von Brewig erhalten; Item nach genereller LQ gestrichen, da schon in WHOQoL

PROG: Indikation = 3 (Demenz)

PROG: Indikation = 3

Im Folgenden möchten wir Sie zu Beschwerden befragen, die häufig im Alter auftreten. Beispiele sind Gedächtnisstörungen, Schwierigkeiten im alltäglichen Leben oder eine gedrückte Stimmung. Wir möchten Sie auch fragen, wie häufig die Beschwerden waren und wie besorgt Sie über die Beschwerden waren. Es gibt keine richtigen und keine falschen Antworten.

	**Zunächst möchten wir Sie zu Ihren Gefühlen fragen. Wie oft haben Sie die folgenden Gefühle in den letzten 2 Wochen erlebt?**	nie	selten	manchmal	häufig
**3401**	Heiterkeit	1	2	3	4
**3402**	Angst oder Sorge	1	2	3	4
**3403**	Haben Sie das Leben genossen?	1	2	3	4
**3404**	Frustration	1	2	3	4
**3405**	Selbstsicherheit	1	2	3	4
**3406**	Voller Energie	1	2	3	4
**3407**	Traurigkeit	1	2	3	4
**3408**	Einsamkeit	1	2	3	4
**3409**	Verzweiflung	1	2	3	4
**3410**	Lebhaftigkeit	1	2	3	4
**3411**	Gereiztheit	1	2	3	4
**3412**	Überdruss	1	2	3	4

3413	Unfähigkeit	1	2	3	4
	**Jetzt folgen Fragen zu Ihrem Gedächtnis. Wie oft waren Sie <u>in den letzten 2 Wochen</u> besorgt wegen der folgenden Beschwerden?**	1	2	3	4
3414	Besorgt, weil Sie kurz zurückliegende Ereignisse vergessen	1	2	3	4
3415	Besorgt, weil Sie vergessen, wer bestimmte Personen sind	1	2	3	4
3416	Besorgt, weil Sie vergessen, welcher Wochentag ist	1	2	3	4
3417	Besorgt, weil Ihre Gedanken durcheinander sind	1	2	3	4
3418	Besorgt, weil Sie keine Entscheidungen treffen können	1	2	3	4
3419	Besorgt wegen Konzentrationsstörungen				
	**Jetzt folgen Fragen über Ihren Alltag. Wie oft waren Sie <u>in den letzten 2 Wochen</u> besorgt über folgende Lebensumstände?**	1	2	3	4
3420	Besorgt, weil Sie nicht genug Gesellschaft hatten	1	2	3	4
3421	Besorgt, weil Sie nicht wussten, wie Sie mit anderen Menschen in Ihrer Umgebung umgehen sollen	1	2	3	4
3422	Besorgt, weil Sie nicht die Zuneigung bekamen, die Sie sich gewünscht haben	1	2	3	4
3423	Besorgt, weil Ihnen nicht zugehört wurde	1	2	3	4
3424	Besorgt, weil Sie sich nicht verständlich machen konnten	1	2	3	4
3425	Besorgt, weil Sie nicht die Hilfe bekommen haben, die Sie gebraucht hätten	1	2	3	4
3426	Besorgt, es nicht rechtzeitig auf die Toilette zu schaffen	1	2	3	4
3427	Besorgt, weil Sie sich in ihrer eigenen Haut nicht wohl fühlten	1	2	3	4
3428	Besorgt über Ihren allgemeinen Gesundheitszustand	1	2	3	4

MSIS

Quelle: Hobart, J., Lamping, D., Fitzpatrick, R., Riazi, A., Thompson, A. (2001). The Multiple Sclerosis Impact Scale (MSIS-29): A new patient-based outcome measure. Brain, 124, 962-973.
Schönberg, P. (2012). Validierung der deutschen Version der Multiple Sclerosis Impact Scale (MSIS-29). Dissertation. Universitätsklinikum Hamburg-Eppendorf

lizenzfrei

PROG: Indikation = 5 (Multiple Sklerose)

PROG: Indikation = 5

In den folgenden Fragen geht es um den Einfluss Ihrer Multiple Sklerose Erkrankung auf Ihr tägliches Leben.

Bitte kreuzen Sie für jede Aussage an, was am besten auf Ihre Situation zutrifft.

	**Wie schwer fiel es Ihnen in den letzten zwei Wochen …**	gar nicht	ein bisschen	mäßig	ziemlich	sehr
**5401**	körperlich anstrengende Dinge zu tun?	1	2	3	4	5
**5402**	Dinge fest anzufassen (z. B. Hahn aufdrehen)?	1	2	3	4	5
**5403**	Dinge zu tragen?	1	2	3	4	5
	**Hatten Sie in den letzten zwei Wochen…**					
**5404**	Probleme mit dem Gleichgewicht?	1	2	3	4	5
**5405**	Schwierigkeiten, sich in der Wohnung zu bewegen?	1	2	3	4	5
**5406**	das Gefühl ungeschickt zu sein?	1	2	3	4	5
**5407**	ein Steifigkeitsgefühl?	1	2	3	4	5
**5408**	schwere Arme und / oder Beine?	1	2	3	4	5
**5409**	Zittern der Arme oder Beine?	1	2	3	4	5
**5410**	Krämpfe der Extremitäten?	1	2	3	4	5
**5411**	das Gefühl, dass ihr Körper nicht tat, was sie wollten?	1	2	3	4	5
**5412**	Beeinträchtigung im sozialen und Freizeitleben zu Hause?	1	2	3	4	5
**5413**	Probleme mit den Händen bei Alltagstätigkeiten?	1	2	3	4	5
**5414**	Probleme sich fortzubewegen (Auto, Bus, Taxi, Zug)?	1	2	3	4	5
**5415**	länger gebraucht, Dinge zu tun?	1	2	3	4	5
**5416**	Schwierigkeiten, Dinge spontan zu machen?	1	2	3	4	5

5417	das Gefühl, ganz schnell zur Toilette zu müssen?	1	2	3	4	5
5418	sich allgemein unwohl gefühlt?	1	2	3	4	5
5419	Schlafprobleme?	1	2	3	4	5
5420	sich geistig / mental müde gefühlt?	1	2	3	4	5
5421	Sorgen bezogen auf ihre MS?	1	2	3	4	5
5422	sich angespannt und ängstlich gefühlt?	1	2	3	4	5
5423	sich ungeduldig und aufbrausend gefühlt?	1	2	3	4	5
5424	Konzentrationsprobleme?	1	2	3	4	5
5425	keine Zuversicht?	1	2	3	4	5
5426	sich traurig / depressiv gefühlt?	1	2	3	4	5
	**Waren Sie in den letzten zwei Wochen…**					
5427	davon abhängig, dass andere Dinge für sie erledigten?	1	2	3	4	5
5428	gezwungen, zu Hause zu bleiben?	1	2	3	4	5
5429	Gezwungen, die Zeit für Arbeit oder Alltagsaktivitäten einzuschränken?	1	2	3	4	5

PROG: Auf jeder Seite mit diesen Items:

®2000 Neurological Outcome Measures Unit

PDQ-39 (Parkinson)

Quelle: Berger, K., Broll, S., Winkelmann, J., Heberlein, I., Müller, T., Ries, V. für die FAQT-Studienzentren (1999). Untersuchung zur Reliabilität der deutschen Version des PDQ-39: Ein krankheitsspezifischer Fragebogen zur Erfassung der Lebensqualität von Parkinson-Patienten. Aktuelle Neurologie, 26, 180-184.

lizenzfrei

PROG: Indikation = 6 (Parkinson)

PROG: Indikation = 6

In den folgenden Fragen geht es darum, wie es Ihnen in verschiedenen Bereichen in Ihrem Leben ergangen ist.

	**Wie oft haben Sie <u>in den letzten 2 Wochen</u> wegen Ihrer Parkinsonerkrankung…**	niemals	selten	manchmal	häufig	Immer oder kann ich überhaupt nicht
**6401**	… Schwierigkeiten gehabt, Freizeitaktivitäten, die Sie gern machen würden, auszuüben?	1	2	3	4	5
**6402**	… Schwierigkeiten gehabt, Ihren Haushalt zu versorgen (z. B. handwerkliche Tätigkeiten, Hausarbeiten, Kochen)?	1	2	3	4	5
**6403**	… Schwierigkeiten gehabt, Einkaufstaschen zu tragen?	1	2	3	4	5
**6404**	… Probleme gehabt, ungefähr 1 km zu gehen?	1	2	3	4	5
**6405**	… Probleme gehabt, ungefähr 100 m zu gehen?	1	2	3	4	5
**6406**	… Probleme gehabt, sich im Haus so zu bewegen, wie Sie wollten?	1	2	3	4	5
**6407**	… Probleme gehabt, sich in der Öffentlichkeit zu bewegen?	1	2	3	4	5
**6408**	… eine Begleitperson gebraucht, um sich außer Haus zu bewegen?	1	2	3	4	5
**6409**	… Angst oder Sorgen gehabt, dass Sie in der Öffentlichkeit hinfallen?	1	2	3	4	5
**6410**	… das Gefühl gehabt, mehr an das Haus gebunden zu sein, als Ihnen lieb wäre?	1	2	3	4	5
**6411**	… Schwierigkeiten gehabt, sich selbst zu waschen?	1	2	3	4	5
**6412**	… Schwierigkeiten gehabt, sich selbst anzuziehen?	1	2	3	4	5
**6413**	… Probleme gehabt, Knöpfe zu schließen oder Schnürsenkel zu binden?	1	2	3	4	5
**6414**	… Probleme gehabt, deutlich zu schreiben?	1	2	3	4	5
**6415**	… Schwierigkeiten gehabt, Ihr Essen klein zu schneiden?	1	2	3	4	5
**6416**	… Schwierigkeiten gehabt, ein Getränk zu halten, ohne es zu verschütten?	1	2	3	4	5

6417	… sich niedergeschlagen oder deprimiert gefühlt?	1	2	3	4	5
6418	… sich isoliert oder einsam gefühlt?	1	2	3	4	5
6419	… sich verärgert oder verbittert gefühlt?	1	2	3	4	5
6420	… sich den Tränen nahe gefühlt?	1	2	3	4	5
6421	… sich ängstlich gefühlt?	1	2	3	4	5
6422	… sich Sorgen über Ihre Zukunft gemacht?	1	2	3	4	5
6423	… das Gefühl gehabt, Ihre Parkinsonerkrankung vor anderen verheimlichen zu müssen?	1	2	3	4	5
6424	… Situationen vermieden, die mit Essen oder Trinken in der Öffentlichkeit verbunden waren?	1	2	3	4	5
6425	… sich in der Öffentlichkeit wegen Ihrer Parkinsonerkrankung geschämt?	1	2	3	4	5
6426	… sich Sorgen über die Reaktionen anderer Ihnen gegenüber gemacht?	1	2	3	4	5
6427	… Probleme im Verhältnis mit Ihnen nahe stehenden Menschen gehabt?	1	2	3	4	5
6428	… nicht die Unterstützung erhalten, die Sie von Ihrem (Ehe-)Partner benötigt hätten?	1	2	3	4	5
6429	… nicht die Unterstützung erhalten, die Sie von Ihren Verwandten oder engen Freunden benötigt hätten?	1	2	3	4	5
6430	… das Problem gehabt, tagsüber unerwartet einzuschlafen?	1	2	3	4	5
6431	… Probleme gehabt, sich zu konzentrieren (z. B. beim Lesen oder beim Fernsehen)?	1	2	3	4	5
6432	… das Gefühl gehabt, dass Sie ein schlechtes Gedächtnis hätten?	1	2	3	4	5
6433	… schlechte Träume oder Halluzinationen gehabt?	1	2	3	4	5
6434	… Schwierigkeiten mit dem Sprechen gehabt?	1	2	3	4	5
6435	… sich außer Stande gefühlt, mit anderen zu kommunizieren?	1	2	3	4	5
6436	… den Eindruck gehabt, von anderen nicht beachtet zu werden?	1	2	3	4	5
6437	… schmerzhafte Muskelkrämpfe gehabt?	1	2	3	4	5
6438	… Schmerzen in den Gelenken oder anderen Körperteilen gehabt?	1	2	3	4	5
6439	… sich unangenehm heißt oder kalt gefühlt?	1	2	3	4	5

SA-SIP30 (Schlaganfall)

Quelle: van Straten, A., de Haan, R. J., Limburg, M., Schuling, J., Bossuyt, P. M., van de Bos, G. A. M. (1997). A Stroke-Adapted 30-Item Version of the Sickness Impact Profile to Assess Quality of Life (SA-SIP30). Stroke, 28, 2155-2161.

Hütter, B. O. (2002). Sickness Impact Profile (SIP) -German version. In S. Salek (Ed.), Compendium of qua/ity of /ife instruments. Chichester, West Sussex: Wiley.

Lizenzfrei – deutsche Version von Hütter bekommen

PROG: Indikation = 7 (Schlaganfall)

PROG: Indikation = 7

Dieser Fragebogen dient dazu festzustellen, welche Beschwerden Sie im Augenblick haben.

Bitte lesen Sie sich alle Sätze genau durch und antworten Sie nur bei solchen Beschwerden mit "ja", die Sie am heutigen Tag haben und die sich auf Ihren Gesundheitszustand beziehen.

		ja	nein
**7401**	Ich kann schwierige Bewegungen nur mit Hilfe machen, wie z. B. in ein Auto oder eine Badewanne ein- und aussteigen.	1	0
**7402**	Meine Hände oder Finger kann ich nur mit Einschränkungen oder Schwierigkeiten gebrauchen.	1	0
**7403**	Wenn ich ins Bett gehen will oder aufstehen möchte, z. B. von einem Stuhl, so muss ich mich an etwas festhalten oder einen Stock benutzen.	1	0
**7404**	Ich habe Schwierigkeiten, mir alleine Schuhe, Strümpfe oder Socken anzuziehen.	1	0
**7405**	Ich kann mich nur anziehen, wenn mir jemand hilft.	1	0
**7406**	Ich kann mich für die Probleme von anderen nicht mehr so interessieren, z. B. höre ich nicht zu, wenn sie mir von ihren Problemen erzählen, oder ich biete ihnen keine Hilfe an.	1	0
**7407**	Ich bin oft ungehalten zu denjenigen, die mich umgeben, z. B. schneide ich ihnen das Wort ab, gebe scharfe Antworten oder kritisiere leicht.	1	0
**7408**	Ich zeige weniger Zuneigung.	1	0
**7409**	Ich unternehme weniger soziale Aktivitäten mit Gruppen von Leuten.	1	0
**7410**	Ich spreche weniger mit denjenigen, die mich umgeben.	1	0
**7411**	Ich bleibe die meiste Zeit zu Hause.	1	0
**7412**	Ich gehe nicht in die Stadt.	1	0
**7413**	Ich bewege mich ohne fremde Hilfe nicht in der Dunkelheit oder in unbeleuchteten Plätzen.	1	0

**7414**	Ich nehme nur noch dann an einem Gespräch teil, wenn ich der anderen Person sehr nahe stehe oder zu ihr schaue.	1	0
**7415**	Ich habe Schwierigkeiten in der Aussprache, z. B. stottere ich, bleibe stecken, stammle oder kann die Worte nicht mehr deutlich aussprechen.	1	0
**7416**	Ich kann nicht klar sprechen, wenn ich unter Stress bin.	1	0
**7417**	Ich sage, wie schlecht oder nutzlos ich bin, z. B. dass ich für andere eine Last bin.	1	0
**7418**	Ich lache oder weine plötzlich.	1	0
**7419**	Ich bin gereizt und ungeduldig mit mir selbst, z. B. spreche ich schlecht über mich, verfluche mich, gebe mir die Schuld, wenn etwas passiert.	1	0
**7420**	Ich bekomme plötzliche Angstzustände.	1	0
**7421**	Ich kann die normale Hausarbeit, die ich eigentlich tun müsste, überhaupt nicht mehr tun.	1	0
**7422**	Ich gehe nicht mehr einkaufen, so wie ich es normalerweise tun würde.	1	0
**7423**	Ich kann nicht mehr die Wohnung oder das Haus putzen, so wie ich es normalerweise tun würde.	1	0
**7424**	Ich kann die Wäsche nicht mehr waschen, so wie ich es normalerweise tun würde.	1	0
**7425**	Ich bin durcheinander und fange mehrere Sachen gleichzeitig an.	1	0
**7426**	Ich mache mehr Fehler als üblich.	1	0
**7427**	Ich habe Schwierigkeiten, Tätigkeiten auszuführen, die Konzentration und Denken erfordern.	1	0
**7428**	Ich gehe keine Steigungen mehr und / oder vermeide abschüssige Strecken.	1	0
**7429**	Ich kann mich nur noch mit Hilfe eines Spazierstocks, Krücken, Wänden oder Möbeln fortbewegen.	1	0
**7430**	Ich laufe langsamer.	1	0

6. Versorgungsqualität bisherige Versorgung (indikationsspezifisch)

Seitenüberschrift: Versorgungsqualität

Qualität / Zufriedenheit / Bezugsarzt
Quelle: eigen

PROG: Extra Seite mit folgendem Text, nicht fett und größere Schrift

PROG: Gruppe = 1 & Indikation = 1 | 2 | 4
Auf den folgenden Seiten möchten wir gern mehr über die Behandlung Ihrer seelischen Erkrankung erfahren, bevor Sie an dem neuen Versorgungsmodell (NPPV) teilgenommen haben.

Es geht darum, wie Sie Ihre Behandlung erlebt haben und wie zufrieden Sie mit Ihrer Behandlung waren. Wir sind dabei an Ihrer persönlichen Einschätzung interessiert. Es gibt keine richtigen oder falschen Angaben.
Bitte denken Sie daran, falls Sie den Fragebogen stellvertretend für jemanden ausfüllen, alle Fragen aus Sicht der erkrankten Person zu beantworten.

PROG: Gruppe = 1 & Indikation = 3 | 5 | 6 | 7
Auf den folgenden Seiten möchten wir gern mehr über die Behandlung Ihrer Erkrankung des Nervensystems erfahren, bevor Sie an dem neuen Versorgungsmodell (NPPV) teilgenommen haben.

Es geht darum, wie Sie Ihre Behandlung erlebt haben und wie zufrieden Sie mit Ihrer Behandlung waren. Wir sind dabei an Ihrer persönlichen Einschätzung interessiert. Es gibt keine richtigen oder falschen Angaben.
Bitte denken Sie daran, falls Sie den Fragebogen stellvertretend für jemanden ausfüllen, alle Fragen aus Sicht der erkrankten Person zu beantworten.

PROG: Gruppe = 2 & Indikation = 1 | 2 | 4
Auf den folgenden Seiten möchten wir gern mehr über die Behandlung Ihrer seelischen Erkrankung erfahren.

Es geht darum, wie Sie Ihre Behandlung erlebt haben und wie zufrieden Sie mit Ihrer Behandlung waren. Wir sind dabei an Ihrer persönlichen Einschätzung interessiert. Es gibt keine richtigen oder falschen Angaben.
Bitte denken Sie daran, falls Sie den Fragebogen stellvertretend für jemanden ausfüllen, alle Fragen aus Sicht der erkrankten Person zu beantworten.

PROG: Gruppe = 2 & Indikation = 3 | 5 | 6 | 7
Auf den folgenden Seiten möchten wir gern mehr über die Behandlung Ihrer Erkrankung des Nervensystems erfahren.

Es geht darum, wie Sie Ihre Behandlung erlebt haben und wie zufrieden Sie mit Ihrer Behandlung waren. Wir sind dabei an Ihrer persönlichen Einschätzung interessiert. Es gibt keine richtigen oder falschen Angaben.
Bitte denken Sie daran, falls Sie den Fragebogen stellvertretend für jemanden ausfüllen, alle Fragen aus Sicht der erkrankten Person zu beantworten.

PROG: teilweise Filter nach Indikation und nach Gruppe

PROG: Gruppe = 1 & Indikation = 1 | 2 | 4, auf jeder Seite bis Frage 515_5 anzeigen

Die folgenden Fragen beziehen sich auf die Behandlung der seelischen Erkrankung, bevor Sie an dem neuen Versorgungsmodell (NPPV) teilgenommen haben.

PROG: Gruppe = 1 & Indikation = 3 | 5 | 6 | 7, auf jeder Seite bis Frage 515_5 anzeigen

Die folgenden Fragen beziehen sich auf die Behandlung der Erkrankung des Nervensystems, bevor Sie an dem neuen Versorgungsmodell (NPPV) teilgenommen haben.

PROG: Gruppe = 2 & Indikation = 1 | 2 | 4, auf jeder Seite bis Frage 515_5 anzeigen

Die folgenden Fragen beziehen sich auf die bisherige Behandlung der seelischen Erkrankung.

PROG: Gruppe = 2 & Indikation = 3 | 5 | 6 | 7, auf jeder Seite bis Frage 515_5 anzeigen

Die folgenden Fragen beziehen sich auf die bisherige Behandlung der Erkrankung des Nervensystems.

PROG: alle

Im Folgenden ist mit „Arzt / (Psycho-)therapeut / Neurologe / Psychiater" immer auch die weibliche Form „Ärztin / (Psycho-)Therapeutin / Neurologin / Psychiaterin" eingeschlossen.

500 PROG: Indikation = 1 | 2 | 4

Bei welchem Arzt / Psychotherapeuten waren Sie in den letzten 12 Monaten mit Ihrer seelischen Erkrankung vorwiegend, d.h. die meiste Zeit, in Behandlung?

PROG: Indikation = 3 | 5 | 6 | 7

Bei welchem Arzt / Psychotherapeuten waren Sie in den letzten 12 Monaten mit Ihrer Erkrankung des Nervensystems vorwiegend, d. h. die meiste Zeit, in Behandlung?

❑ Hausarzt (1)

❑ Psychiater (2)

❑ Neurologe oder Nervenarzt (3)

❑ Psychotherapeut (4)

❑ anderer Arzt / Therapeut (5)

nämlich: ___________________

509 PROG: Indikation = 1 | 2 | 4

Nahmen Sie für Ihre Behandlung in regelmäßigen Abständen Termine bei diesem Arzt / Psychotherapeuten wahr?

PROG: Indikation = 3 | 5 | 6 | 7

Nahmen Sie für Ihre Behandlung in regelmäßigen Abständen Termine bei diesem Arzt wahr?

❑ ja (1) ❑ nein (0)

509_1 PROG: Frage 509 = 1 (ja)

Wie häufig nahmen Sie regelmäßige Behandlungstermine wahr?

jede Woche	alle 2 Wochen	jeden Monat	alle 2-3 Monate	alle 4-6 Monate	seltener
1	2	3	4	5	**6**

PROG: Indikation = 1 | 2 | 4; auf jeder Seite bis Frage 515_5 anzeigen

Bitte beziehen Sie die folgenden Fragen auf diesen Arzt / Psychotherapeuten, bei dem Sie vorwiegend in Behandlung waren.

PROG: Indikation = 3 | 5 | 6 | 7; auf jeder Seite bis Frage 515_5 anzeigen

Bitte beziehen Sie die folgenden Fragen auf diesen Arzt, bei dem Sie vorwiegend in Behandlung waren.

PROG: kein Filter

Wie schätzen Sie die Qualität der bisherigen Behandlung in den letzten 12 Monaten ein?

501 PROG: Indikation = 1 | 2 | 4

In meiner bisherigen Behandlung nahm sich mein Arzt / Psychotherapeut immer genug Zeit für mich.

PROG: Indikation = 3 | 5 | 6 | 7

In meiner bisherigen Behandlung nahm sich mein Arzt immer genug Zeit für mich.

stimmt überhaupt nicht	stimmt nicht	weder noch	stimmt	stimmt voll und ganz
1	2	3	4	5

502 PROG: kein Filter

In meiner bisherigen Behandlung fühlte ich mich sehr gut betreut.

stimmt überhaupt nicht	stimmt nicht	weder noch	stimmt	stimmt voll und ganz
1	2	3	4	5

503 **Meine bisherige Behandlung deckte meine Bedürfnisse voll und ganz ab.**

stimmt überhaupt nicht	stimmt nicht	weder noch	stimmt	stimmt voll und ganz
1	2	3	4	5

503_1 PROG: Indikation = 1 | 2 | 4

In meiner bisherigen Behandlung hat mir mein Arzt / Psychotherapeut ausführlich erklärt, was ich bei Notfällen und Krisen, zum Beispiel wenn es mir plötzlich schlechter geht, tun soll.

PROG: Indikation = 3 | 5 | 6 | 7

In meiner bisherigen Behandlung hat mir mein Arzt ausführlich erklärt, was ich bei Notfällen und Krisen, zum Beispiel wenn es mir plötzlich schlechter geht, tun soll.

stimmt überhaupt nicht	stimmt nicht	weder noch	stimmt	stimmt voll und ganz
1	2	3	4	5

503_2 PROG: Indikation = 1 | 2 | 4

In meiner bisherigen Behandlung hat mich mein Arzt / Psychotherapeut ausführlich informiert, dass ich in Notfällen und Krisen schnell einen Termin bei ihm bekomme.

PROG: Indikation = 3 | 5 | 6 | 7

In meiner bisherigen Behandlung hat mich mein Arzt ausführlich informiert, dass ich in Notfällen und Krisen schnell einen Termin bei ihm bekomme.

stimmt überhaupt nicht	stimmt nicht	weder noch	stimmt	stimmt voll und ganz
1	2	3	4	5

504 PROG: Indikation = 1 | 2 | 4

In meiner bisherigen Behandlung erklärte mir mein Arzt / Psychotherapeut meinen Therapieverlauf einfach und verständlich.

PROG: Indikation = 3 | 5 | 6 | 7

In meiner bisherigen Behandlung erklärte mir mein Arzt meinen Therapieverlauf einfach und verständlich.

stimmt überhaupt nicht	stimmt nicht	weder noch	stimmt	stimmt voll und ganz
1	2	3	4	5

510 PROG: Indikation = 1 | 2 | 4

In meiner bisherigen Behandlung arbeiteten verschiedene Ärzte / Psychotherapeuten gut zusammen und informierten sich gegenseitig.

PROG: Indikation = 3 | 5 | 6 | 7

In meiner bisherigen Behandlung arbeiteten verschiedene Ärzte gut zusammen und informierten sich gegenseitig.

stimmt überhaupt nicht	stimmt nicht	weder noch	stimmt	stimmt voll und ganz
1	2	3	4	5

510_1 PROG: Indikation = 1 | 2 | 4

In meiner bisherigen Behandlung vermittelte mich mein Arzt / Psychotherapeut bei Bedarf an kompetente Stellen weiter (z. B. Physio- / Ergotherapie, anderer Facharzt).

PROG: Indikation = 3 | 5 | 6 | 7

In meiner bisherigen Behandlung vermittelte mich mein Arzt bei Bedarf an kompetente Stellen weiter (z. B. Physio- / Ergotherapie, anderer Facharzt).

stimmt überhaupt nicht	stimmt nicht	weder noch	stimmt	stimmt voll und ganz
1	2	3	4	5

505 PROG: Indikation = 1 | 2 | 4

In meiner bisherigen Behandlung empfahl mir mein Arzt / Psychotherapeut sinnvolle therapeutische Angebote.

PROG: Indikation = 3 | 5 | 6 | 7

In meiner bisherigen Behandlung empfahl mir mein Arzt sinnvolle therapeutische Angebote.

stimmt überhaupt nicht	stimmt nicht	weder noch	stimmt	stimmt voll und ganz
1	2	3	4	5

Gruppenangebote

506 PROG: Indikation = 1 | 2 | 4

Wurden Ihnen in Ihrer bisherigen Behandlung von Ihrem Arzt / Psychotherapeuten Gruppenangebote, die nicht in einem Krankenhaus stattfanden, zur Unterstützung der Therapie empfohlen?

PROG: Indikation = 3 | 5 | 6 | 7

Wurden Ihnen in Ihrer bisherigen Behandlung von Ihrem Arzt Gruppenangebote, die nicht in einem Krankenhaus stattfanden, zur Unterstützung der Therapie empfohlen?

❑ ja (1) ❑ nein (0)

Wie wurden Sie über das Gruppenangebot informiert?

506_01 PROG: Frage 506 = 1 (ja) & Indikation = 1 | 2 | 4

Mein Arzt / Psychotherapeut beschrieb mir ausführlich den Inhalt und Ablauf des Gruppenangebots.

PROG: Frage 506 = 1 (ja) & Indikation = 3 | 5 | 6 | 7

Mein Arzt beschrieb mir ausführlich den Inhalt und Ablauf des Gruppenangebots.

stimmt überhaupt nicht	stimmt nicht	weder noch	stimmt	stimmt voll und ganz
1	2	3	4	5

506_02 PROG: Frage 506 = 1 (ja) & Indikation = 1 | 2 | 4

Mein Arzt / Psychotherapeut erläuterte mir genau, warum das Gruppenangebot für mich hilfreich ist.

PROG: Frage 506 = 1 (ja) & Indikation = 3 | 5 | 6 | 7

Mein Arzt erläuterte mir genau, warum das Gruppenangebot für mich hilfreich ist.

stimmt überhaupt nicht	stimmt nicht	weder noch	stimmt	stimmt voll und ganz
1	2	3	4	5

506_03 PROG: Frage 506 = 1 (ja) & Indikation = 1 | 2 | 4

Mein Arzt / Psychotherapeut bzw. das Praxisteam unterstützte mich sehr dabei, das Gruppenangebot wahrzunehmen.

PROG: Frage 506 = 1 (ja) & Indikation = 3 | 5 | 6 | 7

Mein Arzt bzw. das Praxisteam unterstützte mich sehr dabei, das Gruppenangebot wahrzunehmen

stimmt überhaupt nicht	stimmt nicht	weder noch	stimmt	stimmt voll und ganz
1	2	3	4	5

PROG: Frage 506 = 1 (ja)

Welche Gruppenangebote wurden Ihnen empfohlen und hatten Sie diese wahrgenommen?

	Gruppenangebot	empfohlen	wahrgenommen
**506_11** **506_12**	Gruppenangebot für Betroffene	ja (1) / nein (0)	ja (1) / nein (0)
**506_21** **506_22**	Gruppenangebot für Angehörige	ja (1) / nein (0)	ja (1) / nein (0)

506_5 PROG: Frage 506 = 1 (ja) & mind. einmal (Frage 506_x1 = 1 (ja) & Frage 506_x2 = 0 (nein))

506_6 **Sie haben angegeben, dass Sie mindestens an einem Gruppenangebot, das Ihnen empfohlen wurde, nicht teilgenommen hatten.**

506_7 **Welche Gründe hatten Sie dafür?**

506_71 PROG: Mehrfachantwort möglich

506_8

506_8o

- ❑ Ich hatte kein Interesse daran. (1 genannt; 0 nicht genannt)
- ❑ Der Aufwand war mir zu groß. (1 genannt; 0 nicht genannt)
- ❑ Der Weg war mir zu weit. (1 genannt; 0 nicht genannt)
- ❑ Ich glaubte nicht, dass mir das Angebot hilft. (1 genannt; 0 nicht genannt)
- ❑ Ich möchte meine Probleme nicht vor anderen Patienten besprechen. (1 genannt; 0 nicht genannt)
- ❑ anderer Grund (1 genannt; 0 nicht genannt)

PROG: Frage 506_8 = 1 (anderer Grund)

Und zwar: _____________

PROG: Frage 506 = 1 (ja) & mind. einmal (Frage 506_x2 = 1 (ja))

Sie nahmen an mindestens einem Gruppenangebot teil. Bitte geben Sie an, inwiefern Sie den folgenden Aussagen zustimmen.

506_9 PROG: Frage 506 = 1 (ja) & mind. einmal (Frage 506_x2 = 1 (ja))

Das Gruppenangebot half mir sehr, im Alltag mit meiner Erkrankung zurecht zu kommen.

stimmt überhaupt nicht	stimmt nicht	weder noch	stimmt	stimmt voll und ganz
1	2	3	4	5

506_010 PROG: Frage 506 = 1 (ja) & mind. einmal (Frage 506_x2 = 1 (ja))

Durch das Gruppenangebot belastete mich meine Erkrankung deutlich weniger.

stimmt überhaupt nicht	stimmt nicht	weder noch	stimmt	stimmt voll und ganz
1	2	3	4	5

506_011 PROG: Frage 506 = 1 (ja) & mind. einmal (506_x2 = 1 (ja))

Durch das Gruppenangebot verstand ich meine Erkrankung besser.

stimmt überhaupt nicht	stimmt nicht	weder noch	stimmt	stimmt voll und ganz
1	2	3	4	5

506_012 PROG: Frage 506 = 1 (ja) & mind. einmal (Frage 506_x2 = 1 (ja))

Der Austausch mit anderen Betroffenen beim Gruppenangebot half mir sehr.

stimmt überhaupt nicht	stimmt nicht	weder noch	stimmt	stimmt voll und ganz
1	2	3	4	5

Onlineangebote

507 PROG: kein Filter

Wurde Ihnen in ihrer bisherigen Behandlung Online-Selbsthilfe zur Unterstützung der Therapie empfohlen?

❑ ja (1) ❑ nein (0)

Wie wurden Sie über die Online-Selbsthilfe informiert?

507_01 PROG: Frage 507 = 1 (ja) & Indikation = 1 | 2 | 4

Mein Arzt / Psychotherapeut beschrieb mir ausführlich den Inhalt und Ablauf der Online-Selbsthilfe.

PROG: Frage 507 = 1 (ja) & Indikation = 3 | 5 | 6 | 7

Mein Arzt beschrieb mir ausführlich den Inhalt und Ablauf der Online-Selbsthilfe.

stimmt überhaupt nicht	stimmt nicht	weder noch	stimmt	stimmt voll und ganz
1	2	3	4	5

507_02 PROG: Frage 507 = 1 (ja) & Indikation = 1 | 2 | 4

Mein Arzt / Psychotherapeut erläuterte mir genau, warum die Online-Selbsthilfe für mich hilfreich ist.

PROG: Frage 507 = 1 (ja) & Indikation = 3 | 5 | 6 | 7

Mein Arzt erläuterte mir genau, warum die Online-Selbsthilfe für mich hilfreich ist.

stimmt überhaupt nicht	stimmt nicht	weder noch	stimmt	stimmt voll und ganz
1	2	3	4	5

507_1 PROG: Frage 507 = 1 (ja)

Hatten Sie dieses Angebot der Online-Selbsthilfe wahrgenommen?

❑ ja (1) ❑ nein (0)

507_5
507_6
507_7
507_8
507_8o

PROG: Frage 507 = 1 (ja) & Frage 507_1 = 0 (nein)

Sie haben angegeben, dass Sie an einer Online-Selbsthilfe, die Ihnen empfohlen wurde, nicht teilgenommen hatten.

Welche Gründe hatten Sie dafür?

PROG: Mehrfachantwort möglich

❑ Ich hatte kein Interesse daran. (1 genannt; 0 nicht genannt)

❑ Der Aufwand war mir zu groß. (1 genannt; 0 nicht genannt)

❑ Ich glaubte nicht, dass mir die Online-Selbsthilfe hilft. (1 genannt; 0 nicht genannt)

❑ anderer Grund (1 genannt; 0 nicht genannt)

PROG: Frage 507_8 = 1 (anderer Grund)

Und zwar: _____________

PROG: Frage 507 = 1 (ja) & Frage 507_1 = 1 (ja)

Sie nahmen bereits an einer Online-Selbsthilfe teil. Bitte geben Sie an, inwiefern Sie den folgenden Aussagen zustimmen.

507_9 PROG: Frage 507 = 1 (ja) & Frage 507_1 = 0 (nein)

Die Online-Selbsthilfe half mir sehr, im Alltag mit meiner Erkrankung zurecht zu kommen.

stimmt überhaupt nicht	stimmt nicht	weder noch	stimmt	stimmt voll und ganz
1	2	3	4	5

507_010 PROG: Frage 507 = 1 (ja) & Frage 507_1 = 0 (nein)

Durch die Online-Selbsthilfe belastete mich meine Erkrankung deutlich weniger.

stimmt überhaupt nicht	stimmt nicht	weder noch	stimmt	stimmt voll und ganz
1	2	3	4	5

507_011 PROG: Frage 507 = 1 (ja) & Frage 507_1 = 0 (nein)

Durch die Online-Selbsthilfe verstand ich meine Erkrankung besser.

stimmt überhaupt nicht	stimmt nicht	weder noch	stimmt	stimmt voll und ganz
1	2	3	4	5

511 PROG: Indikation = 1 | 2 | 4

Haben Sie in den letzten 12 Monaten Ihrer Behandlung Ihren Arzt / Psychotherapeuten, zu dem Sie vorwiegend gehen, gewechselt?

PROG: Indikation = 3 | 5 | 6 | 7

Haben Sie in den letzten 12 Monaten Ihrer Behandlung Ihren Arzt, zu dem Sie vorwiegend gehen, gewechselt?

❑ ja (1) ❑ nein (0)

511_1 PROG: Frage 511 = 1 (ja)

nämlich _____________ Mal (numerisch, dreistellig, > 0)

Schübe
Quelle: eigen
PROG: Indikation =5

516 PROG: Indikation = 5

Wann haben Sie Ihren letzten Schub (d.h. eine Verschlechterung Ihrer Erkrankung) erlebt?

habe keinen Schub erlebt	vor 1-6 Monaten	vor 7-12 Monaten	vor 12 bis 24 Monaten	vor mehr als 24 Monaten
1	2	3	4	5

516_1 PROG: Indikation = 5 & Frage 516 > 1 (Schub erlebt)

Haben Sie in dieser Zeit eine Schubtherapie gemacht?

❑ ja, ambulant bei meinem behandelnden Arzt (1)

❑ ja, in einem Krankenhaus (2)

❑ nein (0)

Entlassungsmanagement
Quelle: eigen

512 PROG: Indikation = 1 | 2 | 4

Wie viele Nächte waren Sie in den vergangenen 12 Monaten aufgrund Ihrer seelischen Erkrankung zur stationären Behandlung in einem Krankenhaus?

PROG: Indikation = 3 | 5 | 6 | 7

Wie viele Nächte waren Sie in den vergangenen 12 Monaten aufgrund Ihrer Erkrankung des Nervensystems zur stationären Behandlung in einem Krankenhaus?

____ Nächte (numerisch, 3-stellig, max: 366)

512_1 PROG: Frage 512 > 0 & Indikation = alle

Versuchen Sie sich an die letzte Entlassung aus dem Krankenhaus zu erinnern.

PROG: Indikation = 1 | 2 | 4

Wie viel Zeit ist damals zwischen Ihrer Entlassung und Ihrem ersten Termin bei Ihrem niedergelassenen Arzt / Psychotherapeuten verstrichen?

PROG: Indikation = 3 | 5 | 6 | 7

Wie viel Zeit ist damals zwischen Ihrer Entlassung und Ihrem ersten Termin bei Ihrem niedergelassenen Arzt verstrichen?

bis zu 7 Tage	1 bis 2 Wochen	3 bis 4 Wochen	1 bis 3 Monate	länger als 3 Monate
1	2	3	4	5

Behandlungsabbrüche

Quelle: eigen

PROG: teilweise nach Indikation gefiltert

PROG: kein Filter

Manchmal hat man das Gefühl, dass eine Behandlung nicht so gut hilft oder einem beispielsweise aufgrund von Nebenwirkungen nicht gut tut. Manche Patienten brechen deshalb eine Therapie gegen den ärztlichen Rat ab. Zum Beispiel gehen Sie zu vorgesehenen Folgeterminen nicht mehr hin oder nehmen die Medikamente nicht mehr wie vereinbart.

513 PROG: Indikation = 1 | 2 | 4

Haben Sie in den letzten 12 Monaten eine Behandlung Ihrer seelischen Erkrankung gegen ärztlichen Rat abgebrochen?

PROG: Indikation = 3 | 5 | 6 | 7

Haben Sie in den letzten 12 Monaten eine Behandlung Ihrer Erkrankung des Nervensystems gegen ärztlichen Rat abgebrochen?

❑... ja (1) ❑... nein (0)

Fragen zu Krisensituationen

Quelle: eigen

PROG: teilweise nach Indikation gefiltert

PROG: Indikation = 1 | 2 | 4

Manchmal gibt es bei einer Erkrankung Zeiten, in denen es einem plötzlich schlechter geht und man schnell einen Termin beim Arzt / Psychotherapeuten braucht.

PROG: Indikation = 3 | 5 | 6 | 7

Manchmal gibt es bei einer Erkrankung Zeiten, in denen es einem plötzlich schlechter geht und man schnell einen Termin beim Arzt braucht.

PROG: kein Filter

Denken Sie bitte für die folgenden Fragen an die letzten12 Monate zurück.

515 PROG: Indikation = 1 | 2 | 4

Gab es für Sie in den letzten 12 Monaten einen Zeitpunkt, an dem Sie aufgrund der Verschlechterung Ihrer seelischen Erkrankung schnell einen Behandlungstermin benötigten?

PROG: Indikation = 3 | 5 | 6 | 7

Gab es für Sie in den letzten 12 Monaten einen Zeitpunkt, an dem Sie aufgrund der Verschlechterung Ihrer Erkrankung des Nervensystems schnell einen Behandlungstermin benötigten?

❑... ja (1) ❑... nein (0)

515_1 PROG: Frage 515 = 1 (ja), Indikation = 1 | 2 | 4

Wie lange mussten Sie auf diesen Behandlungstermin bei Ihrem niedergelassenen Arzt / Psychotherapeuten warten?

PROG: Frage 515 = 1 (ja), Indikation = 3 | 5 | 6 | 7

Wie lange mussten Sie auf diesen Behandlungstermin bei Ihrem niedergelassenen Arzt warten?

Bis zu einem Tag	2-3 Tage	4-7 Tage	7-14 Tage	länger als 14 Tage
1	2	3	4	5

515_3 PROG: Frage 515 = 1 (ja)

Haben Sie aufgrund von zu langen Wartezeiten auf einen Termin eine Notaufnahme aufgesucht?

❑... ja (1) ❑... nein (0)

7. Inanspruchnahme und Bewertung projektspezifische Leistungen

Seitenüberschrift: Versorgung in NPPV

Quelle: eigen

PROG: Alle Fragen nur für IG
Gruppe = 1 (IG)

PROG: Extra Seite mit folgendem Text, nicht fett und größere Schrift

PROG: Gruppe = 1 & Indikation = 1 | 2 | 4
Seit kurzem nehmen Sie aufgrund Ihrer seelischen Erkrankung an dem neuen Versorgungsmodell (NPPV) teil.

Auf den folgenden Seiten möchten wir gern mehr darüber erfahren, wie Sie die Behandlung in dieser neuen Versorgungsform erleben und wie Sie die Qualität der Behandlung einschätzen.
Bitte denken Sie daran, falls Sie den Fragebogen stellvertretend für jemanden ausfüllen, alle Fragen aus Sicht der erkrankten Person zu beantworten.

PROG: Gruppe = 1 & Indikation = 3 | 5 | 6 | 7
Seit kurzem nehmen Sie aufgrund Ihrer Erkrankung des Nervensystems an dem neuen Versorgungsmodell (NPPV) teil.

Auf den folgenden Seiten möchten wir gern mehr darüber erfahren, wie Sie die Behandlung in dieser neuen Versorgungsform erleben und wie Sie die Qualität der Behandlung einschätzen.
Bitte denken Sie daran, falls Sie den Fragebogen stellvertretend für jemanden ausfüllen, alle Fragen aus Sicht der erkrankten Person zu beantworten.

PROG: Gruppe = 1 (für alle 600er-Fragen)

PROG: Indikation = 1 | 2 | 4 & Text immer bis Frage 600_2b einblenden

Sie nehmen seit kurzem aufgrund Ihrer seelischen Erkrankung an dem neuen Versorgungsmodell (NPPV) teil. Die folgenden Fragen beziehen sich auf Ihre Erfahrungen in den letzten Wochen mit dieser Behandlungsform.

PROG: Indikation = 3 | 5 | 6 | 7 & Text immer bis Frage 600_2b einblenden

Sie nehmen seit kurzem aufgrund Ihrer Erkrankung des Nervensystems an dem neuen Versorgungsmodell (NPPV) teil. Die folgenden Fragen beziehen sich auf Ihre Erfahrungen in den letzten Wochen mit dieser Behandlungsform.

600 Wie sind Sie auf das neue Versorgungsmodell (NPPV) aufmerksam gemacht worden?

❑ durch meinen Hausarzt (1)

❑ durch meinen Betriebsarzt (2)

❑ durch meinen Psychiater (3)

❑ durch meinen Neurologen oder Nervenarzt (4)

❑ durch meinen Psychotherapeuten (5)

❑ durch Ärzte / Therapeuten in einem Krankenhaus (6)

❑ durch meine Krankenkasse (7)

❑ durch jemand anderen (8)

nämlich _____________

600_1 PROG: Indikation = 1 | 2 | 4

Haben Sie, um an dem neuen Versorgungsmodell (NPPV) teilzunehmen, Ihren Arzt / Psychotherapeuten gewechselt oder einen neuen Arzt / Psychotherapeuten aufgesucht?

PROG: Indikation = 3 | 5 | 6 | 7

Haben Sie, um an dem neuen Versorgungsmodell (NPPV) teilzunehmen, Ihren Arzt gewechselt oder einen neuen Arzt aufgesucht??

ja (1) / nein (0)

600_2a PROG: Indikation = 1 | 2 | 4

Bei welchem Arzt / Therapeuten sind Sie im Rahmen des neuen Versorgungsmodells (NPPV) mit Ihrer seelischen Erkrankung überwiegend in Behandlung?

❑ Psychiater (1)

❑ Neurologe oder Nervenarzt (2)

❑ Psychotherapeut (3)

❑ anderer Arzt (4)

nämlich: __________________

600_2b PROG: Indikation = 3 | 5 | 6 | 7

Bei welchem Arzt sind Sie im Rahmen des neuen Versorgungsmodells (NPPV) mit Ihrer Erkrankung des Nervensystems in Behandlung?

❑ Neurologe oder Nervenarzt (1)

❑ anderer Arzt (2)

nämlich: __________________

Teil des neuen Versorgungsmodells in NPPV sind verschiedene Angebote und die Möglichkeit an Gruppen oder Online-Selbsthilfe (Novego) zur Unterstützung der Behandlung teilzunehmen. Wir möchten nun von Ihnen wissen, wie Sie die Behandlung im neuen Versorgungsmodell bewerten und welche Angebote Sie wahrnehmen.

600_3 PROG: Indikation = 1 | 2 | 4

In meiner Behandlung in NPPV hat mir mein Arzt / Psychotherapeut hilfreiche therapeutische Angebote empfohlen.

PROG: Indikation = 3 | 5 | 6 | 7

In meiner Behandlung in NPPV hat mir mein Arzt hilfreiche therapeutische Angebote empfohlen.

stimmt überhaupt nicht	stimmt nicht	weder noch	stimmt	stimmt voll und ganz
1	2	3	4	5

600_4 PROG: Indikation = 1 | 2 | 4

In meiner Behandlung in NPPV hat mir mein Arzt / Psychotherapeut ausführlich erklärt, was ich bei Notfällen und Krisen, zum Beispiel wenn es mir plötzlich schlechter geht, tun soll.

PROG: Indikation = 3 | 5 | 6 | 7

In meiner Behandlung in NPPV hat mir mein Arzt ausführlich erklärt, was ich bei Notfällen und Krisen, zum Beispiel wenn es mir plötzlich schlechter geht, tun soll.

stimmt überhaupt nicht	stimmt nicht	weder noch	stimmt	stimmt voll und ganz
1	2	3	4	5

600_5 PROG: Indikation = 1 | 2 | 4

In meiner Behandlung in NPPV hat mich mein Arzt / Psychotherapeut ausführlich informiert, dass ich in Notfällen und Krisen schnell einen Termin bei ihm bekomme.

PROG: Indikation = 3 | 5 | 6 | 7

In meiner Behandlung in NPPV hat mich mein Arzt ausführlich informiert, dass ich in Notfällen und Krisen schnell einen Termin bei ihm bekomme.

stimmt überhaupt nicht	stimmt nicht	weder noch	stimmt	stimmt voll und ganz
1	2	3	4	5

Gruppenangebote

601 PROG: Indikation = 1 | 2 | 4

Wurde Ihnen von Ihrem Arzt / Psychotherapeuten ein Gruppenangebot empfohlen?

PROG: Indikation = 3 | 5 | 6 | 7

Wurde Ihnen von Ihrem Arzt ein Gruppenangebot empfohlen?

Ja (1) / nein (0)

Wie wurden Sie über die Gruppenangebote informiert?

601_1 PROG: Frage 601 = 1 (ja) & Indikation = 1 | 2 | 4

Mein Arzt / Psychotherapeut hat mir ausführlich den Inhalt und Ablauf des Gruppenangebots beschrieben.

PROG: Frage 601 = 1 (ja) & Indikation = 3 | 5 | 6 | 7

Mein Arzt hat mir ausführlich den Inhalt und Ablauf des Gruppenangebots beschrieben.

stimmt überhaupt nicht	stimmt nicht	weder noch	stimmt	stimmt voll und ganz
1	2	3	4	5

601_2 PROG: Frage 601 = 1 (ja) & Indikation = 1 | 2 | 4

Mein Arzt / Psychotherapeut hat mir genau erläutert, warum das Gruppenangebot für mich hilfreich ist.

PROG: Frage 601 = 1 (ja) & Indikation = 3 | 5 | 6 | 7

Mein Arzt hat mir genau erläutert, warum das Gruppenangebot für mich hilfreich ist.

stimmt überhaupt nicht	stimmt nicht	weder noch	stimmt	stimmt voll und ganz
1	2	3	4	5

PROG: Frage 601 = 1 (ja)

Welche Gruppenangebote wurden Ihnen empfohlen und haben Sie diese bereits wahrgenommen?

PROG: Mehrfachantwort möglich

	Gruppenangebot	empfohlen	wahrgenommen
**601_31**<br>**601_32**	Gruppe für Betroffene	ja (1) / nein (0)	ja (1) / nein (0)
**601_41**<br>**601_42**	Gruppe für Angehörige	ja (1) / nein (0)	ja (1) / nein (0)

601_10 PROG: Frage 601 = 1 (ja) & mind. einmal (Frage 601_x1 = 1 (ja) & Frage 601_x2 = 0 (nein))

Sie haben angegeben, dass Sie mindestens an einem Angebot, das Ihnen empfohlen wurde, noch nicht teilgenommen haben. Haben Sie noch vor das Angebot wahrzunehmen?

ja (1) / nein (0)

PROG: PROG: Frage 601 = 1 (ja) & Frage 601_10 = 0 (nein)

Welche Gründe haben Sie dafür?

PROG: Mehrfachantwort möglich

❑ Ich habe kein Interesse daran. (1 genannt; 0 nicht genannt)

❑ Der Aufwand ist mir zu groß. (1 genannt; 0 nicht genannt)

❑ Der Weg ist mir zu weit. (1 genannt; 0 nicht genannt)

❑ Ich glaube nicht, dass mir das Angebot hilft. (1 genannt; 0 nicht genannt)

❑ Ich möchte meine Probleme nicht vor anderen Patienten besprechen. (1 genannt; 0 nicht genannt)

❑ anderer Grund (1 genannt; 0 nicht genannt)

PROG: Frage 601_13 = 1 (anderer Grund)

Und zwar: ____________

Sie haben an mindestens einem Gruppenangebot teilgenommen. Bitte geben Sie an, inwiefern Sie den Aussagen zustimmen.

601_5 PROG: Frage 601 = 1 (ja) & mind. einmal (Frage 601_x2 = 1 (ja))

Das Gruppenangebot hat mir sehr geholfen, im Alltag mit einer Erkrankung zurecht zu kommen.

stimmt überhaupt nicht	stimmt nicht	weder noch	stimmt	stimmt voll und ganz
1	2	3	4	5

601_6 PROG: Frage 601 = 1 (ja) & mind. einmal (Frage 601_x2 = 1 (ja))

Durch das Gruppenangebot belastet mich meine Erkrankung deutlich weniger.

stimmt überhaupt nicht	stimmt nicht	weder noch	stimmt	stimmt voll und ganz
1	2	3	4	5

601_7 PROG: Frage 601 = 1 (ja) & mind. einmal (Frage 601_x2 = 1 (ja))

Durch das Gruppenangebot habe ich meine Erkrankung besser verstanden.

stimmt überhaupt nicht	stimmt nicht	weder noch	stimmt	stimmt voll und ganz
1	2	3	4	5

601_8 PROG: Frage 601 = 1 (ja) & mind. einmal (Frage 601_x2 = 1 (ja))

Der Austausch mit anderen Betroffenen beim Gruppenangebot hat mir sehr geholfen.

stimmt überhaupt nicht	stimmt nicht	weder noch	stimmt	stimmt voll und ganz
1	2	3	4	5

Online-Selbsthilfe

602 PROG: Indikation = 1 | 2 | 4

Wurde Ihnen von Ihrem Arzt / Psychotherapeuten eine Online-Selbsthilfe (Novego) empfohlen?

PROG: Indikation = 3 | 5 | 6 | 7

Wurde Ihnen von Ihrem Arzt eine Online-Selbsthilfe (Novego) empfohlen?

Ja (1) / nein (0)

Wie wurden Sie über die Online-Selbsthilfe informiert?

602_1 PROG: Frage 602 = 1 (ja) & Indikation = 1 | 2 | 4

Mein Arzt / Psychotherapeut hat mir ausführlich den Inhalt und Ablauf der Online-Selbsthilfe (Novego) beschrieben.

PROG: Frage 602 = 1 (ja) & Indikation = 3 | 5 | 6 | 7

Mein Arzt hat mir ausführlich den Inhalt und Ablauf des Online-Selbsthilfe (Novego) beschrieben.

stimmt überhaupt nicht	stimmt nicht	weder noch	stimmt	stimmt voll und ganz
1	2	3	4	5

602_2 PROG: Frage 602 = 1 (ja) & Indikation = 1 | 2 | 4

Mein Arzt / Psychotherapeut hat mir genau erläutert, warum die Online-Selbsthilfe (Novego) für mich hilfreich ist.

PROG: Frage 602 = 1 (ja) & Indikation = 3 | 5 | 6 | 7

Mein Arzt hat mir genau erläutert, warum der Online-Selbsthilfe (Novego) für mich hilfreich ist.

stimmt überhaupt nicht	stimmt nicht	weder noch	stimmt	stimmt voll und ganz
1	2	3	4	5

602_3 PROG: Frage 602 = 1 (ja)

Haben Sie das Angebot der Online-Selbsthilfe (Novego) bereits wahrgenommen?

❑ ja (1)

❑ nein, aber ich habe vor es wahrzunehmen (2)

❑ nein und ich habe nicht vor es wahrzunehmen (0)

602_3 1 PROG: Frage 602 = 1 (ja) & Frage 602_3 = 0 (nein)

Sie haben angegeben, dass Sie an der Online-Selbsthilfe (Novego), die Ihnen empfohlen wurde, nicht teilnehmen wollen.

Welche Gründe haben Sie dafür?

PROG: Mehrfachantwort möglich

❑ Ich habe kein Interesse daran. (1 genannt; 0 nicht genannt)

❑ Der Aufwand ist mir zu groß. (1 genannt; 0 nicht genannt)

❑ Ich glaube nicht, dass mir die Online-Selbsthilfe (Novego) hilft. (1 genannt; 0 nicht genannt)

❑ anderer Grund (1 genannt; 0 nicht genannt)

Und zwar: _____________

PROG: Frage 602 = 1 (ja) & Frage 602_3 = 1 (ja)

Sie haben bereits an einer Online-Selbsthilfe (Novego) teilgenommen. Bitte geben Sie an, inwiefern Sie den folgenden Aussagen zustimmen.

602_4 PROG: Frage 602 = 1 (ja) & Frage 602_3 = 1 (ja)

Die Online-Selbsthilfe half mir sehr, im Alltag mit meiner Erkrankung zurecht zu kommen.

stimmt überhaupt nicht	stimmt nicht	weder noch	stimmt	stimmt voll und ganz
1	2	3	4	5

602_5 PROG: Frage 602 = 1 (ja) & Frage 602_3 = 1 (ja)

Durch die Online-Selbsthilfe belastete mich meine Erkrankung deutlich weniger.

stimmt überhaupt nicht	stimmt nicht	weder noch	stimmt	stimmt voll und ganz
1	2	3	4	5

602_6 PROG: Frage 602 = 1 (ja) & Frage 602_3 = 1 (ja)

Durch die Online-Selbsthilfe verstand ich meine Erkrankung besser.

stimmt überhaupt nicht	stimmt nicht	weder noch	stimmt	stimmt voll und ganz
1	2	3	4	5

8. Soziodemographie II

Seitenüberschrift: Angaben zur Person

PROG: Extra Seite mit folgendem Text, nicht fett und größere Schrift

Zum Abschluss der Befragung möchten wir Sie noch um ein paar Angaben zu Ihrer Person bitten.

PROG: kein Filter

Bitte denken Sie daran, falls Sie den Fragebogen stellvertretend für jemanden ausfüllen, alle Fragen aus Sicht der erkrankten Person zu beantworten.

Welche ist Ihre Muttersprache / sind Ihre Muttersprachen?

PROG: Mehrfachnennungen möglich

704_1 ❑ Deutsch (1 genannt; 0 nicht genannt)
704_2 ❑ Türkisch (1 genannt; 0 nicht genannt)
❑ Polnisch (1 genannt; 0 nicht genannt)

704_3 ❑ Russisch (1 genannt; 0 nicht genannt)
704_4 ❑ Italienisch (1 genannt; 0 nicht genannt)
❑ Englisch (1 genannt; 0 nicht genannt)

704_5 ❑ eine andere Sprache (1 genannt; 0 nicht genannt)

704_6 PROG: Frage 704_7 = 1 (genannt)

704_7 und zwar: ________________

704_7o

705 PROG: kein Filter

Wie viele Personen (inkl. Kinder) leben, wohnen und wirtschaften gemeinsam in Ihrem Haushalt?

________ Person(en) (PROG: >/= 1)

706 Welchen höchsten allgemeinbildenden Schulabschluss haben Sie?

❑ Hilfs- oder Sonderschule (1)
❑ Haupt- oder Volksschulabschluss (2)
❑ Realschulabschluss / Mittlere Reife / Fachschulreife (3)
❑ POS (Polytechn. Oberschule) bzw. 10. Klasse (vor 1965: 8. Klasse) (4)
❑ Fachhochschulreife / Abschluss einer Fachoberschule (5)
❑ Abitur, allgemeine oder fachgebundene Hochschulreife (6)
❑ Anderen Schulabschluss (z. B. im Ausland erworben) (7)
❑ Schule beendet ohne Schulabschluss (8)
❑ noch keinen Schulabschluss (9)

707 Machen Sie zurzeit eine Schulausbildung?

❑ nein (0)

❑ ja (1)

PROG: Frage 707 = 1 (ja)

und ich strebe folgenden Schulabschluss an:

707_1 ❑ Hauptschulabschluss (1) ❑ Realschulabschluss / Mittlere Reife/ Fachschulreife (2) ❑ Fachhochschulreife / Abschluss einer Fachoberschule (3)

❑ Abitur, allgemeine oder fachgebundene Hochschulreife (4) ❑ Anderen Schulabschluss (5)

Welchen höchsten Ausbildungsabschluss haben Sie?

708 ❑ Teilfacharbeiter (1) ❑ beruflich-betriebliche Berufsausbildung (Lehre) (2) ❑ beruflich-schulische Ausbildung (Berufsfach- oder Handelsschule) (3)

❑ Fachschule, Meister- oder Technikerschule, Berufs- oder Fachakademie (4) ❑ Fachhochschulabschluss (5) ❑ Hochschulabschluss (6)

❑ kein Ausbildungsabschluss (7) ❑ anderer Abschluss (8)

708_o PROG: Frage 709 = 8 (anderer Abschluss)

Und zwar: ____________________

PROG: kein Filter

Wie lässt sich Ihr derzeitiges Beschäftigungsverhältnis am ehesten beschreiben?

709 ❑ vollzeit erwerbstätig (1) ❑ teilzeit erwerbstätig (2) ❑ geringfügig oder unregelmäßig erwerbstätig (3)

❑ Rentner / Pensionär (4) ❑ arbeitslos (5) ❑ Hausfrau / Hausmann (6)

❑ vorrübergehend freigestellt / beurlaubt (z. B. Elternzeit) (7) ❑ aus anderen Gründen nicht erwerbstätig (8)

709_o PROG: Frage 709 = 1-3

Wie viele Stunden arbeiten Sie regulär pro Woche? _________ Stunden

710 PROG: kein Filter

Wie haben Sie den Fragebogen ausgefüllt?

❑ vorwiegend alleine (1)

❑ zusammen mit einer anderen Person (2)

❑ vorwiegend eine andere Person (3)

711o Haben Sie noch weitere Anregungen für uns?

9. Abschluss

VIELEN DANK FÜR IHRE TEILNAHME!

Hilfsvariablen zur Programmierung

Indikation = 1-7
Kodierung: 1 = Depression, 2 = Schizophrenie, schizotype oder wahnhafte Störungen, bipolare Störungen, 3 = Demenz; 4 = komplexe Traumafolgestörungen, 5 = Multiple Sklerose, 6 = Parkinson, 7 = Schlaganfall

Gruppe = 1;2
Kodierung: 1 = IG; 2 = KG

Einschub in Text beruhend auf Ausfülldatum:
[Monate] – die letzten 3 vollen Kalendermonate (z. B. „Januar, Februar und März"; wenn Ausfülldatum der 16.4.)
[Monat bis Monat] die letzten 6 vollen Kalendermonate (z. B. „Januar bis Juni"; wenn Ausfülldatum 13.7.)

Missings zulassen und kodieren: NULL
Kodiert im Pseudonym wird Gruppe und Indikation: Liste mit Pseudonymen + Indikation + Gruppe + Kennwort / Token
Speichern der Eingaben bei Abbruch: ja
Möglichkeit der Unterbrechung der Befragung: ja

Startseite

PROG: Gruppe = 1 (www.nppv-iges.de)

Herzlich Willkommen

Schön, dass Sie an der Befragung zur Qualität Ihrer Behandlung teilnehmen möchten. Sie helfen uns damit die Versorgung von Betroffenen zu verbessern.
Die Befragung findet im Rahmen der Versorgung Ihrer Erkrankung im Projekt zur Verbesserung der neurologisch-psychiatrischen und psychotherapeutischen Versorgung statt. Insgesamt werden Sie im Verlauf eines Jahres für 4 Befragungen kontaktiert.

Informationen zum Datenschutz

Ihre Daten werden streng nach den gesetzlichen Datenschutzbestimmungen verarbeitet. Es wird sichergestellt, dass die Daten der Befragung nicht mit Ihrer Person in Verbindung gebracht werden können. Zur Verknüpfung der vier Befragungszeitpunkte wird Ihnen ein Pseudonym zugewiesen. Nach Ende der letzten Befragung werden die Daten anonymisiert, so dass kein Personenbezug mehr herstellbar ist. Die Daten werden nur anonymisiert und in aggregierter Form veröffentlicht. Es finden keine Auswertungen für einzelne Personen statt. Nach Ablauf des Projektes werden die Daten unter Berücksichtigung der gesetzlichen Bestimmungen gelöscht.

Freiwilligkeit der Teilnahme

Ihre Teilnahme an der Befragung ist freiwillig und kann jederzeit ohne Angabe von Gründen beendet werden. Auch können Sie bis zum Zeitpunkt der Anonymisierung nach der letzten Befragung verlangen, dass alle von Ihnen erhobenen Daten gelöscht werden.
Weitere Informationen zur Teilnahme und zum Datenschutz finden Sie auch im Anschreiben Ihrer Krankenkasse.

Wenn Sie an der Befragung teilnehmen möchten und der beschriebenen Verarbeitung Ihrer Daten zustimmen, geben Sie bitte hier Ihr Passwort ein, das Sie von Ihrer Krankenkasse im Anschreiben erhalten haben:

PROG: Gruppe 2 (www.nppv-iges.de/KG)

Herzlich Willkommen

Schön, dass Sie an der Befragung zur Qualität Ihrer Behandlung teilnehmen möchten. Sie helfen uns damit die Versorgung von Betroffenen zu verbessern.
In dieser Befragung geht es um die Versorgung Ihrer seelischen und/oder neurologischen Erkrankung. Zurzeit werden Sie aufgrund dieser Erkrankung nach dem aktuellen Standard der Regelversorgung behandelt.

Um die Versorgung hierfür zu verbessern, fördert der Gesetzgeber neue Versorgungsprojekte im Rahmen des sogenannten *Innovationsfonds*. Eines dieser Projekte ist die Verbesserung der neurologischen-psychiatrischen und psychotherapeutischen Versorgung (NPPV).
Ziel von NPPV ist es, die Behandlung von psychischen und neurologischen Krankheiten zu verbessern. Dazu wird dem/r Patienten/in ein Bezugsarzt oder -therapeut bzw. eine Bezugsärztin oder -therapeutin an die Seite gestellt, der/die die verschiedenen Behandler/innen vernetzt und die Versorgung koordiniert.
Wir möchten herausfinden, ob NPPV die Versorgung spürbar verbessern kann und benötigen dazu Ihre Hilfe: Sie sind eingeladen an dieser Befragung teilzunehmen und durch Ihre Erfahrungen mit der bisherigen Regelversorgung einen wichtigen Beitrag zur Beurteilung der Versorgungsformen beizusteuern.
Die Ergebnisse dieser Befragungen können dazu führen, dass zukünftig alle gesetzlich Krankenversicherten von neuen Versorgungsformen profitieren.

Einverständniserklärung zur Datenverarbeitung
Für den Landesteil Nordrhein wird ein Projekt zur Verbesserung der neurologisch-psychiatrischen und psychotherapeutischen Versorgung (NPPV-Projekt) durchgeführt.
Ich nehme an dem NPPV-Projekt nicht teil. Ich bin aber durch das ausführliche Anschreiben meiner Krankenkasse eingehend darüber informiert worden, dass für alle gesetzlich krankenversicherten Patienten mit entsprechender Erkrankung ein Nutzen aus dem NPPV-Projekt nur festgestellt werden kann, wenn Daten von teilnehmenden Patienten mit Daten von nicht teilnehmenden Patienten (sog. Kontrollgruppe) verglichen werden.
Ich komme für eine Befragung in der sog. Kontrollgruppe in Betracht und stehe für diese zur Verfügung.
Ich habe darüber hinaus den Text der nachfolgenden Datenschutzerklärung gelesen und verstanden.
Datenschutzerklärung
Mir ist bekannt, dass personenbezogene Daten, d. h. Stammdaten (Name, Anschrift, Geburtsdatum etc.) sowie erforderliche Gesundheitsdaten (medizinische Behandlungsdaten) über mich bei meiner Krankenkasse erhoben, gespeichert und verarbeitet werden. Die Verwendung der Daten erfolgt nach gesetzlichen Bestimmungen. Ich erteile hiermit ausdrücklich die folgenden datenschutzrechtlichen Einwilligungserklärungen.
Ich erkläre mich damit einverstanden, dass meine Krankenkasse mir einen Fragebogen zur wissenschaftlichen Evaluation (Auswertung) zur Verfügung stellt, den ich online ausfülle und dessen Antworten pseudonymisiert (d. h. mit einer Kennung, aber ohne meinen Namen, Kontaktdaten oder sonstigen Angaben) bei der mit der wissenschaftlichen Evaluation beauftragten IGES Institut GmbH, Friedrichstraße 180, 10117 Berlin – vertretungsberechtigte Geschäftsführer: Prof. Dr. Bertram Häussler (Vorsitzender der Geschäftsführung), Dr. Martin Albrecht, Christoph Gipp, Hans-Dieter Nolting – gespeichert und verarbeitet werden. Die Befragung erfolgt ausschließlich bezogen auf eventuell bei mir vorliegende Krankheitsbilder, die auch Gegenstand des NPPV-Projekts sind; Rückschlüsse auf meine Person sind ausgeschlossen.

Ich bin darüber aufgeklärt worden, dass ich jederzeit meine Einwilligung zur Datenverarbeitung widerrufen kann und bis zum Zeitpunkt der Anonymisierung (nach Abschluss der letzten Befragung) verlangen kann, dass alle von mir erhobenen Daten gelöscht werden.

Für gesetzliche Vertreter/innen, Betreuer/innen bzw. Vorsorgebevollmächtigte:
Ich habe die obigen Ausführungen zur Einverständniserklärung zur Datenverarbeitung sowie die Datenschutzerklärung sorgfältig gelesen. Hiermit erkläre ich die Einwilligung des von mir betreuten bzw. vertretenen Patienten.

Wenn Sie an der Befragung teilnehmen möchten und der beschriebenen Verarbeitung Ihrer Daten zustimmen, geben Sie bitte hier Ihr Passwort ein, das Sie von Ihrer Krankenkasse im Anschreiben erhalten haben:

Übersicht über Ihre Befragungen

PROG: kein Filter

Insgesamt wird es vier Online-Befragungen geben, zu denen Sie jeweils von Ihrer Krankenkasse eingeladen werden. Die zweite Befragung findet ca. 3 Monate, die dritte ca. 6 Monate und die vierte ca. 12 Monate nach der ersten Befragung statt.
Bitte klicken Sie nachfolgend auf die nächst verfügbare Befragung, um mit dem Ausfüllen des Fragebogens zu beginnen.
PROG: Nur die aktuelle Befragung sollte auswählbar sein.
Befragung 1
Befragung 2
Befragung 3
Befragung 4

Einleitungstext

PROG: Gruppe = 1

	Lieber Teilnehmer, liebe Teilnehmerin,<br>Wir freuen uns sehr, dass Sie sich entschieden haben, an dem neuen Versorgungsmodell für Menschen mit psychischen und neurologischen Erkrankungen (NPPV) teilzunehmen. Um herauszufinden, wie gut Ihnen die neue Versorgungsform hilft und wie Sie diese bewerten, werden wir Ihnen nachfolgend Fragen zu Ihrem Gesundheitszustand und Ihren Erfahrungen mit der neuen Versorgungsform stellen. Einige der Fragen könnten Ihnen aus der letzten Befragung bekannt vorkommen. Wir würden uns sehr freuen, wenn Sie auch dieses Mal die Fragen wieder beantworten.<br><br>Das Ausfüllen wird etwa 30 Minuten in Anspruch nehmen. Sie können die Befragung jederzeit unterbrechen und zu einem späteren Zeitpunkt mit demselben Passwort fortsetzen. Sie können selbst entscheiden, ob Sie den Fragebogen alleine oder gemeinsam mit einer Bezugsperson ausfüllen. Die Bezugsperson kann die Beantwortung der Fragen auch vollständig übernehmen. Bitte geben Sie am Ende der Befragung an, wer den Fragebogen vorwiegend ausgefüllt hat.<br><br>Danke, dass Sie sich die Zeit nehmen die Fragen zu beantworten.<br><br>Bei Fragen zu der Befragung wenden Sie sich bitte an:<br>Tobias Woköck<br>Tel:<br>Email:

PROG: Gruppe = 2

	Lieber Teilnehmer, liebe Teilnehmerin,<br>Wir freuen uns sehr, dass Sie sich bereit erklären, diesen Fragebogen zu der Versorgung Ihrer Erkrankung auszufüllen. Sie helfen uns damit, die Versorgung ein Stück besser zu machen.<br>Um herauszufinden, wie gut Ihnen Ihre Versorgung hilft und wie Sie diese bewerten, werden wir Ihnen nachfolgend Fragen zu Ihrem Gesundheitszustand und Ihren Erfahrungen mit der Versorgung Ihrer Erkrankung stellen. Einige der Fragen könnten Ihnen aus der letzten Befragung bekannt vorkommen. Wir bitten Siewürden uns sehr freuen, wenn Sie auch dieses Mal die Fragen wieder den Instruktionen nach zu beantworten.<br><br>Das Ausfüllen wird etwa 30 Minuten in Anspruch nehmen. Sie können die Befragung jederzeit unterbrechen und zu einem späteren Zeitpunkt mit demselben Passwort fortsetzen. Sie können selbst entscheiden, ob Sie den Fragebogen alleine oder gemeinsam mit einer Bezugsperson ausfüllen. Die Bezugsperson kann die Beantwortung der Fragen auch vollständig übernehmen. Bitte geben Sie am Ende der Befragung an, wer den Fragebogen vorwiegend ausgefüllt hat.<br><br>Danke, dass Sie sich die Zeit nehmen die Fragen zu beantworten.

Bei Fragen zur Befragung wenden Sie sich bitte an:
Tobias Woköck
Tel:
Email:

PROG: Datum und Zeitstempel erfassen

1. Soziodemographie

**Seitenüberschrift:** Angaben zur Person

PROG: kein Filter

	**Zum Beginn möchten wir Sie um zwei kurze Angaben zu Ihrer Person bitten.**<br>**Falls Sie den Fragebogen stellvertretend für jemanden ausfüllen, beantworten Sie bitte alle Fragen aus Sicht der erkrankten Person.**
**001**	**Wie ist Ihr Geburtsjahr?**<br>_______ (numerisch, 4-stellig, min 1900)
**002**	**Welches ist Ihr Geschlecht?** ❑ männlich (1) ❑ weiblich (2)

2. Gesundheitsbezogene Lebensqualität (generisch)

**Seitenüberschrift:** Gesundheit & Lebensqualität

WHOQOL-BREF (26 Items):
Quelle: Hogrefe
Lizenz: gekauft

PROG: kein Filter

	**Die nun folgenden Fragen beschäftigen sich mit Ihrer Lebensqualität, Ihrer Gesundheit und anderen Bereichen Ihres Lebens. Wir möchten möglichst genau erfahren, wie es Ihnen im Allgemeinen und mit spezifischen Erkrankungen geht.**<br>**Bitte denken Sie daran, falls Sie den Fragebogen stellvertretend für jemanden ausfüllen, alle Fragen aus Sicht der erkrankten Person zu beantworten.**					
	**Bitte lesen Sie jede Frage, überlegen Sie, wie Sie sich in den letzten zwei Wochen gefühlt haben und geben Sie die Antwort an, die am ehesten auf Sie zutrifft.**					
		sehr schlecht	schlecht	mittelmäßig	gut	sehr gut
**101**	Wie würden Sie Ihre Lebensqualität beurteilen?	1	2	3	4	5
		sehr unzufrieden	unzufrieden	weder zufrieden noch unzufrieden	zufrieden	sehr zufrieden
**102**	Wie zufrieden sind Sie mit Ihrer Gesundheit?	1	2	3	4	5
	**In den folgenden Fragen geht es darum, wie stark Sie während der letzten zwei Wochen bestimmte Dinge erlebt haben.**					

		überhaupt nicht	ein wenig	mittelmäßig	ziemlich	äußerst
103	Wie stark werden Sie durch Schmerzen daran gehindert, notwendige Dinge zu tun?	1	2	3	4	5
104	Wie sehr sind Sie auf medizinische Behandlung angewiesen, um das tägliche Leben zu meistern?	1	2	3	4	5
105	Wie gut können Sie Ihr Leben genießen?	1	2	3	4	5
106	Betrachten Sie Ihr Leben als sinnvoll?	1	2	3	4	5
107	Wie gut können Sie sich konzentrieren?	1	2	3	4	5
108	Wie sicher fühlen Sie sich in Ihrem täglichen Leben?	1	2	3	4	5
109	Wie gesund sind die Umweltbedingungen in Ihrem Wohngebiet?	1	2	3	4	5
	**In den folgenden Fragen geht es darum, in welchem Umfang Sie <u>während der letzten zwei Wochen</u> bestimmte Dinge erlebt haben oder in der Lage waren, bestimmte Dinge zu tun.**					
		überhaupt nicht	eher nicht	halbwegs	überwiegend	völlig
110	Haben Sie genug Energie für das tägliche Leben?	1	2	3	4	5
111	Können Sie Ihr Aussehen akzeptieren?	1	2	3	4	5
112	Haben Sie genug Geld, um Ihre Bedürfnisse erfüllen zu können?	1	2	3	4	5
113	Haben Sie Zugang zu den Informationen, die Sie für das tägliche Leben brauchen?	1	2	3	4	5
114	Haben Sie ausreichend Möglichkeiten zu Freizeitaktivitäten?	1	2	3	4	5
		sehr schlecht	schlecht	mittelmäßig	gut	sehr gut
115	Wie gut können Sie sich fortbewegen?	1	2	3	4	5
	**In den folgenden Fragen geht es darum, wie zufrieden, glücklich oder gut Sie sich <u>während der letzten zwei Wochen</u> hinsichtlich verschiedener Aspekte Ihres Lebens gefühlt haben.**					

		sehr unzufrieden	unzufrieden	weder zufrieden noch unzufrieden	zufrieden	sehr zufrieden
**116**	Wie zufrieden sind Sie mit Ihrem Schlaf?	1	2	3	4	5
**117**	Wie zufrieden sind Sie mit Ihrer Fähigkeit, alltägliche Dinge erledigen zu können?	1	2	3	4	5
**118**	Wie zufrieden sind Sie mit Ihrer Arbeitsfähigkeit?	1	2	3	4	5
**119**	Wie zufrieden sind Sie mit sich selbst?	1	2	3	4	5
**120**	Wie zufrieden sind Sie mit Ihren persönlichen Beziehungen?	1	2	3	4	5
**121**	Wie zufrieden sind Sie mit Ihrem Sexualleben?	1	2	3	4	5
**122**	Wie zufrieden sind Sie mit der Unterstützung durch Ihre Freunde?	1	2	3	4	5
**123**	Wie zufrieden sind Sie mit Ihren Wohnbedingungen?	1	2	3	4	5
**124**	Wie zufrieden sind Sie mit Ihren Möglichkeiten, Gesundheitsdienste in Anspruch nehmen zu können?	1	2	3	4	5
**125**	Wie zufrieden sind Sie mit den Beförderungsmitteln, die Ihnen zur Verfügung stehen?	1	2	3	4	5
	**Bei der folgenden Frage geht es darum, wie oft sich während der letzten zwei Wochen bei Ihnen negative Gefühle eingestellt haben, wie zum Beispiel Angst oder Traurigkeit.**					
		niemals	nicht oft	zeitweilig	oftmals	immer
**126**	Wie häufig haben Sie negative Gefühle wie Traurigkeit, Verzweiflung, Angst oder Depression?	1	2	3	4	5
	PROG: Auf jeder Seite mit diesen Items<br>Hogrefe Copyright					

3. Gesundheitliche Situation (indikationsspezifisch)

**Seitenüberschrift:** Gesundheit & Lebensqualität

Fragen zur Erkrankung

Quelle: eigen

PROG:

Teilweise Filter nach Indikation und Gruppe

[Monate]: die letzten 3 vollen Kalendermonate einfügen

PROG: Extra Seite mit folgendem Text, nicht fett und größere Schrift

PROG: Gruppe = 1 & Indikation = 1 | 2 | 4
Sie nehmen aufgrund einer seelischen Erkrankung an dem neuen Versorgungsmodell (NPPV) teil. Wir möchten im Folgenden gern mehr über Ihre Erfahrungen mit dieser Erkrankung erfahren.

Es geht darum, wie Sie Ihre Krankheit erleben und wie es Ihnen mit Ihrer seelischen Erkrankung im Alltag geht. Uns ist dabei Ihre persönliche Einschätzung wichtig. Es gibt keine richtigen oder falschen Angaben.
Bitte denken Sie daran, falls Sie den Fragebogen stellvertretend für jemanden ausfüllen, alle Fragen aus Sicht der erkrankten Person zu beantworten.

PROG: Gruppe = 1 & Indikation = 3 | 5 | 6 | 7
Sie nehmen aufgrund einer Erkrankung des Nervensystems an dem neuen Versorgungsmodell (NPPV) teil. Wir möchten im Folgenden gern mehr über Ihre Erfahrungen mit dieser Erkrankung erfahren.

Es geht darum, wie Sie Ihre Krankheit erleben und wie es Ihnen mit Ihrer Erkrankung des Nervensystems im Alltag geht. Uns ist dabei Ihre persönliche Einschätzung wichtig. Es gibt keine richtigen oder falschen Angaben.
Bitte denken Sie daran, falls Sie den Fragebogen stellvertretend für jemanden ausfüllen, alle Fragen aus Sicht der erkrankten Person zu beantworten.

PROG: Gruppe = 2 & Indikation = 1 | 2 | 4
Wir haben Sie aufgrund einer seelischen Erkrankung zu dieser Befragung eingeladen. Wir möchten im Folgenden gern mehr über Ihre Erfahrungen mit dieser Erkrankung erfahren.

Es geht darum, wie Sie Ihre Krankheit erleben und wie es Ihnen mit Ihrer seelischen Erkrankung im Alltag geht. Uns ist dabei Ihre persönliche Einschätzung wichtig. Es gibt keine richtigen oder falschen Angaben.
Bitte denken Sie daran, falls Sie den Fragebogen stellvertretend für jemanden ausfüllen, alle Fragen aus Sicht der erkrankten Person zu beantworten.

PROG: Gruppe = 2 & Indikation = 3 | 5 | 6 | 7
Wir haben Sie aufgrund einer Erkrankung des Nervensystems zu dieser Befragung eingeladen. Wir möchten im Folgenden gern mehr über Ihre Erfahrungen mit dieser Erkrankung erfahren.

Es geht darum, wie Sie Ihre Krankheit erleben und wie es Ihnen mit Ihrer Erkrankung des Nervensystems im Alltag geht. Uns ist dabei Ihre persönliche Einschätzung wichtig. Es gibt keine richtigen oder falschen Angaben.
Bitte denken Sie daran, falls Sie den Fragebogen stellvertretend für jemanden ausfüllen, alle Fragen aus Sicht der erkrankten Person zu beantworten.

PROG: Gruppe 1 & Indikation = 1 | 2 | 4

Sie nehmen aufgrund einer seelischen Erkrankung an dem neuen Versorgungsmodell (NPPV) teil. Die folgenden Fragen beziehen sich daher auf Ihre Erfahrungen mit dieser Erkrankung.

PROG: Gruppe 1 & Indikation = 3 | 5 | 6 | 7

Sie nehmen aufgrund einer Erkrankung des Nervensystems an dem neuen Versorgungsmodell (NPPV) teil. Die folgenden Fragen beziehen sich daher auf Ihre Erfahrungen mit dieser Erkrankung.

PROG: Gruppe 2 & Indikation = 1 | 2 | 4

Wir haben Sie aufgrund einer seelischen Erkrankung zu dieser Befragung eingeladen. Die folgenden Fragen beziehen sich daher auf Ihre Erfahrungen mit dieser Erkrankung.

PROG: Gruppe 1 & Indikation = 3 | 5 | 6 | 7

Wir haben Sie aufgrund einer Erkrankung des Nervensystems zu dieser Befragung eingeladen. Die folgenden Fragen beziehen sich daher auf Ihre Erfahrungen mit dieser Erkrankung.

302 PROG: kein Filter

Inwiefern hatten Sie in den letzten 2 Wochen aufgrund dieser Erkrankung körperliche und/oder seelische Beschwerden?

keine	leichte	mittlere	große
1	2	3	4

303 **Wie sehr fühlten Sie sich in den letzten 2 Wochen durch diese Erkrankung in Ihrem Alltag belastet?**

überhaupt nicht	kaum	etwas	ziemlich	sehr
1	2	3	4	5

304 **Wir möchten gern von Ihnen wissen, wie sich Ihre Erkrankung in der letzten Zeit verändert hat. Die folgenden Fragen beziehen sich daher auf die letzten 3 Monate, das heißt auf** [PROG: Monate]**.**

Hat sich Ihre Erkrankung in den letzten 3 Monaten verbessert, verschlechtert oder ist sie gleich geblieben?

deutlich verschlechtert	verschlechtert	gleich geblieben	verbessert	deutlich verbessert
1	2	3	4	5

305 PROG: Indikation = 1 | 2 | 4

Nehmen Sie zurzeit Medikamente gegen die seelische Erkrankung ein?

PROG: Indikation = 3 | 5 | 6 | 7

Nehmen Sie zurzeit Medikamente gegen die Erkrankung des Nervensystems ein?

❑... ja (1) ❑... nein (0)

4. Lebensqualität (indikationsspezifisch)

Seitenüberschrift: Gesundheit & Lebensqualität

Q-LES-Q 18 (Depression; Schizophrenie, schizotype oder wahnhafte Störungen, bipolare Störungen; Traumafolgestörungen)

Quelle: Ritsner, M., Kurs, R., Gibel, A., Ratner, Y. & Endicott, J. (2005). Validity of an abbreviated Quality of Life Enjoyment and Satisfaction Questionnaire (Q-LES-Q-18) for schizophrenia, schizoaffective,and mood disorder patients. Quality of Life research, 14, 1693-1703.

Endicott J, Nee J, Harrison W, Blumenthal R. Quality of Life Enjoyment and Satisfaction Questionnaire: a new measure. Psychopharmacol Bull. 1993;29(2):321-6

Rohenkohl, A., Ruppelt, F., Gallinat, J., Karow, A., Lüdecke, D., Nawara, L. A., … Lambert, M. (2015). Erfassung der Lebensqualität bei PsychosepatientInnen – psychometrische Analyse des Q-LES-Q-18 Fragebogens

Lizenzfrei in Ritsner publiziert

Deutsche Übersetzung mit Rückübersetzung selbst gemacht

PROG: Indikation = 1 | 2 | 4 (Depression; Schizophrenie, schizotype oder wahnhafte Störungen, bipolare Störungen; Traumafolgestörungen)

PROG: Indikation = 1 | 2 | 4
In den folgenden Fragen geht es darum, wie es Ihnen <u>in den letzten zwei Wochen</u> in verschiedenen Bereichen Ihres Lebens ergangen ist.

	**Wie häufig…**	nie	selten	manch mal	häufig	immer
**1401**	… waren Sie bei sehr guter körperlicher Gesundheit?	1	2	3	4	5
**1402**	… waren Sie frei von Sorgen über Ihre körperliche Gesundheit?	1	2	3	4	5
**1403**	… fühlten Sie sich körperlich gut?	1	2	3	4	5
**1404**	… fühlten Sie sich voller Energie und Vitalität?	1	2	3	4	5
**1405**	… waren Sie zufrieden mit Ihrem Leben?	1	2	3	4	5
**1406**	… fühlten Sie sich glücklich oder fröhlich?	1	2	3	4	5
**1407**	… fühlten Sie sich in der Lage mit anderen zu kommunizieren?	1	2	3	4	5
**1408**	… fühlten Sie sich in der Lage sich zu Fuß, mit dem Auto, Bus, Bahn oder Fahrrad fortzubewegen, um Erledigungen zu machen?	1	2	3	4	5
**1409**	… fühlten Sie sich in der Lage, sich um sich selbst zu kümmern?	1	2	3	4	5
**1410**	… sind Sie Ihren Freizeitaktivitäten nachgegangen?	1	2	3	4	5

**1411**	… haben Sie sich auf die Freizeitaktivitäten konzentriert und ihnen Aufmerksamkeit geschenkt?	1	2	3	4	5
**1412**	Wenn bei Ihren Freizeitaktivitäten ein Problem auftauchte, wie oft konnten Sie es lösen oder damit ohne übermäßigen Stress umgehen?	1	2	3	4	5
**1413**	… haben Sie sich auf ein Zusammentreffen mit Freunden oder Verwandten gefreut?	1	2	3	4	5
**1414**	… hat es Ihnen Spaß gemacht, mit Kollegen oder Nachbarn zu sprechen?	1	2	3	4	5
**1415**	… haben Sie Zuneigung gegenüber einer oder mehrerer Personen gespürt?	1	2	3	4	5
**1416**	… haben Sie mit anderen Menschen gescherzt oder gelacht?	1	2	3	4	5
**1417**	… waren Sie für Ihre Freunde oder Verwandten da, wenn diese Sie gebraucht haben?	1	2	3	4	5

DEMQoL (Demenz)

Quelle: Smith, S. C., Lamping, D. L., Banerjee, S., Harwood, R., Foley, B., Smith, P., ... & Mann, A. (2005). Measurement of health-related quality of life for people with dementia: development of a new instrument (DEMQOL) and an evaluation of current methodology. Health Technology Assessment (Winchester, England), 9(10), 1-93.

Berwig, M., Leicht, H., & Gertz, H. J. (2009). Critical evaluation of self-rated quality of life in mild cognitive impairment and Alzheimer's disease — Further evidence for the impact of anosognosia and global cognitive impairment. JNHA - The Journal of Nutrition, Health and Aging, 13(3), 226–230. doi:10.1007/s12603-009-0063-4

Berwig, M., Leicht, H., Hartwig, K., & Gertz, H. J. (2011). Self-rated quality of life in mild cognitive impairment and Alzheimer's disease: The problem of affective distortion. GeroPsych: The Journal of Gerontopsychology and Geriatric Psychiatry, 24(1), 45–51. doi:http://dx.doi.org/10.1024/1662-9647/a000029

Lizenzfrei, deutsche Version von Brewig erhalten; Item nach genereller LQ gestrichen, da schon in WHOQoL

PROG: Indikation = 3 (Demenz)

PROG: Indikation = 3

Im Folgenden möchten wir Sie zu Beschwerden befragen, die häufig im Alter auftreten. Beispiele sind Gedächtnisstörungen, Schwierigkeiten im alltäglichen Leben oder eine gedrückte Stimmung. Wir möchten Sie auch fragen, wie häufig die Beschwerden waren und wie besorgt Sie über die Beschwerden waren. Es gibt keine richtigen und keine falschen Antworten.

	**Zunächst möchten wir Sie zu Ihren Gefühlen fragen. Wie oft haben Sie die folgenden Gefühle in den letzten 2 Wochen erlebt?**	nie	selten	manchmal	häufig
**3401**	Heiterkeit	1	2	3	4
**3402**	Angst oder Sorge	1	2	3	4
**3403**	Haben Sie das Leben genossen?	1	2	3	4
**3404**	Frustration	1	2	3	4
**3405**	Selbstsicherheit	1	2	3	4
**3406**	Voller Energie	1	2	3	4
**3407**	Traurigkeit	1	2	3	4
**3408**	Einsamkeit	1	2	3	4
**3409**	Verzweiflung	1	2	3	4
**3410**	Lebhaftigkeit	1	2	3	4
**3411**	Gereiztheit	1	2	3	4
**3412**	Überdruss	1	2	3	4

3413	Unfähigkeit	1	2	3	4
	**Jetzt folgen Fragen zu Ihrem Gedächtnis. Wie oft waren Sie in den letzten 2 Wochen besorgt wegen der folgenden Beschwerden?**	1	2	3	4
3414	Besorgt, weil Sie kurz zurückliegende Ereignisse vergessen	1	2	3	4
3415	Besorgt, weil Sie vergessen, wer bestimmte Personen sind	1	2	3	4
3416	Besorgt, weil Sie vergessen, welcher Wochentag ist	1	2	3	4
3417	Besorgt, weil Ihre Gedanken durcheinander sind	1	2	3	4
3418	Besorgt, weil Sie keine Entscheidungen treffen können	1	2	3	4
3419	Besorgt wegen Konzentrationsstörungen				
	**Jetzt folgen Fragen über Ihren Alltag. Wie oft waren Sie in den letzten 2 Wochen besorgt über folgende Lebensumstände?**	1	2	3	4
3420	Besorgt, weil Sie nicht genug Gesellschaft hatten	1	2	3	4
3421	Besorgt, weil Sie nicht wussten, wie Sie mit anderen Menschen in Ihrer Umgebung umgehen sollen	1	2	3	4
3422	Besorgt, weil Sie nicht die Zuneigung bekamen, die Sie sich gewünscht haben	1	2	3	4
3423	Besorgt, weil Ihnen nicht zugehört wurde	1	2	3	4
3424	Besorgt, weil Sie sich nicht verständlich machen konnten	1	2	3	4
3425	Besorgt, weil Sie nicht die Hilfe bekommen haben, die Sie gebraucht hätten	1	2	3	4
3426	Besorgt, es nicht rechtzeitig auf die Toilette zu schaffen	1	2	3	4
3427	Besorgt, weil Sie sich in ihrer eigenen Haut nicht wohl fühlten	1	2	3	4
3428	Besorgt über Ihren allgemeinen Gesundheitszustand	1	2	3	4

MSIS

Quelle: Hobart, J., Lamping, D., Fitzpatrick, R., Riazi, A., Thompson, A. (2001). The Multiple Sclerosis Impact Scale (MSIS-29): A new patient-based outcome measure. Brain, 124, 962-973.
Schönberg, P. (2012). Validierung der deutschen Version der Multiple Sclerosis Impact Scale (MSIS-29). Dissertation. Universitätsklinikum Hamburg-Eppendorf
lizenzfrei

PROG: Indikation = 5 (Multiple Sklerose)

PROG: Indikation = 5

In den folgenden Fragen geht es um den Einfluss Ihrer Multiple Sklerose Erkrankung auf Ihr tägliches Leben.

Bitte kreuzen Sie für jede Aussage an, was am besten auf Ihre Situation zutrifft.

	**Wie schwer fiel es Ihnen <u>in den letzten zwei Wochen</u> …**	gar nicht	ein bisschen	mäßig	ziemlich	sehr
**5401**	körperlich anstrengende Dinge zu tun?	1	2	3	4	5
**5402**	Dinge fest anzufassen (z. B. Hahn aufdrehen)?	1	2	3	4	5
**5403**	Dinge zu tragen?	1	2	3	4	5
	**Hatten Sie <u>in den letzten zwei Wochen</u>…**					
**5404**	Probleme mit dem Gleichgewicht?	1	2	3	4	5
**5405**	Schwierigkeiten, sich in der Wohnung zu bewegen?	1	2	3	4	5
**5406**	das Gefühl ungeschickt zu sein?	1	2	3	4	5
**5407**	ein Steifigkeitsgefühl?	1	2	3	4	5
**5408**	schwere Arme und / oder Beine?	1	2	3	4	5
**5409**	Zittern der Arme oder Beine?	1	2	3	4	5
**5410**	Krämpfe der Extremitäten?	1	2	3	4	5
**5411**	das Gefühl, dass ihr Körper nicht tat, was sie wollten?	1	2	3	4	5
**5412**	Beeinträchtigung im sozialen und Freizeitleben zu Hause?	1	2	3	4	5
**5413**	Probleme mit den Händen bei Alltagstätigkeiten?	1	2	3	4	5
**5414**	Probleme sich fortzubewegen (Auto, Bus, Taxi, Zug)?	1	2	3	4	5
**5415**	länger gebraucht, Dinge zu tun?	1	2	3	4	5
**5416**	Schwierigkeiten, Dinge spontan zu machen?	1	2	3	4	5

5417	das Gefühl, ganz schnell zur Toilette zu müssen?	1	2	3	4	5
5418	sich allgemein unwohl gefühlt?	1	2	3	4	5
5419	Schlafprobleme?	1	2	3	4	5
5420	sich geistig / mental müde gefühlt?	1	2	3	4	5
5421	Sorgen bezogen auf ihre MS?	1	2	3	4	5
5422	sich angespannt und ängstlich gefühlt?	1	2	3	4	5
5423	sich ungeduldig und aufbrausend gefühlt?	1	2	3	4	5
5424	Konzentrationsprobleme?	1	2	3	4	5
5425	keine Zuversicht?	1	2	3	4	5
5426	sich traurig / depressiv gefühlt?	1	2	3	4	5
	**Waren Sie <u>in den letzten zwei Wochen</u>…**					
5427	davon abhängig, dass andere Dinge für sie erledigten?	1	2	3	4	5
5428	gezwungen, zu Hause zu bleiben?	1	2	3	4	5
5429	Gezwungen, die Zeit für Arbeit oder Alltagsaktivitäten einzuschränken?	1	2	3	4	5

PROG: Auf jeder Seite mit diesen Items:

®2000 Neurological Outcome Measures Unit

PDQ-39 (Parkinson)

Quelle: Berger, K., Broll, S., Winkelmann, J., Heberlein, I., Müller, T., Ries, V. für die FAQT-Studienzentren (1999). Untersuchung zur Reliabilität der deutschen Version des PDQ-39: Ein krankheitsspezifischer Fragebogen zur Erfassung der Lebensqualität von Parkinson-Patienten. Aktuelle Neurologie, 26, 180-184.

lizenzfrei

PROG: Indikation = 6 (Parkinson)

PROG: Indikation = 6

In den folgenden Fragen geht es darum, wie es Ihnen in verschiedenen Bereichen in Ihrem Leben ergangen ist.

	**Wie oft haben Sie in den letzten 2 Wochen wegen Ihrer Parkinsonerkrankung…**	niemals	selten	manchmal	häufig	Immer oder kann ich überhaupt nicht
**6401**	… Schwierigkeiten gehabt, Freizeitaktivitäten, die Sie gern machen würden, auszuüben?	1	2	3	4	5
**6402**	… Schwierigkeiten gehabt, Ihren Haushalt zu versorgen (z. B. handwerkliche Tätigkeiten, Hausarbeiten, Kochen)?	1	2	3	4	5
**6403**	… Schwierigkeiten gehabt, Einkaufstaschen zu tragen?	1	2	3	4	5
**6404**	… Probleme gehabt, ungefähr 1 km zu gehen?	1	2	3	4	5
**6405**	… Probleme gehabt, ungefähr 100 m zu gehen?	1	2	3	4	5
**6406**	… Probleme gehabt, sich im Haus so zu bewegen, wie Sie wollten?	1	2	3	4	5
**6407**	… Probleme gehabt, sich in der Öffentlichkeit zu bewegen?	1	2	3	4	5
**6408**	… eine Begleitperson gebraucht, um sich außer Haus zu bewegen?	1	2	3	4	5
**6409**	… Angst oder Sorgen gehabt, dass Sie in der Öffentlichkeit hinfallen?	1	2	3	4	5
**6410**	… das Gefühl gehabt, mehr an das Haus gebunden zu sein, als Ihnen lieb wäre?	1	2	3	4	5
**6411**	… Schwierigkeiten gehabt, sich selbst zu waschen?	1	2	3	4	5
**6412**	… Schwierigkeiten gehabt, sich selbst anzuziehen?	1	2	3	4	5
**6413**	… Probleme gehabt, Knöpfe zu schließen oder Schnürsenkel zu binden?	1	2	3	4	5
**6414**	… Probleme gehabt, deutlich zu schreiben?	1	2	3	4	5
**6415**	… Schwierigkeiten gehabt, Ihr Essen klein zu schneiden?	1	2	3	4	5
**6416**	… Schwierigkeiten gehabt, ein Getränk zu halten, ohne es zu verschütten?	1	2	3	4	5

6417	… sich niedergeschlagen oder deprimiert gefühlt?	1	2	3	4	5
6418	… sich isoliert oder einsam gefühlt?	1	2	3	4	5
6419	… sich verärgert oder verbittert gefühlt?	1	2	3	4	5
6420	… sich den Tränen nahe gefühlt?	1	2	3	4	5
6421	… sich ängstlich gefühlt?	1	2	3	4	5
6422	… sich Sorgen über Ihre Zukunft gemacht?	1	2	3	4	5
6423	… das Gefühl gehabt, Ihre Parkinsonerkrankung vor anderen verheimlichen zu müssen?	1	2	3	4	5
6424	… Situationen vermieden, die mit Essen oder Trinken in der Öffentlichkeit verbunden waren?	1	2	3	4	5
6425	… sich in der Öffentlichkeit wegen Ihrer Parkinsonerkrankung geschämt?	1	2	3	4	5
6426	… sich Sorgen über die Reaktionen anderer Ihnen gegenüber gemacht?	1	2	3	4	5
6427	… Probleme im Verhältnis mit Ihnen nahe stehenden Menschen gehabt?	1	2	3	4	5
6428	… nicht die Unterstützung erhalten, die Sie von Ihrem (Ehe-)Partner benötigt hätten?	1	2	3	4	5
6429	… nicht die Unterstützung erhalten, die Sie von Ihren Verwandten oder engen Freunden benötigt hätten?	1	2	3	4	5
6430	… das Problem gehabt, tagsüber unerwartet einzuschlafen?	1	2	3	4	5
6431	… Probleme gehabt, sich zu konzentrieren (z. B. beim Lesen oder beim Fernsehen)?	1	2	3	4	5
6432	… das Gefühl gehabt, dass Sie ein schlechtes Gedächtnis hätten?	1	2	3	4	5
6433	… schlechte Träume oder Halluzinationen gehabt?	1	2	3	4	5
6434	… Schwierigkeiten mit dem Sprechen gehabt?	1	2	3	4	5
6435	… sich außer Stande gefühlt, mit anderen zu kommunizieren?	1	2	3	4	5
6436	… den Eindruck gehabt, von anderen nicht beachtet zu werden?	1	2	3	4	5
6437	… schmerzhafte Muskelkrämpfe gehabt?	1	2	3	4	5
6438	… Schmerzen in den Gelenken oder anderen Körperteilen gehabt?	1	2	3	4	5
6439	… sich unangenehm heiß oder kalt gefühlt?	1	2	3	4	5

SA-SIP30 (Schlaganfall)

Quelle: van Straten, A., de Haan, R. J., Limburg, M., Schuling, J., Bossuyt, P. M., van de Bos, G. A. M. (1997). A Stroke-Adapted 30-Item Version of the Sickness Impact Profile to Assess Quality of Life (SA-SIP30). Stroke, 28, 2155-2161.

Hütter, B. O. (2002). Sickness Impact Profile (SIP) -German version. In S. Salek (Ed.), Compendium of qua/ity of /ife instruments. Chichester, West Sussex: Wiley.

Lizenzfrei – deutsche Version von Hütter bekommen

PROG: Indikation = 7 (Schlaganfall)

PROG: Indikation = 7

Dieser Fragebogen dient dazu festzustellen, welche Beschwerden Sie im Augenblick haben.

Bitte lesen Sie sich alle Sätze genau durch und antworten Sie nur bei solchen Beschwerden mit "ja", die Sie am heutigen Tag haben und die sich auf Ihren Gesundheitszustand beziehen.

		ja	nein
**7401**	Ich kann schwierige Bewegungen nur mit Hilfe machen, wie z. B. in ein Auto oder eine Badewanne ein- und aussteigen.	1	0
**7402**	Meine Hände oder Finger kann ich nur mit Einschränkungen oder Schwierigkeiten gebrauchen.	1	0
**7403**	Wenn ich ins Bett gehen will oder aufstehen möchte, z. B. von einem Stuhl, so muss ich mich an etwas festhalten oder einen Stock benutzen.	1	0
**7404**	Ich habe Schwierigkeiten, mir alleine Schuhe, Strümpfe oder Socken anzuziehen.	1	0
**7405**	Ich kann mich nur anziehen, wenn mir jemand hilft.	1	0
**7406**	Ich kann mich für die Probleme von anderen nicht mehr so interessieren, z. B. höre ich nicht zu, wenn sie mir von ihren Problemen erzählen, oder ich biete ihnen keine Hilfe an.	1	0
**7407**	Ich bin oft ungehalten zu denjenigen, die mich umgeben, z. B. schneide ich ihnen das Wort ab, gebe scharfe Antworten oder kritisiere leicht.	1	0
**7408**	Ich zeige weniger Zuneigung.	1	0
**7409**	Ich unternehme weniger soziale Aktivitäten mit Gruppen von Leuten.	1	0
**7410**	Ich spreche weniger mit denjenigen, die mich umgeben.	1	0
**7411**	Ich bleibe die meiste Zeit zu Hause.	1	0
**7412**	Ich gehe nicht in die Stadt.	1	0
**7413**	Ich bewege mich ohne fremde Hilfe nicht in der Dunkelheit oder in unbeleuchteten Plätzen.	1	0

7414	Ich nehme nur noch dann an einem Gespräch teil, wenn ich der anderen Person sehr nahe stehe oder zu ihr schaue.	1	0
7415	Ich habe Schwierigkeiten in der Aussprache, z. B. stottere ich, bleibe stecken, stammle oder kann die Worte nicht mehr deutlich aussprechen.	1	0
7416	Ich kann nicht klar sprechen, wenn ich unter Stress bin.	1	0
7417	Ich sage, wie schlecht oder nutzlos ich bin, z. B. dass ich für andere eine Last bin.	1	0
7418	Ich lache oder weine plötzlich.	1	0
7419	Ich bin gereizt und ungeduldig mit mir selbst, z. B. spreche ich schlecht über mich, verfluche mich, gebe mir die Schuld, wenn etwas passiert.	1	0
7420	Ich bekomme plötzliche Angstzustände.	1	0
7421	Ich kann die normale Hausarbeit, die ich eigentlich tun müsste, überhaupt nicht mehr tun.	1	0
7422	Ich gehe nicht mehr einkaufen, so wie ich es normalerweise tun würde.	1	0
7423	Ich kann nicht mehr die Wohnung oder das Haus putzen, so wie ich es normalerweise tun würde.	1	0
7424	Ich kann die Wäsche nicht mehr waschen, so wie ich es normalerweise tun würde.	1	0
7425	Ich bin durcheinander und fange mehrere Sachen gleichzeitig an.	1	0
7426	Ich mache mehr Fehler als üblich.	1	0
7427	Ich habe Schwierigkeiten, Tätigkeiten auszuführen, die Konzentration und Denken erfordern.	1	0
7428	Ich gehe keine Steigungen mehr und / oder vermeide abschüssige Strecken.	1	0
7429	Ich kann mich nur noch mit Hilfe eines Spazierstocks, Krücken, Wänden oder Möbeln fortbewegen.	1	0
7430	Ich laufe langsamer.	1	0

5. IG: Versorgungsqualität & Inanspruchnahme und Bewertung projektspezifischer Leistungen

Seitenüberschrift: Versorgung in NPPV

Quelle: eigen

PROG: Alle Fragen unter 5. nur für IG
Gruppe = 1 (IG)

PROG: Extra Seite mit folgendem Text, nicht fett und größere Schrift

PROG: Gruppe = 1 & Indikation = 1 | 2 | 4
Sie nehmen seit einiger Zeit aufgrund Ihrer seelischen Erkrankung an dem neuen Versorgungsmodell (NPPV) teil. In Ihrer Behandlung wurde Ihnen ein Bezugstherapeut bzw. eine Bezugstherapeutin zur Seite gestellt. Die folgenden Fragen beziehen sich auf die Behandlung bei Ihrem Bezugstherapeuten oder Ihrer Bezugstherapeutin.

Auf den folgenden Seiten möchten wir gern mehr darüber erfahren, wie Sie die Behandlung in dieser neuen Versorgungsform erleben und wie Sie die Qualität der Behandlung einschätzen.
Bitte denken Sie daran, falls Sie den Fragebogen stellvertretend für jemanden ausfüllen, alle Fragen aus Sicht der erkrankten Person zu beantworten.

PROG: Gruppe = 1 & Indikation = 3 | 5 | 6 | 7
Sie nehmen seit einiger Zeit aufgrund Ihrer Erkrankung des Nervensystems an dem neuen Versorgungsmodell (NPPV) teil. In Ihrer Behandlung wurde Ihnen ein Bezugsarzt bzw. eine Bezugsärztin zur Seite gestellt. Die folgenden Fragen beziehen sich auf die Behandlung bei Ihrem Bezugsarzt oder Ihrer Bezugsärztin.

Auf den folgenden Seiten möchten wir gern mehr darüber erfahren, wie Sie die Behandlung in dieser neuen Versorgungsform erleben und wie Sie die Qualität der Behandlung einschätzen.
Bitte denken Sie daran, falls Sie den Fragebogen stellvertretend für jemanden ausfüllen, alle Fragen aus Sicht der erkrankten Person zu beantworten.

PROG: Gruppe = 1 (für alle Fragen unter 5.; bis Frage 515_3)

PROG: Indikation = 1 | 2 | 4 & Text immer bis Frage 509_1 einblenden

Sie nehmen seit einiger Zeit aufgrund Ihrer seelischen Erkrankung an dem neuen Versorgungsmodell (NPPV) teil. Die folgenden Fragen beziehen sich auf Ihre Erfahrungen in den letzten drei Monaten mit dieser Behandlungsform, das heißt die Monate [Monate].

PROG: Indikation = 3 | 5 | 6 | 7 & Text immer bis Frage 509_1 einblenden

Sie nehmen seit einiger Zeit aufgrund Ihrer Erkrankung des Nervensystems an dem neuen Versorgungsmodell (NPPV) teil. Die folgenden Fragen beziehen sich auf Ihre Erfahrungen in den letzten drei Monaten mit dieser Behandlungsform, das heißt die Monate [Monate].

PROG: alle Indikationen

Im Folgenden ist mit „Arzt / (Psycho-)Therapeut / Neurologe / Psychiater“ immer auch die weibliche Form „Ärztin / (Psycho-)Therapeutin / Neurologin / Psychiaterin“ eingeschlossen.

509n (T0_n.a.) PROG: Indikation = 1 | 2 | 4

Nehmen Sie für Ihre Behandlung in regelmäßigen Abständen Termine bei Ihrem Bezugsarzt / Bezugspsychotherapeuten wahr?

PROG: Indikation = 3 | 5 | 6 | 7

Nehmen Sie für Ihre Behandlung in regelmäßigen Abständen Termine bei Ihrem Bezugsarzt wahr?

❑ ja (1) ❑ nein (0)

509_1n (T0_n.a.) PROG: Frage 509 = 1 (ja)

Wie häufig nehmen Sie regelmäßige Behandlungstermine wahr?

jede Woche	alle 2 Wochen	jeden Monat	alle 2-3 Monate	alle 4-6 Monate	seltener
1	2	3	4	5	6

511n PROG: Indikation = 1 | 2 | 4

Haben Sie in den letzten 3 Monaten Ihrer Behandlung Ihren Bezugsarzt / Bezugspsychotherapeuten gewechselt?

PROG: Indikation = 3 | 5 | 6 | 7

Haben Sie in den letzten 3 Monaten Ihrer Behandlung Ihren Bezugsarzt gewechselt?

❑ ja (1) ❑ nein (0)

511_1 PROG: Frage 511 = 1 (ja)

nämlich _____________ Mal (numerisch, dreistellig, > 0)

PROG: Indikation = 1 | 2 | 4; auf jeder Seite bis Frage 503 anzeigen

Bitte beziehen Sie die folgenden Fragen auf Ihren Bezugsarzt / Bezugspsychotherapeuten.

PROG: Indikation = 3 | 5 | 6 | 7; auf jeder Seite bis Frage 503 anzeigen

Bitte beziehen Sie die folgenden Fragen auf Ihren Bezugsarzt.

PROG: kein Filter

Wie schätzen Sie die Qualität der Behandlung in den letzten 3 Monaten ein?

501n (T0_n.a.) PROG: Indikation = 1 | 2 | 4

In meiner Behandlung nimmt sich mein Arzt / Psychotherapeut immer genug Zeit für mich.

PROG: Indikation = 3 | 5 | 6 | 7

In meiner Behandlung nimmt sich mein Arzt immer genug Zeit für mich.

stimmt überhaupt nicht	stimmt nicht	weder noch	stimmt	stimmt voll und ganz
1	2	3	4	5

502n (T0_n.a.)

PROG: kein Filter

In meiner Behandlung fühle ich mich sehr gut betreut.

stimmt überhaupt nicht	stimmt nicht	weder noch	stimmt	stimmt voll und ganz
1	2	3	4	5

503n (T0_n.a.)

Meine Behandlung deckt meine Bedürfnisse voll und ganz ab.

stimmt überhaupt nicht	stimmt nicht	weder noch	stimmt	stimmt voll und ganz
1	2	3	4	5

Teil des neuen Versorgungsmodells in NPPV sind verschiedene Angebote und die Möglichkeit an Gruppen oder Online-Selbsthilfe (Novego) zur Unterstützung der Behandlung teilzunehmen. Wir möchten nun von Ihnen wissen, wie Sie die Behandlung im neuen Versorgungsmodell bewerten und welche Angebote Sie wahrnehmen.

600_3

PROG: Indikation = 1 | 2 | 4

In meiner Behandlung in NPPV hat mir mein Arzt / Psychotherapeut sinnvolle therapeutische Angebote empfohlen.

PROG: Indikation = 3 | 5 | 6 | 7

In meiner Behandlung in NPPV hat mir mein Arzt sinnvolle therapeutische Angebote empfohlen.

stimmt überhaupt nicht	stimmt nicht	weder noch	stimmt	stimmt voll und ganz
1	2	3	4	5

600_4

PROG: Indikation = 1 | 2 | 4

In meiner Behandlung in NPPV hat mir mein Arzt / Psychotherapeut ausführlich erklärt, was ich bei Notfällen und Krisen, zum Beispiel, wenn es mir plötzlich schlechter geht, tun soll.

PROG: Indikation = 3 | 5 | 6 | 7

In meiner Behandlung in NPPV hat mir mein Arzt ausführlich erklärt, was ich bei Notfällen und Krisen, zum Beispiel wenn es mir plötzlich schlechter geht, tun soll.

stimmt überhaupt nicht	stimmt nicht	weder noch	stimmt	stimmt voll und ganz
1	2	3	4	5

600_5

PROG: Indikation = 1 | 2 | 4

In meiner Behandlung in NPPV hat mich mein Arzt / Psychotherapeut ausführlich informiert, dass ich in Notfällen und Krisen schnell einen Termin bei ihm bekomme.

PROG: Indikation = 3 | 5 | 6 | 7

In meiner Behandlung in NPPV hat mich mein Arzt ausführlich informiert, dass ich in Notfällen und Krisen schnell einen Termin bei ihm bekomme.

stimmt überhaupt nicht	stimmt nicht	weder noch	stimmt	stimmt voll und ganz
1	2	3	4	5

504n (T0_n.a.)

PROG: Indikation = 1 | 2 | 4

In meiner Behandlung in NPPV erklärte mir mein Arzt / Psychotherapeut meinen Therapieverlauf einfach und verständlich.

PROG: Indikation = 3 | 5 | 6 | 7

In meiner Behandlung in NPPV erklärte mir mein Arzt meinen Therapieverlauf einfach und verständlich.

stimmt überhaupt nicht	stimmt nicht	weder noch	stimmt	stimmt voll und ganz
1	2	3	4	5

510n (T0_n.a.)

PROG: Indikation = 1 | 2 | 4

In meiner Behandlung in NPPV arbeiten verschiedene Ärzte / Psychotherapeuten gut zusammen und informieren sich gegenseitig.

PROG: Indikation = 3 | 5 | 6 | 7

In meiner Behandlung in NPPV arbeiten verschiedene Ärzte gut zusammen und informieren sich gegenseitig.

stimmt überhaupt nicht	stimmt nicht	weder noch	stimmt	stimmt voll und ganz
1	2	3	4	5

510_1n (T0_n.a.)

PROG: Indikation = 1 | 2 | 4

In meiner Behandlung in NPPV vermittelt mich mein Arzt / Psychotherapeut bei Bedarf an kompetente Stellen weiter (z. B. Physio- / Ergotherapie, anderer Facharzt).

PROG: Indikation = 3 | 5 | 6 | 7

In meiner Behandlung in NPPV vermittelt mich mein Arzt bei Bedarf an kompetente Stellen weiter (z.B. Physio- / Ergotherapie, anderer Facharzt).

stimmt überhaupt nicht	stimmt nicht	weder noch	stimmt	stimmt voll und ganz
1	2	3	4	5

Gruppenangebote

601 PROG: Indikation = 1 | 2 | 4

Wurde Ihnen von Ihrem Arzt / Psychotherapeuten ein Gruppenangebot empfohlen?

PROG: Indikation = 3 | 5 | 6 | 7

Wurde Ihnen von Ihrem Arzt ein Gruppenangebot empfohlen?

Ja (1) / nein (0)

Wie wurden Sie über die Gruppenangebote informiert?

601_1 PROG: Frage 601 = 1 (ja) & Indikation = 1 | 2 | 4

Mein Arzt / Psychotherapeut hat mir ausführlich den Inhalt und Ablauf des Gruppenangebots beschrieben.

PROG: Frage 601 = 1 (ja) & Indikation = 3 | 5 | 6 | 7

Mein Arzt hat mir ausführlich den Inhalt und Ablauf des Gruppenangebots beschrieben.

stimmt überhaupt nicht	stimmt nicht	weder noch	stimmt	stimmt voll und ganz
1	2	3	4	5

601_2 PROG: Frage 601 = 1 (ja) & Indikation = 1 | 2 | 4

Mein Arzt / Psychotherapeut hat mir genau erläutert, warum das Gruppenangebot für mich hilfreich ist.

PROG: Frage 601 = 1 (ja) & Indikation = 3 | 5 | 6 | 7

Mein Arzt hat mir genau erläutert, warum das Gruppenangebot für mich hilfreich ist.

stimmt überhaupt nicht	stimmt nicht	weder noch	stimmt	stimmt voll und ganz
1	2	3	4	5

PROG: Frage 601 = 1 (ja)

Welche Gruppenangebote wurden Ihnen empfohlen und haben Sie diese bereits wahrgenommen?

PROG: Mehrfachantwort möglich

	Gruppenangebot	empfohlen	wahrgenommen
**601_31**<br>**601_32**	Gruppe für Betroffene	ja (1) / nein (0)	ja (1) / nein (0)
**601_41**<br>**601_42**	Gruppe für Angehörige	ja (1) / nein (0)	ja (1) / nein (0)

601_10 PROG: Frage 601 = 1 (ja) & mind. einmal (Frage 601_x1 = 1 (ja) & Frage 601_x2 = 0 (nein))

Sie haben angegeben, dass Sie mindestens an einem Angebot, das Ihnen empfohlen wurde, noch nicht teilgenommen haben. Haben Sie noch vor das Angebot wahrzunehmen?

ja (1) / nein (0)

PROG: PROG: Frage 601 = 1 (ja) & Frage 601_10 = 0 (nein)

Welche Gründe haben Sie dafür?

PROG: Mehrfachantwort möglich

❑ Ich habe kein Interesse daran. (1 genannt; 0 nicht genannt)

❑ Der Aufwand ist mir zu groß. (1 genannt; 0 nicht genannt)

❑ Der Weg ist mir zu weit. (1 genannt; 0 nicht genannt)

❑ Ich glaube nicht, dass mir das Angebot hilft. (1 genannt; 0 nicht genannt)

❑ Ich möchte meine Probleme nicht vor anderen Patienten besprechen. (1 genannt; 0 nicht genannt)

❑ anderer Grund (1 genannt; 0 nicht genannt)

PROG: Frage 601_13 = 1 (anderer Grund)

Und zwar: _____________

Sie haben an mindestens einem Gruppenangebot teilgenommen. Bitte geben Sie an, inwiefern Sie den Aussagen zustimmen.

601_5 PROG: Frage 601 = 1 (ja) & mind. einmal (Frage 601_x2 = 1 (ja))

Das Gruppenangebot hat mir sehr geholfen, im Alltag mit meiner Erkrankung zurecht zu kommen.

stimmt überhaupt nicht	stimmt nicht	weder noch	stimmt	stimmt voll und ganz
1	2	3	4	5

601_6 PROG: Frage 601 = 1 (ja) & mind. einmal (Frage 601_x2 = 1 (ja))

Durch das Gruppenangebot belastet mich meine Erkrankung deutlich weniger.

stimmt überhaupt nicht	stimmt nicht	weder noch	stimmt	stimmt voll und ganz
1	2	3	4	5

601_7 PROG: Frage 601 = 1 (ja) & mind. einmal (Frage 601_x2 = 1 (ja))

Durch das Gruppenangebot habe ich meine Erkrankung besser verstanden.

stimmt überhaupt nicht	stimmt nicht	weder noch	stimmt	stimmt voll und ganz
1	2	3	4	5

601_8 PROG: Frage 601 = 1 (ja) & mind. einmal (Frage 601_x2 = 1 (ja))

Der Austausch mit anderen Betroffenen beim Gruppenangebot hat mir sehr geholfen.

stimmt überhaupt nicht	stimmt nicht	weder noch	stimmt	stimmt voll und ganz

1	2	3	4	5

Online-Selbsthilfe

602 PROG: Indikation = 1 | 2 | 4

Wurde Ihnen von Ihrem Arzt / Psychotherapeuten eine Online-Selbsthilfe (Novego) empfohlen?

PROG: Indikation = 3 | 5 | 6 | 7

Wurde Ihnen von Ihrem Arzt eine Online-Selbsthilfe (Novego) empfohlen?

Ja (1) / nein (0)

Wie wurden Sie über die Online-Selbsthilfe informiert?

602_1 PROG: Frage 602 = 1 (ja) & Indikation = 1 | 2 | 4

Mein Arzt / Psychotherapeut hat mir ausführlich den Inhalt und Ablauf der Online-Selbsthilfe (Novego) beschrieben.

PROG: Frage 602 = 1 (ja) & Indikation = 3 | 5 | 6 | 7

Mein Arzt hat mir ausführlich den Inhalt und Ablauf des Online-Selbsthilfe (Novego) beschrieben.

stimmt überhaupt nicht	stimmt nicht	weder noch	stimmt	stimmt voll und ganz
1	2	3	4	5

602_2 PROG: Frage 602 = 1 (ja) & Indikation = 1 | 2 | 4

Mein Arzt / Psychotherapeut hat mir genau erläutert, warum die Online-Selbsthilfe (Novego) für mich hilfreich ist.

PROG: Frage 602 = 1 (ja) & Indikation = 3 | 5 | 6 | 7

Mein Arzt hat mir genau erläutert, warum die Online-Selbsthilfe (Novego) für mich hilfreich ist.

stimmt überhaupt nicht	stimmt nicht	weder noch	stimmt	stimmt voll und ganz
1	2	3	4	5

602_3 PROG: Frage 602 = 1 (ja)

Haben Sie das Angebot der Online-Selbsthilfe (Novego) bereits wahrgenommen?

❑ ja (1)

❑ nein, aber ich habe vor es wahrzunehmen (2)

❑ nein und ich habe nicht vor es wahrzunehmen (0)

602_3 1 PROG: Frage 602 = 1 (ja) & Frage 602_3 = 0 (nein)

Sie haben angegeben, dass Sie an der Online-Selbsthilfe (Novego), die Ihnen empfohlen wurde, nicht teilnehmen wollen.

Welche Gründe haben Sie dafür?

PROG: Mehrfachantwort möglich

❑ Ich habe kein Interesse daran. (1 genannt; 0 nicht genannt)

❑ Der Aufwand ist mir zu groß. (1 genannt; 0 nicht genannt)

❑ Ich glaube nicht, dass mir die Online-Selbsthilfe (Novego) hilft. (1 genannt; 0 nicht genannt)

❑ anderer Grund (1 genannt; 0 nicht genannt)

Und zwar: _____________

PROG: Frage 602 = 1 (ja) & Frage 602_3 = 1 (ja)

Sie haben bereits an einer Online-Selbsthilfe (Novego) teilgenommen. Bitte geben Sie an, inwiefern Sie den folgenden Aussagen zustimmen.

602_4 PROG: Frage 602 = 1 (ja) & Frage 602_3 = 1 (ja)

Die Online-Selbsthilfe half mir sehr, im Alltag mit meiner Erkrankung zurecht zu kommen.

stimmt überhaupt nicht	stimmt nicht	weder noch	stimmt	stimmt voll und ganz
1	2	3	4	5

602_5 PROG: Frage 602 = 1 (ja) & Frage 602_3 = 1 (ja)

Durch die Online-Selbsthilfe belastet mich meine Erkrankung deutlich weniger.

stimmt überhaupt nicht	stimmt nicht	weder noch	stimmt	stimmt voll und ganz
1	2	3	4	5

602_6 PROG: Frage 602 = 1 (ja) & Frage 602_3 = 1 (ja)

Durch die Online-Selbsthilfe verstehe ich meine Erkrankung besser.

stimmt überhaupt nicht	stimmt nicht	weder noch	stimmt	stimmt voll und ganz
1	2	3	4	5

Schübe
Quelle: eigen
PROG: Indikation =5

516n (T0_n. a.) PROG: Indikation = 5

Haben Sie in den letzten drei Monaten, das heißt [Monate], einen Schub erlebt?

❑ ja (1) ❑ nein (0)

**516_1n (T0_n.a.)**	PROG: Indikation = 5 & Frage 516 = 1 (Schub erlebt)<br>**Haben Sie in dieser Zeit eine Schubtherapie gemacht?**<br>❑ ja, ambulant bei meinem behandelnden Arzt (1)<br>❑ ja, in einem Krankenhaus (2)<br>❑ nein (0)

Entlassungsmanagement
Quelle: eigen

**512n (T0_n.a.)**	PROG: Indikation = 1 | 2 | 4<br>**Wie viele Nächte waren Sie <u>in den vergangenen 3 Monaten, d.h. in den Monaten [Monate]</u> aufgrund Ihrer seelischen Erkrankung zur stationären Behandlung in einem Krankenhaus?**<br>PROG: Indikation = 3 | 5 | 6 | 7<br>**Wie viele Nächte waren Sie <u>in den vergangenen 3 Monaten, d.h. in den Monaten [Monate]</u> aufgrund Ihrer Erkrankung des Nervensystems zur stationären Behandlung in einem Krankenhaus?**<br>____ Nächte (numerisch, 3-stellig, max: 120)
**512_1n (T0_n.a.)**	PROG: Frage 512 > 0 & Indikation = alle<br>**Versuchen Sie sich an die letzte Entlassung aus dem Krankenhaus zu erinnern.**<br>PROG: Indikation = 1 | 2 | 4<br>**Wie viel Zeit ist zwischen Ihrer Entlassung und Ihrem ersten Termin bei Ihrem niedergelassenen Arzt / Psychotherapeuten verstrichen?**<br>PROG: Indikation = 3 | 5 | 6 | 7<br>**Wie viel Zeit ist zwischen Ihrer Entlassung und Ihrem ersten Termin bei Ihrem niedergelassenen Arzt verstrichen?**

bis zu 7 Tage	1 bis 2 Wochen	3 bis 4 Wochen	1 bis 3 Monate	länger als 3 Monate
1	2	3	4	5

Behandlungsabbrüche

Quelle: eigen

PROG: teilweise nach Indikation gefiltert

PROG: kein Filter

Manchmal hat man das Gefühl, dass eine Behandlung nicht so gut hilft oder einem beispielsweise aufgrund von Nebenwirkungen nicht guttut. Manche Patienten brechen deshalb eine Therapie <u>gegen den ärztlichen Rat</u> ab. Zum Beispiel gehen Sie zu vorgesehenen Folgeterminen nicht mehr hin oder nehmen die Medikamente nicht mehr wie vereinbart.

**513n (T0_n.a.)**	PROG: Indikation = 1 | 2 | 4<br>**Haben Sie <u>in den letzten 3 Monaten</u> eine Behandlung Ihrer seelischen Erkrankung gegen ärztlichen Rat abgebrochen?**<br>PROG: Indikation = 3 | 5 | 6 | 7<br>**Haben Sie <u>in den letzten 3 Monaten</u> eine Behandlung Ihrer Erkrankung des Nervensystems gegen ärztlichen Rat abgebrochen?**<br>❑... ja (1) ❑... nein (0)

Fragen zu Krisensituationen
Quelle: eigen

PROG: teilweise nach Indikation gefiltert

	PROG: Indikation = 1 | 2 | 4, letzte drei Monate angeben<br>**Manchmal gibt es bei einer Erkrankung Zeiten, in denen es einem plötzlich schlechter geht und man schnell einen Termin beim Arzt / Psychotherapeuten braucht.**<br>PROG: Indikation = 3 | 5 | 6 | 7<br>**Manchmal gibt es bei einer Erkrankung Zeiten, in denen es einem plötzlich schlechter geht und man schnell einen Termin beim Arzt braucht.**<br>PROG: kein Filter<br>**Denken Sie bitte für die folgenden Fragen <u>an die letzten 3 Monate, d.h. [Monate]</u> zurück.**
**515n (T0_n.a.)**	PROG: Indikation = 1 | 2 | 4<br>**Gab es für Sie <u>in den letzten 3 Monaten</u> einen Zeitpunkt, an dem Sie aufgrund der Verschlechterung Ihrer seelischen Erkrankung schnell einen Behandlungstermin benötigten?**<br>PROG: Indikation = 3 | 5 | 6 | 7<br>**Gab es für Sie <u>in den letzten 3 Monaten</u> einen Zeitpunkt, an dem Sie aufgrund der Verschlechterung Ihrer Erkrankung des Nervensystems schnell einen Behandlungstermin benötigten?**<br>❑... ja (1) ❑... nein (0)
**515_1n (T0_n.a.)**	PROG: Frage 515 = 1 (ja), Indikation = 1 | 2 | 4<br>**Wie lange mussten Sie auf diesen Behandlungstermin bei <u>Ihrem Bezugsarzt / Bezugspsychotherapeuten</u> warten?**<br>PROG: Frage 515 = 1 (ja), Indikation = 3 | 5 | 6 | 7<br>**Wie lange mussten Sie auf diesen Behandlungstermin bei <u>Ihrem Bezugsarzt</u> warten?**

Bis zu einem Tag	2-3 Tage	4-7 Tage	7-14 Tage	länger als 14 Tage
1	2	3	4	5

**515_3n**	PROG: Frage 515 = 1 (ja)<br>**Haben Sie aufgrund von zu langen Wartezeiten auf einen Termin eine Notaufnahme aufgesucht?**

**(T0_n.a.)**	❑… ja (1)	❑… nein (0)

6. KG: Versorgungsqualität (indikationsspezifisch)

Seitenüberschrift: Versorgungsqualität

Qualität / Zufriedenheit / Bezugsarzt
Quelle: eigen

PROG: Extra Seite mit folgendem Text, nicht fett und größere Schrift

PROG: Gruppe = 2 & Indikation = 1 | 2 | 4
Auf den folgenden Seiten möchten wir gern mehr über die Behandlung Ihrer seelischen Erkrankung erfahren.

Es geht darum, wie Sie Ihre Behandlung erleben und wie zufrieden Sie mit Ihrer Behandlung sind. Wir sind dabei an Ihrer persönlichen Einschätzung interessiert. Es gibt keine richtigen oder falschen Angaben.
Bitte denken Sie daran, falls Sie den Fragebogen stellvertretend für jemanden ausfüllen, alle Fragen aus Sicht der erkrankten Person zu beantworten.

PROG: Gruppe = 2 & Indikation = 3 | 5 | 6 | 7
Auf den folgenden Seiten möchten wir gern mehr über die Behandlung Ihrer Erkrankung des Nervensystems erfahren.

Es geht darum, wie Sie Ihre Behandlung erleben und wie zufrieden Sie mit Ihrer Behandlung sind. Wir sind dabei an Ihrer persönlichen Einschätzung interessiert. Es gibt keine richtigen oder falschen Angaben.
Bitte denken Sie daran, falls Sie den Fragebogen stellvertretend für jemanden ausfüllen, alle Fragen aus Sicht der erkrankten Person zu beantworten.

PROG: teilweise Filter nach Indikation und nach Gruppe

PROG: Gruppe = 2 & Indikation = 1 | 2 | 4, auf jeder Seite bis Frage 509_1 anzeigen

Die folgenden Fragen beziehen sich auf Ihre Behandlung der seelischen Erkrankung.

PROG: Gruppe = 2 & Indikation = 3 | 5 | 6 | 7, auf jeder Seite bis Frage 509_1 anzeigen

Die folgenden Fragen beziehen sich auf Ihre Behandlung der Erkrankung des Nervensystems.

PROG: alle

Im Folgenden ist mit „Arzt / (Psycho-)therapeut / Neurologe / Psychiater“ immer auch die weibliche Form „Ärztin / (Psycho-)therapeutin / Neurologin / Psychiaterin“ eingeschlossen.

500 PROG: Indikation = 1 | 2 | 4

Bei welchem Arzt / Psychotherapeuten waren Sie <u>in den letzten 3 Monaten</u> mit Ihrer seelischen Erkrankung vorwiegend, d.h. die meiste Zeit, in Behandlung?

PROG: Indikation = 3 | 5 | 6 | 7

Bei welchem Arzt / Psychotherapeuten waren Sie <u>in den letzten 3 Monaten</u> mit Ihrer Erkrankung des Nervensystems vorwiegend, d. h. die meiste Zeit, in Behandlung?

❑ Hausarzt (1)

❑ Psychiater (2)

❑ Neurologe oder Nervenarzt (3)

❑ Psychotherapeut (4)

❑ anderer Arzt / Therapeut (5)

nämlich: __________________

509 PROG: Indikation = 1 | 2 | 4

Nehmen Sie für Ihre Behandlung in regelmäßigen Abständen Termine bei diesem Arzt / Psychotherapeuten wahr?

PROG: Indikation = 3 | 5 | 6 | 7

Nehmen Sie für Ihre Behandlung in regelmäßigen Abständen Termine bei diesem Arzt wahr?

❑ ja (1) ❑ nein (0)

509_1 PROG: Frage 509 = 1 (ja)

Wie häufig nehmen Sie regelmäßige Behandlungstermine wahr?

jede Woche	alle 2 Wochen	jeden Monat	alle 2-3 Monate	alle 4-6 Monate	seltener
1	2	3	4	5	6

511 PROG: Indikation = 1 | 2 | 4

Haben Sie in den letzten 3 Monaten Ihrer Behandlung Ihren Arzt / Psychotherapeuten, zu dem Sie vorwiegend gehen, gewechselt?

PROG: Indikation = 3 | 5 | 6 | 7

Haben Sie in den letzten 3 Monaten Ihrer Behandlung Ihren Arzt, zu dem Sie vorwiegend gehen, gewechselt?

❑ ja (1) ❑ nein (0)

511_1 PROG: Frage 511 = 1 (ja)

nämlich ______________ Mal (numerisch, dreistellig, > 0)

PROG: Indikation = 1 | 2 | 4; auf jeder Seite bis Frage 505 anzeigen

Bitte beziehen Sie die folgenden Fragen auf diesen Arzt / Psychotherapeuten, bei dem Sie vorwiegend in Behandlung waren.

PROG: Indikation = 3 | 5 | 6 | 7; auf jeder Seite bis Frage 505 anzeigen

Bitte beziehen Sie die folgenden Fragen auf diesen Arzt, bei dem Sie vorwiegend in Behandlung waren.

PROG: kein Filter

Wie schätzen Sie die Qualität der Behandlung in den letzten 3 Monaten ein?

501 PROG: Indikation = 1 | 2 | 4

In meiner Behandlung nimmt sich mein Arzt / Psychotherapeut immer genug Zeit für mich.

PROG: Indikation = 3 | 5 | 6 | 7

	**In meiner Behandlung nimmt sich mein Arzt immer genug Zeit für mich.**

stimmt überhaupt nicht	stimmt nicht	weder noch	stimmt	stimmt voll und ganz
1	2	3	4	5

502 PROG: kein Filter

In meiner Behandlung fühle ich mich sehr gut betreut.

stimmt überhaupt nicht	stimmt nicht	weder noch	stimmt	stimmt voll und ganz
1	2	3	4	5

503 **Meine Behandlung deckt meine Bedürfnisse voll und ganz ab.**

stimmt überhaupt nicht	stimmt nicht	weder noch	stimmt	stimmt voll und ganz
1	2	3	4	5

503_1 PROG: Indikation = 1 | 2 | 4

In meiner Behandlung hat mir mein Arzt / Psychotherapeut ausführlich erklärt, was ich bei Notfällen und Krisen, zum Beispiel wenn es mir plötzlich schlechter geht, tun soll.

PROG: Indikation = 3 | 5 | 6 | 7

In meiner Behandlung hat mir mein Arzt ausführlich erklärt, was ich bei Notfällen und Krisen, zum Beispiel wenn es mir plötzlich schlechter geht, tun soll.

stimmt überhaupt nicht	stimmt nicht	weder noch	stimmt	stimmt voll und ganz
1	2	3	4	5

503_2 PROG: Indikation = 1 | 2 | 4

In meiner Behandlung hat mich mein Arzt / Psychotherapeut ausführlich informiert, dass ich in Notfällen und Krisen schnell einen Termin bei ihm bekomme.

PROG: Indikation = 3 | 5 | 6 | 7

In meiner Behandlung hat mich mein Arzt ausführlich informiert, dass ich in Notfällen und Krisen schnell einen Termin bei ihm bekomme.

stimmt überhaupt nicht	stimmt nicht	weder noch	stimmt	stimmt voll und ganz
1	2	3	4	5

504 PROG: Indikation = 1 | 2 | 4

In meiner Behandlung erklärte mir mein Arzt / Psychotherapeut meinen Therapieverlauf einfach und verständlich.

PROG: Indikation = 3 | 5 | 6 | 7

In meiner Behandlung erklärte mir mein Arzt meinen Therapieverlauf einfach und verständlich.

stimmt überhaupt nicht	stimmt nicht	weder noch	stimmt	stimmt voll und ganz
1	2	3	4	5

510 PROG: Indikation = 1 | 2 | 4

In meiner Behandlung arbeiten verschiedene Ärzte / Psychotherapeuten gut zusammen und informieren sich gegenseitig.

PROG: Indikation = 3 | 5 | 6 | 7

In meiner Behandlung arbeiten verschiedene Ärzte gut zusammen und informieren sich gegenseitig.

stimmt überhaupt nicht	stimmt nicht	weder noch	stimmt	stimmt voll und ganz
1	2	3	4	5

510_1 PROG: Indikation = 1 | 2 | 4

In meiner Behandlung vermittelt mich mein Arzt / Psychotherapeut bei Bedarf an kompetente Stellen weiter (z. B. Physio- / Ergotherapie, anderer Facharzt).

PROG: Indikation = 3 | 5 | 6 | 7

In meiner Behandlung vermittelt mich mein Arzt bei Bedarf an kompetente Stellen weiter (z. B. Physio- / Ergotherapie, anderer Facharzt).

stimmt überhaupt nicht	stimmt nicht	weder noch	stimmt	stimmt voll und ganz
1	2	3	4	5

505 PROG: Indikation = 1 | 2 | 4

In meiner Behandlung empfahl mir mein Arzt / Psychotherapeut sinnvolle therapeutische Angebote.

PROG: Indikation = 3 | 5 | 6 | 7

In meiner Behandlung empfahl mir mein Arzt sinnvolle therapeutische Angebote.

stimmt überhaupt nicht	stimmt nicht	weder noch	stimmt	stimmt voll und ganz
1	2	3	4	5

Gruppenangebote

506 PROG: Indikation = 1 | 2 | 4

Wurden Ihnen in Ihrer Behandlung von Ihrem Arzt / Psychotherapeuten Gruppenangebote, die nicht in einem Krankenhaus stattfanden, zur Unterstützung der Therapie empfohlen?

PROG: Indikation = 3 | 5 | 6 | 7

Wurden Ihnen in Ihrer Behandlung von Ihrem Arzt Gruppenangebote, die nicht in einem Krankenhaus stattfanden, zur Unterstützung der Therapie empfohlen?

❑ ja (1) ❑ nein (0)

Wie wurden Sie über das Gruppenangebot informiert?

506_01 PROG: Frage 506 = 1 (ja) & Indikation = 1 | 2 | 4

Mein Arzt / Psychotherapeut beschrieb mir ausführlich den Inhalt und Ablauf des Gruppenangebots.

PROG: Frage 506 = 1 (ja) & Indikation = 3 | 5 | 6 | 7

Mein Arzt beschrieb mir ausführlich den Inhalt und Ablauf des Gruppenangebots.

stimmt überhaupt nicht	stimmt nicht	weder noch	stimmt	stimmt voll und ganz
1	2	3	4	5

506_02 PROG: Frage 506 = 1 (ja) & Indikation = 1 | 2 | 4

Mein Arzt / Psychotherapeut erläuterte mir genau, warum das Gruppenangebot für mich hilfreich ist.

PROG: Frage 506 = 1 (ja) & Indikation = 3 | 5 | 6 | 7

Mein Arzt erläuterte mir genau, warum das Gruppenangebot für mich hilfreich ist.

stimmt überhaupt nicht	stimmt nicht	weder noch	stimmt	stimmt voll und ganz
1	2	3	4	5

506_03 PROG: Frage 506 = 1 (ja) & Indikation = 1 | 2 | 4

Mein Arzt / Psychotherapeut bzw. das Praxisteam unterstützte mich sehr dabei, das Gruppenangebot wahrzunehmen.

PROG: Frage 506 = 1 (ja) & Indikation = 3 | 5 | 6 | 7

Mein Arzt bzw. das Praxisteam unterstützte mich sehr dabei, das Gruppenangebot wahrzunehmen

stimmt überhaupt nicht	stimmt nicht	weder noch	stimmt	stimmt voll und ganz
1	2	3	4	5

PROG: Frage 506 = 1 (ja)

Welche Gruppenangebote wurden Ihnen empfohlen und haben Sie diese wahrgenommen?

Gruppenangebot	empfohlen	wahrgenommen

**506_11**<br>**506_12**	Gruppenangebot für Betroffene	ja (1) / nein (0)	ja (1) / nein (0)
**506_21**<br>**506_22**	Gruppenangebot für Angehörige	ja (1) / nein (0)	ja (1) / nein (0)

506_5 PROG: Frage 506 = 1 (ja) & mind. einmal (Frage 506_x1 = 1 (ja) & Frage 506_x2 = 0 (nein))

506_6 **Sie haben angegeben, dass Sie mindestens an einem Gruppenangebot, das Ihnen empfohlen wurde, nicht teilgenommen haben.**

506_7 **Welche Gründe hatten Sie dafür?**

506_71 PROG: Mehrfachantwort möglich

506_8

506_8o

- ❑ Ich habe kein Interesse daran. (1 genannt; 0 nicht genannt)
- ❑ Der Aufwand ist mir zu groß. (1 genannt; 0 nicht genannt)
- ❑ Der Weg ist mir zu weit. (1 genannt; 0 nicht genannt)
- ❑ Ich glaube nicht, dass mir das Angebot hilft. (1 genannt; 0 nicht genannt)
- ❑ Ich möchte meine Probleme nicht vor anderen Patienten besprechen. (1 genannt; 0 nicht genannt)
- ❑ anderer Grund (1 genannt; 0 nicht genannt)

PROG: Frage 506_8 = 1 (anderer Grund)

Und zwar: _____________

PROG: Frage 506 = 1 (ja) & mind. einmal (Frage 506_x2 = 1 (ja))

Sie haben an mindestens einem Gruppenangebot teilgenommen. Bitte geben Sie an, inwiefern Sie den folgenden Aussagen zustimmen.

506_9 PROG: Frage 506 = 1 (ja) & mind. einmal (Frage 506_x2 = 1 (ja))

Das Gruppenangebot hat mir sehr geholfen, im Alltag mit meiner Erkrankung zurecht zu kommen.

stimmt überhaupt nicht	stimmt nicht	weder noch	stimmt	stimmt voll und ganz
1	2	3	4	5

506_010 PROG: Frage 506 = 1 (ja) & mind. einmal (Frage 506_x2 = 1 (ja))

Durch das Gruppenangebot belastet mich meine Erkrankung deutlich weniger.

stimmt überhaupt nicht	stimmt nicht	weder noch	stimmt	stimmt voll und ganz
1	2	3	4	5

506_011 PROG: Frage 506 = 1 (ja) & mind. einmal (506_x2 = 1 (ja))

Durch das Gruppenangebot verstehe ich meine Erkrankung besser.

stimmt überhaupt nicht	stimmt nicht	weder noch	stimmt	stimmt voll und ganz
1	2	3	4	5

506_012 PROG: Frage 506 = 1 (ja) & mind. einmal (Frage 506_x2 = 1 (ja))

Der Austausch mit anderen Betroffenen beim Gruppenangebot hat mir sehr geholfen.

stimmt überhaupt nicht	stimmt nicht	weder noch	stimmt	stimmt voll und ganz
1	2	3	4	5

Onlineangebote

507 PROG: kein Filter

Wurde Ihnen in ihrer Behandlung Online-Selbsthilfe zur Unterstützung der Therapie empfohlen?

❑ ja (1) ❑ nein (0)

Wie wurden Sie über die Online-Selbsthilfe informiert?

507_01 PROG: Frage 507 = 1 (ja) & Indikation = 1 | 2 | 4

Mein Arzt / Psychotherapeut beschrieb mir ausführlich den Inhalt und Ablauf der Online-Selbsthilfe.

PROG: Frage 507 = 1 (ja) & Indikation = 3 | 5 | 6 | 7

Mein Arzt beschrieb mir ausführlich den Inhalt und Ablauf der Online-Selbsthilfe.

stimmt überhaupt nicht	stimmt nicht	weder noch	stimmt	stimmt voll und ganz
1	2	3	4	5

507_02 PROG: Frage 507 = 1 (ja) & Indikation = 1 | 2 | 4

Mein Arzt / Psychotherapeut erläuterte mir genau, warum die Online-Selbsthilfe für mich hilfreich ist.

PROG: Frage 507 = 1 (ja) & Indikation = 3 | 5 | 6 | 7

Mein Arzt erläuterte mir genau, warum die Online-Selbsthilfe für mich hilfreich ist.

stimmt überhaupt nicht	stimmt nicht	weder noch	stimmt	stimmt voll und ganz
1	2	3	4	5

507_1 PROG: Frage 507 = 1 (ja)

Haben Sie dieses Angebot der Online-Selbsthilfe wahrgenommen?

❑ ja (1) ❑ nein (0)

507_5 PROG: Frage 507 = 1 (ja) & Frage 507_1 = 0 (nein)

507_6 **Sie haben angegeben, dass Sie an einer Online-Selbsthilfe, die Ihnen empfohlen wurde, nicht teilgenommen haben.**

507_7

507_8 **Welche Gründe haben Sie dafür?**

507_8o PROG: Mehrfachantwort möglich

- ❑ Ich habe kein Interesse daran. (1 genannt; 0 nicht genannt)
- ❑ Der Aufwand ist mir zu groß. (1 genannt; 0 nicht genannt)
- ❑ Ich glaube nicht, dass mir die Online-Selbsthilfe hilft. (1 genannt; 0 nicht genannt)
- ❑ anderer Grund (1 genannt; 0 nicht genannt)

PROG: Frage 507_8 = 1 (anderer Grund)

Und zwar: _____________

PROG: Frage 507 = 1 (ja) & Frage 507_1 = 1 (ja)

Sie haben bereits an einer Online-Selbsthilfe teilgenommen. Bitte geben Sie an, inwiefern Sie den folgenden Aussagen zustimmen.

507_9 PROG: Frage 507 = 1 (ja) & Frage 507_1 = 0 (nein)

Die Online-Selbsthilfe hat mir sehr geholfen, im Alltag mit meiner Erkrankung zurecht zu kommen.

stimmt überhaupt nicht	stimmt nicht	weder noch	stimmt	stimmt voll und ganz
1	2	3	4	5

507_010 PROG: Frage 507 = 1 (ja) & Frage 507_1 = 0 (nein)

Durch die Online-Selbsthilfe belastet mich meine Erkrankung deutlich weniger.

stimmt überhaupt nicht	stimmt nicht	weder noch	stimmt	stimmt voll und ganz
1	2	3	4	5

507_011 PROG: Frage 507 = 1 (ja) & Frage 507_1 = 0 (nein)

Durch die Online-Selbsthilfe verstehe ich meine Erkrankung besser.

stimmt überhaupt nicht	stimmt nicht	weder noch	stimmt	stimmt voll und ganz
1	2	3	4	5

Schübe
Quelle: eigen
PROG: Indikation =5

516 PROG: Indikation = 5

Haben Sie <u>in den letzten drei Monaten, d.h. [Monate]</u> einen Schub erlebt?

❑ ja (1) ❑ nein (0)

516_1 PROG: Indikation = 5 & Frage 516 = 1 (Schub erlebt)

Haben Sie in dieser Zeit eine Schubtherapie gemacht?

❑ ja, ambulant bei meinem behandelnden Arzt (1)

❑ ja, in einem Krankenhaus (2)

❑ nein (0)

Entlassungsmanagement
Quelle: eigen

512 PROG: Indikation = 1 | 2 | 4

Wie viele Nächte waren Sie <u>in den vergangenen 3 Monaten, d.h. [Monate]</u> aufgrund Ihrer seelischen Erkrankung zur stationären Behandlung in einem Krankenhaus?

PROG: Indikation = 3 | 5 | 6 | 7

Wie viele Nächte waren Sie <u>in den vergangenen 3 Monaten, d.h. [Monate]</u> aufgrund Ihrer Erkrankung des Nervensystems zur stationären Behandlung in einem Krankenhaus?

____ Nächte (numerisch, 3-stellig, max: 120)

512_1 PROG: Frage 512 > 0 & Indikation = alle

Versuchen Sie sich an die letzte Entlassung aus dem Krankenhaus zu erinnern.

PROG: Indikation = 1 | 2 | 4

Wie viel Zeit ist zwischen Ihrer Entlassung und Ihrem ersten Termin bei Ihrem niedergelassenen Arzt / Psychotherapeuten verstrichen?

PROG: Indikation = 3 | 5 | 6 | 7

Wie viel Zeit ist zwischen Ihrer Entlassung und Ihrem ersten Termin bei Ihrem niedergelassenen Arzt verstrichen?

bis zu 7 Tage	1 bis 2 Wochen	3 bis 4 Wochen	1 bis 3 Monate	länger als 3 Monate
1	2	3	4	5

Behandlungsabbrüche

Quelle: eigen

PROG: teilweise nach Indikation gefiltert

PROG: kein Filter

Manchmal hat man das Gefühl, dass eine Behandlung nicht so gut hilft oder einem beispielsweise aufgrund von Nebenwirkungen nicht guttut. Manche Patienten brechen deshalb eine Therapie <u>gegen den</u>

ärztlichen Rat ab. Zum Beispiel gehen Sie zu vorgesehenen Folgeterminen nicht mehr hin oder nehmen die Medikamente nicht mehr wie vereinbart.

513 PROG: Indikation = 1 | 2 | 4

Haben Sie in den letzten 3 Monaten die Behandlung Ihrer seelischen Erkrankung gegen ärztlichen Rat abgebrochen?

PROG: Indikation = 3 | 5 | 6 | 7

Haben Sie in den letzten 3 Monaten die Behandlung Ihrer Erkrankung des Nervensystems gegen ärztlichen Rat abgebrochen?

❑... ja (1) ❑... nein (0)

Fragen zu Krisensituationen
Quelle: eigen

PROG: teilweise nach Indikation gefiltert

PROG: Indikation = 1 | 2 | 4

Manchmal gibt es bei einer Erkrankung Zeiten, in denen es einem plötzlich schlechter geht und man schnell einen Termin beim Arzt / Psychotherapeuten braucht.

PROG: Indikation = 3 | 5 | 6 | 7

Manchmal gibt es bei einer Erkrankung Zeiten, in denen es einem plötzlich schlechter geht und man schnell einen Termin beim Arzt braucht.

PROG: kein Filter

Denken Sie bitte für die folgenden Fragen an die letzten 3 Monate, d.h. [Monate] zurück.

515 PROG: Indikation = 1 | 2 | 4

Gab es für Sie in den letzten 3 Monaten einen Zeitpunkt, an dem Sie aufgrund der Verschlechterung Ihrer seelischen Erkrankung schnell einen Behandlungstermin benötigten?

PROG: Indikation = 3 | 5 | 6 | 7

Gab es für Sie in den letzten 3 Monaten einen Zeitpunkt, an dem Sie aufgrund der Verschlechterung Ihrer Erkrankung des Nervensystems schnell einen Behandlungstermin benötigten?

❑... ja (1) ❑... nein (0)

515_1 PROG: Frage 515 = 1 (ja), Indikation = 1 | 2 | 4

Wie lange mussten Sie auf diesen Behandlungstermin bei Ihrem niedergelassenen Arzt / Psychotherapeuten warten?

PROG: Frage 515 = 1 (ja), Indikation = 3 | 5 | 6 | 7

Wie lange mussten Sie auf diesen Behandlungstermin bei Ihrem niedergelassenen Arzt warten?

Bis zu einem Tag	2-3 Tage	4-7 Tage	7-14 Tage	länger als 14 Tage
1	2	3	4	5

**515_3**	PROG: Frage 515 = 1 (ja)
	**Haben Sie aufgrund von zu langen Wartezeiten auf einen Termin eine Notaufnahme aufgesucht?**
	❑... ja (1) ❑... nein (0)

7. Abschluss

**710**	PROG: kein Filter
	**Bitte geben Sie zum Abschluss noch an, wie Sie den Fragebogen ausgefüllt haben.**
	❑ vorwiegend alleine (1)
	❑ zusammen mit einer anderen Person (2)
	❑ vorwiegend eine andere Person (3)
**711o**	**Haben Sie noch weitere Anregungen für uns?**

VIELEN DANK FÜR IHRE TEILNAHME!

Hilfsvariablen zur Programmierung

Indikation = 1-7
Kodierung: 1 = Depression, 2 = Schizophrenie, schizotype oder wahnhafte Störungen, bipolare Störungen, 3 = Demenz; 4 = komplexe Traumafolgestörungen, 5 = Multiple Sklerose, 6 = Parkinson, 7 = Schlaganfall

Gruppe = 1;2
Kodierung: 1 = IG; 2 = KG

Einschub in Text beruhend auf Ausfülldatum:
[Monate] – die letzten 3 vollen Kalendermonate (z. B. „Januar, Februar und März"; wenn Ausfülldatum der 16.4.)
[Monat bis Monat] die letzten 6 vollen Kalendermonate (z. B. „Januar bis Juni"; wenn Ausfülldatum 13.7.)

Missings zulassen und kodieren: NULL
Kodiert im Pseudonym wird Gruppe und Indikation: Liste mit Pseudonymen + Indikation + Gruppe + Kennwort / Token
Speichern der Eingaben bei Abbruch: ja
Möglichkeit der Unterbrechung der Befragung: ja

Startseite

PROG: Gruppe = 1 (www.nppv-iges.de)

Herzlich Willkommen

Schön, dass Sie an der Befragung zur Qualität Ihrer Behandlung teilnehmen möchten. Sie helfen uns damit die Versorgung von Betroffenen zu verbessern.
Die Befragung findet im Rahmen der Versorgung Ihrer Erkrankung im Projekt zur Verbesserung der neurologisch-psychiatrischen und psychotherapeutischen Versorgung statt. Insgesamt werden Sie im Verlauf eines Jahres für 4 Befragungen kontaktiert.

Informationen zum Datenschutz

Ihre Daten werden streng nach den gesetzlichen Datenschutzbestimmungen verarbeitet. Es wird sichergestellt, dass die Daten der Befragung nicht mit Ihrer Person in Verbindung gebracht werden können. Zur Verknüpfung der vier Befragungszeitpunkte wird Ihnen ein Pseudonym zugewiesen. Nach Ende der letzten Befragung werden die Daten anonymisiert, so dass kein Personenbezug mehr herstellbar ist. Die Daten werden nur anonymisiert und in aggregierter Form veröffentlicht. Es finden keine Auswertungen für einzelne Personen statt. Nach Ablauf des Projektes werden die Daten unter Berücksichtigung der gesetzlichen Bestimmungen gelöscht.

Freiwilligkeit der Teilnahme

Ihre Teilnahme an der Befragung ist freiwillig und kann jederzeit ohne Angabe von Gründen beendet werden. Auch können Sie bis zum Zeitpunkt der Anonymisierung nach der letzten Befragung verlangen, dass alle von Ihnen erhobenen Daten gelöscht werden.
Weitere Informationen zur Teilnahme und zum Datenschutz finden Sie auch im Anschreiben Ihrer Krankenkasse.

Wenn Sie an der Befragung teilnehmen möchten und der beschriebenen Verarbeitung Ihrer Daten zustimmen, geben Sie bitte hier Ihr Passwort ein, das Sie von Ihrer Krankenkasse im Anschreiben erhalten haben:

PROG: Gruppe 2 (www.nppv-iges.de/KG)

Herzlich Willkommen

Schön, dass Sie an der Befragung zur Qualität Ihrer Behandlung teilnehmen möchten. Sie helfen uns damit die Versorgung von Betroffenen zu verbessern.
In dieser Befragung geht es um die Versorgung Ihrer seelischen und/oder neurologischen Erkrankung. Zurzeit werden Sie aufgrund dieser Erkrankung nach dem aktuellen Standard der Regelversorgung behandelt.

Um die Versorgung hierfür zu verbessern, fördert der Gesetzgeber neue Versorgungsprojekte im Rahmen des sogenannten *Innovationsfonds*. Eines dieser Projekte ist die Verbesserung der neurologischen-psychiatrischen und psychotherapeutischen Versorgung (NPPV).
Ziel von NPPV ist es, die Behandlung von psychischen und neurologischen Krankheiten zu verbessern. Dazu wird dem/r Patienten/in ein Bezugsarzt oder -therapeut bzw. eine Bezugsärztin oder -therapeutin an die Seite gestellt, der/die die verschiedenen Behandler/innen vernetzt und die Versorgung koordiniert.
Wir möchten herausfinden, ob NPPV die Versorgung spürbar verbessern kann und benötigen dazu Ihre Hilfe: Sie sind eingeladen an dieser Befragung teilzunehmen und durch Ihre Erfahrungen mit der bisherigen Regelversorgung einen wichtigen Beitrag zur Beurteilung der Versorgungsformen beizusteuern.
Die Ergebnisse dieser Befragungen können dazu führen, dass zukünftig alle gesetzlich Krankenversicherten von neuen Versorgungsformen profitieren.

Einverständniserklärung zur Datenverarbeitung
Für den Landesteil Nordrhein wird ein Projekt zur Verbesserung der neurologisch-psychiatrischen und psychotherapeutischen Versorgung (NPPV-Projekt) durchgeführt.
Ich nehme an dem NPPV-Projekt nicht teil. Ich bin aber durch das ausführliche Anschreiben meiner Krankenkasse eingehend darüber informiert worden, dass für alle gesetzlich krankenversicherten Patienten mit entsprechender Erkrankung ein Nutzen aus dem NPPV-Projekt nur festgestellt werden kann, wenn Daten von teilnehmenden Patienten mit Daten von nicht teilnehmenden Patienten (sog. Kontrollgruppe) verglichen werden.
Ich komme für eine Befragung in der sog. Kontrollgruppe in Betracht und stehe für diese zur Verfügung.
Ich habe darüber hinaus den Text der nachfolgenden Datenschutzerklärung gelesen und verstanden.
Datenschutzerklärung
Mir ist bekannt, dass personenbezogene Daten, d. h. Stammdaten (Name, Anschrift, Geburtsdatum etc.) sowie erforderliche Gesundheitsdaten (medizinische Behandlungsdaten) über mich bei meiner Krankenkasse erhoben, gespeichert und verarbeitet werden. Die Verwendung der Daten erfolgt nach gesetzlichen Bestimmungen. Ich erteile hiermit ausdrücklich die folgenden datenschutzrechtlichen Einwilligungserklärungen.
Ich erkläre mich damit einverstanden, dass meine Krankenkasse mir einen Fragebogen zur wissenschaftlichen Evaluation (Auswertung) zur Verfügung stellt, den ich online ausfülle und dessen Antworten pseudonymisiert (d. h. mit einer Kennung, aber ohne meinen Namen, Kontaktdaten oder sonstigen Angaben) bei der mit der wissenschaftlichen Evaluation beauftragten IGES Institut GmbH, Friedrichstraße 180, 10117 Berlin – vertretungsberechtigte Geschäftsführer: Prof. Dr. Bertram Häussler (Vorsitzender der Geschäftsführung), Dr. Martin Albrecht, Christoph Gipp, Hans-Dieter Nolting – gespeichert und verarbeitet werden. Die Befragung erfolgt ausschließlich bezogen auf eventuell bei mir vorliegende Krankheitsbilder, die auch Gegenstand des NPPV-Projekts sind; Rückschlüsse auf meine Person sind ausgeschlossen.

Ich bin darüber aufgeklärt worden, dass ich jederzeit meine Einwilligung zur Datenverarbeitung widerrufen kann und bis zum Zeitpunkt der Anonymisierung (nach Abschluss der letzten Befragung) verlangen kann, dass alle von mir erhobenen Daten gelöscht werden.

Für gesetzliche Vertreter/innen, Betreuer/innen bzw. Vorsorgebevollmächtigte:
Ich habe die obigen Ausführungen zur Einverständniserklärung zur Datenverarbeitung sowie die Datenschutzerklärung sorgfältig gelesen. Hiermit erkläre ich die Einwilligung des von mir betreuten bzw. vertretenen Patienten.

Wenn Sie an der Befragung teilnehmen möchten und der beschriebenen Verarbeitung Ihrer Daten zustimmen, geben Sie bitte hier Ihr Passwort ein, das Sie von Ihrer Krankenkasse im Anschreiben erhalten haben:

Übersicht über Ihre Befragungen

PROG: kein Filter

Insgesamt wird es vier Online-Befragungen geben, zu denen Sie jeweils von Ihrer Krankenkasse eingeladen werden. Die zweite Befragung findet ca. 3 Monate, die dritte ca. 6 Monate und die vierte ca. 12 Monate nach der ersten Befragung statt.
Bitte klicken Sie nachfolgend auf die nächst verfügbare Befragung, um mit dem Ausfüllen des Fragebogens zu beginnen.
PROG: Nur die aktuelle Befragung sollte auswählbar sein.
Befragung 1
Befragung 2
Befragung 3
Befragung 4

Einleitungstext

PROG: Gruppe = 1

	Lieber Teilnehmer, liebe Teilnehmerin,<br>Wir freuen uns sehr, dass Sie sich entschieden haben, an dem neuen Versorgungsmodell für Menschen mit psychischen und neurologischen Erkrankungen (NPPV) teilzunehmen. Um herauszufinden, wie gut Ihnen die neue Versorgungsform hilft und wie Sie diese bewerten, werden wir Ihnen nachfolgend Fragen zu Ihrem Gesundheitszustand und Ihren Erfahrungen mit der neuen Versorgungsform stellen. Einige der Fragen könnten Ihnen aus der letzten Befragung bekannt vorkommen. Wir würden uns sehr freuen, wenn Sie auch an dieser vierten und damit letzten Befragung im Rahmen der Untersuchung teilnehmen.<br><br>Das Ausfüllen wird etwa 30 Minuten in Anspruch nehmen. Sie können die Befragung jederzeit unterbrechen und zu einem späteren Zeitpunkt mit demselben Passwort fortsetzen. Sie können selbst entscheiden, ob Sie den Fragebogen alleine oder gemeinsam mit einer Bezugsperson ausfüllen. Die Bezugsperson kann die Beantwortung der Fragen auch vollständig übernehmen. Bitte geben Sie am Ende der Befragung an, wer den Fragebogen vorwiegend ausgefüllt hat.<br><br>Danke, dass Sie sich die Zeit nehmen die Fragen zu beantworten.<br><br>Bei Fragen zu der Befragung wenden Sie sich bitte an:<br>Tobias Woköck<br>Tel:<br>Email:

PROG: Gruppe = 2

	Lieber Teilnehmer, liebe Teilnehmerin,<br>Wir freuen uns sehr, dass Sie sich bereit erklären, diesen Fragebogen zu der Versorgung Ihrer Erkrankung auszufüllen. Sie helfen uns damit, die Versorgung ein Stück besser zu machen.<br>Um herauszufinden, wie gut Ihnen Ihre Versorgung hilft und wie Sie diese bewerten, werden wir Ihnen nachfolgend Fragen zu Ihrem Gesundheitszustand und Ihren Erfahrungen mit der Versorgung Ihrer Erkrankung stellen. Einige der Fragen könnten Ihnen aus der letzten Befragung bekannt vorkommen. Wir würden uns sehr freuen, wenn Sie auch an dieser vierten und damit letzten Befragung im Rahmen der Untersuchung teilnehmen.<br><br>Das Ausfüllen wird etwa 30 Minuten in Anspruch nehmen. Sie können die Befragung jederzeit unterbrechen und zu einem späteren Zeitpunkt mit demselben Passwort fortsetzen. Sie können selbst entscheiden, ob Sie den Fragebogen alleine oder gemeinsam mit einer Bezugsperson ausfüllen. Die Bezugsperson kann die Beantwortung der Fragen auch vollständig übernehmen. Bitte geben Sie am Ende der Befragung an, wer den Fragebogen vorwiegend ausgefüllt hat.<br><br>Danke, dass Sie sich die Zeit nehmen die Fragen zu beantworten.

	Bei Fragen zur Befragung wenden Sie sich bitte an:<br>Tobias Woköck<br>Tel:<br>Email:

PROG: Datum und Zeitstempel erfassen

1. Soziodemographie

Seitenüberschrift: Angaben zur Person

PROG: kein Filter

	**Zum Beginn möchten wir Sie um zwei kurze Angaben zu Ihrer Person bitten.**<br>**Falls Sie den Fragebogen stellvertretend für jemanden ausfüllen, beantworten Sie bitte alle Fragen aus Sicht der erkrankten Person.**
**001**	**Wie ist Ihr Geburtsjahr?**<br>______ (numerisch, 4-stellig, min 1900)
**002**	**Welches ist Ihr Geschlecht?** ❑ männlich (1) ❑ weiblich (2)

2. Gesundheitsbezogene Lebensqualität (generisch)

Seitenüberschrift: Gesundheit & Lebensqualität

WHOQOL-BREF (26 Items):
Quelle: Hogrefe
Lizenz: gekauft

PROG: kein Filter

	**Die nun folgenden Fragen beschäftigen sich mit Ihrer Lebensqualität, Ihrer Gesundheit und anderen Bereichen Ihres Lebens. Wir möchten möglichst genau erfahren, wie es Ihnen im Allgemeinen und mit spezifischen Erkrankungen geht.**<br>**Bitte denken Sie daran, falls Sie den Fragebogen stellvertretend für jemanden ausfüllen, alle Fragen aus Sicht der erkrankten Person zu beantworten.**					
	**Bitte lesen Sie jede Frage, überlegen Sie, wie Sie sich in den letzten zwei Wochen gefühlt haben und geben Sie die Antwort an, die am ehesten auf Sie zutrifft.**					
		sehr schlecht	schlecht	mittelmäßig	gut	sehr gut
**101**	Wie würden Sie Ihre Lebensqualität beurteilen?	1	2	3	4	5
		sehr unzufrieden	unzufrieden	weder zufrieden noch unzufrieden	zufrieden	sehr zufrieden
**102**	Wie zufrieden sind Sie mit Ihrer Gesundheit?	1	2	3	4	5
	**In den folgenden Fragen geht es darum, wie stark Sie während der letzten zwei Wochen bestimmte Dinge erlebt haben.**					

		überhaupt nicht	ein wenig	mittelmäßig	ziemlich	äußerst
**103**	Wie stark werden Sie durch Schmerzen daran gehindert, notwendige Dinge zu tun?	1	2	3	4	5
**104**	Wie sehr sind Sie auf medizinische Behandlung angewiesen, um das tägliche Leben zu meistern?	1	2	3	4	5
**105**	Wie gut können Sie Ihr Leben genießen?	1	2	3	4	5
**106**	Betrachten Sie Ihr Leben als sinnvoll?	1	2	3	4	5
**107**	Wie gut können Sie sich konzentrieren?	1	2	3	4	5
**108**	Wie sicher fühlen Sie sich in Ihrem täglichen Leben?	1	2	3	4	5
**109**	Wie gesund sind die Umweltbedingungen in Ihrem Wohngebiet?	1	2	3	4	5
	**In den folgenden Fragen geht es darum, in welchem Umfang Sie <u>während der letzten zwei Wochen</u> bestimmte Dinge erlebt haben oder in der Lage waren, bestimmte Dinge zu tun.**					
		überhaupt nicht	eher nicht	halbwegs	überwiegend	völlig
**110**	Haben Sie genug Energie für das tägliche Leben?	1	2	3	4	5
**111**	Können Sie Ihr Aussehen akzeptieren?	1	2	3	4	5
**112**	Haben Sie genug Geld, um Ihre Bedürfnisse erfüllen zu können?	1	2	3	4	5
**113**	Haben Sie Zugang zu den Informationen, die Sie für das tägliche Leben brauchen?	1	2	3	4	5
**114**	Haben Sie ausreichend Möglichkeiten zu Freizeitaktivitäten?	1	2	3	4	5
		sehr schlecht	schlecht	mittelmäßig	gut	sehr gut
**115**	Wie gut können Sie sich fortbewegen?	1	2	3	4	5
	**In den folgenden Fragen geht es darum, wie zufrieden, glücklich oder gut Sie sich <u>während der letzten zwei Wochen</u> hinsichtlich verschiedener Aspekte Ihres Lebens gefühlt haben.**					

	sehr unzufrieden	unzufrieden	weder zufrieden noch unzufrieden	zufrieden	sehr zufrieden
**116** Wie zufrieden sind Sie mit Ihrem Schlaf?	1	2	3	4	5
**117** Wie zufrieden sind Sie mit Ihrer Fähigkeit, alltägliche Dinge erledigen zu können?	1	2	3	4	5
**118** Wie zufrieden sind Sie mit Ihrer Arbeitsfähigkeit?	1	2	3	4	5
**119** Wie zufrieden sind Sie mit sich selbst?	1	2	3	4	5
**120** Wie zufrieden sind Sie mit Ihren persönlichen Beziehungen?	1	2	3	4	5
**121** Wie zufrieden sind Sie mit Ihrem Sexualleben?	1	2	3	4	5
**122** Wie zufrieden sind Sie mit der Unterstützung durch Ihre Freunde?	1	2	3	4	5
**123** Wie zufrieden sind Sie mit Ihren Wohnbedingungen?	1	2	3	4	5
**124** Wie zufrieden sind Sie mit Ihren Möglichkeiten, Gesundheitsdienste in Anspruch nehmen zu können?	1	2	3	4	5
**125** Wie zufrieden sind Sie mit den Beförderungsmitteln, die Ihnen zur Verfügung stehen?	1	2	3	4	5
**Bei der folgenden Frage geht es darum, wie oft sich <u>während der letzten zwei Wochen</u> bei Ihnen negative Gefühle eingestellt haben, wie zum Beispiel Angst oder Traurigkeit.**					
	niemals	nicht oft	zeitweilig	oftmals	immer
**126** Wie häufig haben Sie negative Gefühle wie Traurigkeit, Verzweiflung, Angst oder Depression?	1	2	3	4	5
PROG: Auf jeder Seite mit diesen Items<br>Hogrefe Copyright					

3. Gesundheitliche Situation (indikationsspezifisch)

**Seitenüberschrift:** Gesundheit & Lebensqualität

Fragen zur Erkrankung

Quelle: eigen

PROG:

Teilweise Filter nach Indikation und Gruppe

[Monate]: die letzten 3 vollen Kalendermonate einfügen

PROG: Extra Seite mit folgendem Text, nicht fett und größere Schrift

PROG: Gruppe = 1 & Indikation = 1 | 2 | 4
Sie nehmen aufgrund einer seelischen Erkrankung an dem neuen Versorgungsmodell (NPPV) teil. Wir möchten im Folgenden gern mehr über Ihre Erfahrungen mit dieser Erkrankung erfahren.

Es geht darum, wie Sie Ihre Krankheit erleben und wie es Ihnen mit Ihrer seelischen Erkrankung im Alltag geht. Uns ist dabei Ihre persönliche Einschätzung wichtig. Es gibt keine richtigen oder falschen Angaben.
Bitte denken Sie daran, falls Sie den Fragebogen stellvertretend für jemanden ausfüllen, alle Fragen aus Sicht der erkrankten Person zu beantworten.

PROG: Gruppe = 1 & Indikation = 3 | 5 | 6 | 7
Sie nehmen aufgrund einer Erkrankung des Nervensystems an dem neuen Versorgungsmodell (NPPV) teil. Wir möchten im Folgenden gern mehr über Ihre Erfahrungen mit dieser Erkrankung erfahren.

Es geht darum, wie Sie Ihre Krankheit erleben und wie es Ihnen mit Ihrer Erkrankung des Nervensystems im Alltag geht. Uns ist dabei Ihre persönliche Einschätzung wichtig. Es gibt keine richtigen oder falschen Angaben.
Bitte denken Sie daran, falls Sie den Fragebogen stellvertretend für jemanden ausfüllen, alle Fragen aus Sicht der erkrankten Person zu beantworten.

PROG: Gruppe = 2 & Indikation = 1 | 2 | 4
Wir haben Sie aufgrund einer seelischen Erkrankung zu dieser Befragung eingeladen. Wir möchten im Folgenden gern mehr über Ihre Erfahrungen mit dieser Erkrankung erfahren.

Es geht darum, wie Sie Ihre Krankheit erleben und wie es Ihnen mit Ihrer seelischen Erkrankung im Alltag geht. Uns ist dabei Ihre persönliche Einschätzung wichtig. Es gibt keine richtigen oder falschen Angaben.
Bitte denken Sie daran, falls Sie den Fragebogen stellvertretend für jemanden ausfüllen, alle Fragen aus Sicht der erkrankten Person zu beantworten.

PROG: Gruppe = 2 & Indikation = 3 | 5 | 6 | 7
Wir haben Sie aufgrund einer Erkrankung des Nervensystems zu dieser Befragung eingeladen. Wir möchten im Folgenden gern mehr über Ihre Erfahrungen mit dieser Erkrankung erfahren.

Es geht darum, wie Sie Ihre Krankheit erleben und wie es Ihnen mit Ihrer Erkrankung des Nervensystems im Alltag geht. Uns ist dabei Ihre persönliche Einschätzung wichtig. Es gibt keine richtigen oder falschen Angaben.
Bitte denken Sie daran, falls Sie den Fragebogen stellvertretend für jemanden ausfüllen, alle Fragen aus Sicht der erkrankten Person zu beantworten.

PROG: Gruppe 1 & Indikation = 1 | 2 | 4

Sie nehmen aufgrund einer seelischen Erkrankung an dem neuen Versorgungsmodell (NPPV) teil. Die folgenden Fragen beziehen sich daher auf Ihre Erfahrungen mit dieser Erkrankung.

PROG: Gruppe 1 & Indikation = 3 | 5 | 6 | 7

Sie nehmen aufgrund einer Erkrankung des Nervensystems an dem neuen Versorgungsmodell (NPPV) teil. Die folgenden Fragen beziehen sich daher auf Ihre Erfahrungen mit dieser Erkrankung.

PROG: Gruppe 2 & Indikation = 1 | 2 | 4

Wir haben Sie aufgrund einer seelischen Erkrankung zu dieser Befragung eingeladen. Die folgenden Fragen beziehen sich daher auf Ihre Erfahrungen mit dieser Erkrankung.

PROG: Gruppe 1 & Indikation = 3 | 5 | 6 | 7

Wir haben Sie aufgrund einer Erkrankung des Nervensystems zu dieser Befragung eingeladen. Die folgenden Fragen beziehen sich daher auf Ihre Erfahrungen mit dieser Erkrankung.

302 PROG: kein Filter

Inwiefern hatten Sie <u>in den letzten 2 Wochen</u> aufgrund dieser Erkrankung körperliche und/oder seelische Beschwerden?

keine	leichte	mittlere	große
1	2	3	4

303 **Wie sehr fühlten Sie sich <u>in den letzten 2 Wochen</u> durch diese Erkrankung in Ihrem Alltag belastet?**

überhaupt nicht	kaum	etwas	ziemlich	sehr
1	2	3	4	5

304 **Wir möchten gern von Ihnen wissen, wie sich Ihre Erkrankung in der letzten Zeit verändert hat. Die folgenden Fragen beziehen sich daher auf <u>die letzten 3 Monate</u>, das heißt auf** [PROG: Monate]**.**

Hat sich Ihre Erkrankung <u>in den letzten 3 Monaten</u> verbessert, verschlechtert oder ist sie gleich geblieben?

deutlich verschlechtert	verschlechtert	gleich geblieben	verbessert	deutlich verbessert
1	2	3	4	5

305 PROG: Indikation = 1 | 2 | 4

Nehmen Sie zurzeit Medikamente gegen die seelische Erkrankung ein?

PROG: Indikation = 3 | 5 | 6 | 7

Nehmen Sie zurzeit Medikamente gegen die Erkrankung des Nervensystems ein?

❑… ja (1) ❑… nein (0)

4. Lebensqualität (indikationsspezifisch)

Seitenüberschrift: Gesundheit & Lebensqualität

Q-LES-Q 18 (Depression; Schizophrenie, schizotype oder wahnhafte Störungen, bipolare Störungen; Traumafolgestörungen)

Quelle: Ritsner, M., Kurs, R., Gibel, A., Ratner, Y. & Endicott, J. (2005). Validity of an abbreviated Quality of Life Enjoyment and Satisfaction Questionnaire (Q-LES-Q-18) for schizophrenia, schizoaffective,and mood disorder patients. Quality of Life research, 14, 1693-1703.

Endicott J, Nee J, Harrison W, Blumenthal R. Quality of Life Enjoyment and Satisfaction Questionnaire: a new measure. Psychopharmacol Bull. 1993;29(2):321-6

Rohenkohl, A., Ruppelt, F., Gallinat, J., Karow, A., Lüdecke, D., Nawara, L. A., ... Lambert, M. (2015). Erfassung der Lebensqualität bei PsychosepatientInnen – psychometrische Analyse des Q-LES-Q-18 Fragebogens

Lizenzfrei in Ritsner publiziert

Deutsche Übersetzung mit Rückübersetzung selbst gemacht

PROG: Indikation = 1 | 2 | 4 (Depression; Schizophrenie, schizotype oder wahnhafte Störungen, bipolare Störungen; Traumafolgestörungen)

PROG: Indikation = 1 | 2 | 4

In den folgenden Fragen geht es darum, wie es Ihnen in den letzten zwei Wochen in verschiedenen Bereichen Ihres Lebens ergangen ist.

	**Wie häufig…**	nie	selten	manch mal	häufig	immer
**1401**	… waren Sie bei sehr guter körperlicher Gesundheit?	1	2	3	4	5
**1402**	… waren Sie frei von Sorgen über Ihre körperliche Gesundheit?	1	2	3	4	5
**1403**	… fühlten Sie sich körperlich gut?	1	2	3	4	5
**1404**	… fühlten Sie sich voller Energie und Vitalität?	1	2	3	4	5
**1405**	… waren Sie zufrieden mit Ihrem Leben?	1	2	3	4	5
**1406**	… fühlten Sie sich glücklich oder fröhlich?	1	2	3	4	5
**1407**	… fühlten Sie sich in der Lage mit anderen zu kommunizieren?	1	2	3	4	5
**1408**	… fühlten Sie sich in der Lage sich zu Fuß, mit dem Auto, Bus, Bahn oder Fahrrad fortzubewegen, um Erledigungen zu machen?	1	2	3	4	5
**1409**	… fühlten Sie sich in der Lage, sich um sich selbst zu kümmern?	1	2	3	4	5
**1410**	… sind Sie Ihren Freizeitaktivitäten nachgegangen?	1	2	3	4	5

**1411**	… haben Sie sich auf die Freizeitaktivitäten konzentriert und ihnen Aufmerksamkeit geschenkt?	1	2	3	4	5
**1412**	Wenn bei Ihren Freizeitaktivitäten ein Problem auftauchte, wie oft konnten Sie es lösen oder damit ohne übermäßigen Stress umgehen?	1	2	3	4	5
**1413**	… haben Sie sich auf ein Zusammentreffen mit Freunden oder Verwandten gefreut?	1	2	3	4	5
**1414**	… hat es Ihnen Spaß gemacht, mit Kollegen oder Nachbarn zu sprechen?	1	2	3	4	5
**1415**	… haben Sie Zuneigung gegenüber einer oder mehrerer Personen gespürt?	1	2	3	4	5
**1416**	… haben Sie mit anderen Menschen gescherzt oder gelacht?	1	2	3	4	5
**1417**	… waren Sie für Ihre Freunde oder Verwandten da, wenn diese Sie gebraucht haben?	1	2	3	4	5

DEMQoL (Demenz)

Quelle: Smith, S. C., Lamping, D. L., Banerjee, S., Harwood, R., Foley, B., Smith, P., ... & Mann, A. (2005). Measurement of health-related quality of life for people with dementia: development of a new instrument (DEMQOL) and an evaluation of current methodology. Health Technology Assessment (Winchester, England), 9(10), 1-93.

Berwig, M., Leicht, H., & Gertz, H. J. (2009). Critical evaluation of self-rated quality of life in mild cognitive impairment and Alzheimer's disease — Further evidence for the impact of anosognosia and global cognitive impairment. JNHA - The Journal of Nutrition, Health and Aging, 13(3), 226–230. doi:10.1007/s12603-009-0063-4

Berwig, M., Leicht, H., Hartwig, K., & Gertz, H. J. (2011). Self-rated quality of life in mild cognitive impairment and Alzheimer's disease: The problem of affective distortion. GeroPsych: The Journal of Gerontopsychology and Geriatric Psychiatry, 24(1), 45–51. doi:http://dx.doi.org/10.1024/1662-9647/a000029

Lizenzfrei, deutsche Version von Brewig erhalten; Item nach genereller LQ gestrichen, da schon in WHOQoL

PROG: Indikation = 3 (Demenz)

PROG: Indikation = 3

Im Folgenden möchten wir Sie zu Beschwerden befragen, die häufig im Alter auftreten. Beispiele sind Gedächtnisstörungen, Schwierigkeiten im alltäglichen Leben oder eine gedrückte Stimmung. Wir möchten Sie auch fragen, wie häufig die Beschwerden waren und wie besorgt Sie über die Beschwerden waren. Es gibt keine richtigen und keine falschen Antworten.

	**Zunächst möchten wir Sie zu Ihren Gefühlen fragen. Wie oft haben Sie die folgenden Gefühle in den letzten 2 Wochen erlebt?**	nie	selten	manchmal	häufig
**3401**	Heiterkeit	1	2	3	4
**3402**	Angst oder Sorge	1	2	3	4
**3403**	Haben Sie das Leben genossen?	1	2	3	4
**3404**	Frustration	1	2	3	4
**3405**	Selbstsicherheit	1	2	3	4
**3406**	Voller Energie	1	2	3	4
**3407**	Traurigkeit	1	2	3	4
**3408**	Einsamkeit	1	2	3	4
**3409**	Verzweiflung	1	2	3	4
**3410**	Lebhaftigkeit	1	2	3	4
**3411**	Gereiztheit	1	2	3	4
**3412**	Überdruss	1	2	3	4

3413	Unfähigkeit	1	2	3	4
	**Jetzt folgen Fragen zu Ihrem Gedächtnis. Wie oft waren Sie in den letzten 2 Wochen besorgt wegen der folgenden Beschwerden?**	1	2	3	4
3414	Besorgt, weil Sie kurz zurückliegende Ereignisse vergessen	1	2	3	4
3415	Besorgt, weil Sie vergessen, wer bestimmte Personen sind	1	2	3	4
3416	Besorgt, weil Sie vergessen, welcher Wochentag ist	1	2	3	4
3417	Besorgt, weil Ihre Gedanken durcheinander sind	1	2	3	4
3418	Besorgt, weil Sie keine Entscheidungen treffen können	1	2	3	4
3419	Besorgt wegen Konzentrationsstörungen				
	**Jetzt folgen Fragen über Ihren Alltag. Wie oft waren Sie in den letzten 2 Wochen besorgt über folgende Lebensumstände?**	1	2	3	4
3420	Besorgt, weil Sie nicht genug Gesellschaft hatten	1	2	3	4
3421	Besorgt, weil Sie nicht wussten, wie Sie mit anderen Menschen in Ihrer Umgebung umgehen sollen	1	2	3	4
3422	Besorgt, weil Sie nicht die Zuneigung bekamen, die Sie sich gewünscht haben	1	2	3	4
3423	Besorgt, weil Ihnen nicht zugehört wurde	1	2	3	4
3424	Besorgt, weil Sie sich nicht verständlich machen konnten	1	2	3	4
3425	Besorgt, weil Sie nicht die Hilfe bekommen haben, die Sie gebraucht hätten	1	2	3	4
3426	Besorgt, es nicht rechtzeitig auf die Toilette zu schaffen	1	2	3	4
3427	Besorgt, weil Sie sich in ihrer eigenen Haut nicht wohl fühlten	1	2	3	4
3428	Besorgt über Ihren allgemeinen Gesundheitszustand	1	2	3	4

MSIS

Quelle: Hobart, J., Lamping, D., Fitzpatrick, R., Riazi, A., Thompson, A. (2001). The Multiple Sclerosis Impact Scale (MSIS-29): A new patient-based outcome measure. Brain, 124, 962-973.
Schönberg, P. (2012). Validierung der deutschen Version der Multiple Sclerosis Impact Scale (MSIS-29). Dissertation. Universitätsklinikum Hamburg-Eppendorf

lizenzfrei

PROG: Indikation = 5 (Multiple Sklerose)

PROG: Indikation = 5

In den folgenden Fragen geht es um den Einfluss Ihrer Multiple Sklerose Erkrankung auf Ihr tägliches Leben.

Bitte kreuzen Sie für jede Aussage an, was am besten auf Ihre Situation zutrifft.

	**Wie schwer fiel es Ihnen in den letzten zwei Wochen …**	gar nicht	ein bissche n	mäßig	ziemlich	sehr
**5401**	körperlich anstrengende Dinge zu tun?	1	2	3	4	5
**5402**	Dinge fest anzufassen (z. B. Hahn aufdrehen)?	1	2	3	4	5
**5403**	Dinge zu tragen?	1	2	3	4	5
	**Hatten Sie in den letzten zwei Wochen…**					
**5404**	Probleme mit dem Gleichgewicht?	1	2	3	4	5
**5405**	Schwierigkeiten, sich in der Wohnung zu bewegen?	1	2	3	4	5
**5406**	das Gefühl ungeschickt zu sein?	1	2	3	4	5
**5407**	ein Steifigkeitsgefühl?	1	2	3	4	5
**5408**	schwere Arme und / oder Beine?	1	2	3	4	5
**5409**	Zittern der Arme oder Beine?	1	2	3	4	5
**5410**	Krämpfe der Extremitäten?	1	2	3	4	5
**5411**	das Gefühl, dass ihr Körper nicht tat, was sie wollten?	1	2	3	4	5
**5412**	Beeinträchtigung im sozialen und Freizeitleben zu Hause?	1	2	3	4	5
**5413**	Probleme mit den Händen bei Alltagstätigkeiten?	1	2	3	4	5
**5414**	Probleme sich fortzubewegen (Auto, Bus, Taxi, Zug)?	1	2	3	4	5
**5415**	länger gebraucht, Dinge zu tun?	1	2	3	4	5
**5416**	Schwierigkeiten, Dinge spontan zu machen?	1	2	3	4	5

5417	das Gefühl, ganz schnell zur Toilette zu müssen?	1	2	3	4	5
5418	sich allgemein unwohl gefühlt?	1	2	3	4	5
5419	Schlafprobleme?	1	2	3	4	5
5420	sich geistig / mental müde gefühlt?	1	2	3	4	5
5421	Sorgen bezogen auf ihre MS?	1	2	3	4	5
5422	sich angespannt und ängstlich gefühlt?	1	2	3	4	5
5423	sich ungeduldig und aufbrausend gefühlt?	1	2	3	4	5
5424	Konzentrationsprobleme?	1	2	3	4	5
5425	keine Zuversicht?	1	2	3	4	5
5426	sich traurig / depressiv gefühlt?	1	2	3	4	5
	**Waren Sie in den letzten zwei Wochen…**					
5427	davon abhängig, dass andere Dinge für sie erledigten?	1	2	3	4	5
5428	gezwungen, zu Hause zu bleiben?	1	2	3	4	5
5429	Gezwungen, die Zeit für Arbeit oder Alltagsaktivitäten einzuschränken?	1	2	3	4	5

PROG: Auf jeder Seite mit diesen Items:

®2000 Neurological Outcome Measures Unit

PDQ-39 (Parkinson)

Quelle: Berger, K., Broll, S., Winkelmann, J., Heberlein, I., Müller, T., Ries, V. für die FAQT-Studienzentren (1999). Untersuchung zur Reliabilität der deutschen Version des PDQ-39: Ein krankheitsspezifischer Fragebogen zur Erfassung der Lebensqualität von Parkinson-Patienten. Aktuelle Neurologie, 26, 180-184.

lizenzfrei

PROG: Indikation = 6 (Parkinson)

PROG: Indikation = 6

In den folgenden Fragen geht es darum, wie es Ihnen in verschiedenen Bereichen in Ihrem Leben ergangen ist.

	**Wie oft haben Sie in den letzten 2 Wochen wegen Ihrer Parkinsonerkrankung…**	niemals	selten	manchmal	häufig	Immer oder kann ich überhaupt nicht
**6401**	… Schwierigkeiten gehabt, Freizeitaktivitäten, die Sie gern machen würden, auszuüben?	1	2	3	4	5
**6402**	… Schwierigkeiten gehabt, Ihren Haushalt zu versorgen (z. B. handwerkliche Tätigkeiten, Hausarbeiten, Kochen)?	1	2	3	4	5
**6403**	… Schwierigkeiten gehabt, Einkaufstaschen zu tragen?	1	2	3	4	5
**6404**	… Probleme gehabt, ungefähr 1 km zu gehen?	1	2	3	4	5
**6405**	… Probleme gehabt, ungefähr 100 m zu gehen?	1	2	3	4	5
**6406**	… Probleme gehabt, sich im Haus so zu bewegen, wie Sie wollten?	1	2	3	4	5
**6407**	… Probleme gehabt, sich in der Öffentlichkeit zu bewegen?	1	2	3	4	5
**6408**	… eine Begleitperson gebraucht, um sich außer Haus zu bewegen?	1	2	3	4	5
**6409**	… Angst oder Sorgen gehabt, dass Sie in der Öffentlichkeit hinfallen?	1	2	3	4	5
**6410**	… das Gefühl gehabt, mehr an das Haus gebunden zu sein, als Ihnen lieb wäre?	1	2	3	4	5
**6411**	… Schwierigkeiten gehabt, sich selbst zu waschen?	1	2	3	4	5
**6412**	… Schwierigkeiten gehabt, sich selbst anzuziehen?	1	2	3	4	5
**6413**	… Probleme gehabt, Knöpfe zu schließen oder Schnürsenkel zu binden?	1	2	3	4	5
**6414**	… Probleme gehabt, deutlich zu schreiben?	1	2	3	4	5
**6415**	… Schwierigkeiten gehabt, Ihr Essen klein zu schneiden?	1	2	3	4	5
**6416**	… Schwierigkeiten gehabt, ein Getränk zu halten, ohne es zu verschütten?	1	2	3	4	5

6417	… sich niedergeschlagen oder deprimiert gefühlt?	1	2	3	4	5
6418	… sich isoliert oder einsam gefühlt?	1	2	3	4	5
6419	… sich verärgert oder verbittert gefühlt?	1	2	3	4	5
6420	… sich den Tränen nahe gefühlt?	1	2	3	4	5
6421	… sich ängstlich gefühlt?	1	2	3	4	5
6422	… sich Sorgen über Ihre Zukunft gemacht?	1	2	3	4	5
6423	… das Gefühl gehabt, Ihre Parkinsonerkrankung vor anderen verheimlichen zu müssen?	1	2	3	4	5
6424	… Situationen vermieden, die mit Essen oder Trinken in der Öffentlichkeit verbunden waren?	1	2	3	4	5
6425	… sich in der Öffentlichkeit wegen Ihrer Parkinsonerkrankung geschämt?	1	2	3	4	5
6426	… sich Sorgen über die Reaktionen anderer Ihnen gegenüber gemacht?	1	2	3	4	5
6427	… Probleme im Verhältnis mit Ihnen nahe stehenden Menschen gehabt?	1	2	3	4	5
6428	… nicht die Unterstützung erhalten, die Sie von Ihrem (Ehe-)Partner benötigt hätten?	1	2	3	4	5
6429	… nicht die Unterstützung erhalten, die Sie von Ihren Verwandten oder engen Freunden benötigt hätten?	1	2	3	4	5
6430	… das Problem gehabt, tagsüber unerwartet einzuschlafen?	1	2	3	4	5
6431	… Probleme gehabt, sich zu konzentrieren (z. B. beim Lesen oder beim Fernsehen)?	1	2	3	4	5
6432	… das Gefühl gehabt, dass Sie ein schlechtes Gedächtnis hätten?	1	2	3	4	5
6433	… schlechte Träume oder Halluzinationen gehabt?	1	2	3	4	5
6434	… Schwierigkeiten mit dem Sprechen gehabt?	1	2	3	4	5
6435	… sich außer Stande gefühlt, mit anderen zu kommunizieren?	1	2	3	4	5
6436	… den Eindruck gehabt, von anderen nicht beachtet zu werden?	1	2	3	4	5
6437	… schmerzhafte Muskelkrämpfe gehabt?	1	2	3	4	5
6438	… Schmerzen in den Gelenken oder anderen Körperteilen gehabt?	1	2	3	4	5
6439	… sich unangenehm heiß oder kalt gefühlt?	1	2	3	4	5

SA-SIP30 (Schlaganfall)

Quelle: van Straten, A., de Haan, R. J., Limburg, M., Schuling, J., Bossuyt, P. M., van de Bos, G. A. M. (1997). A Stroke-Adapted 30-Item Version of the Sickness Impact Profile to Assess Quality of Life (SA-SIP30). Stroke, 28, 2155-2161.

Hütter, B. O. (2002). Sickness Impact Profile (SIP) -German version. In S. Salek (Ed.), Compendium of qua/ity of /ife instruments. Chichester, West Sussex: Wiley.

Lizenzfrei – deutsche Version von Hütter bekommen

PROG: Indikation = 7 (Schlaganfall)

PROG: Indikation = 7

Dieser Fragebogen dient dazu festzustellen, welche Beschwerden Sie im Augenblick haben.

Bitte lesen Sie sich alle Sätze genau durch und antworten Sie nur bei solchen Beschwerden mit "ja", die Sie am heutigen Tag haben und die sich auf Ihren Gesundheitszustand beziehen.

		ja	nein
**7401**	Ich kann schwierige Bewegungen nur mit Hilfe machen, wie z. B. in ein Auto oder eine Badewanne ein- und aussteigen.	1	0
**7402**	Meine Hände oder Finger kann ich nur mit Einschränkungen oder Schwierigkeiten gebrauchen.	1	0
**7403**	Wenn ich ins Bett gehen will oder aufstehen möchte, z. B. von einem Stuhl, so muss ich mich an etwas festhalten oder einen Stock benutzen.	1	0
**7404**	Ich habe Schwierigkeiten, mir alleine Schuhe, Strümpfe oder Socken anzuziehen.	1	0
**7405**	Ich kann mich nur anziehen, wenn mir jemand hilft.	1	0
**7406**	Ich kann mich für die Probleme von anderen nicht mehr so interessieren, z. B. höre ich nicht zu, wenn sie mir von ihren Problemen erzählen, oder ich biete ihnen keine Hilfe an.	1	0
**7407**	Ich bin oft ungehalten zu denjenigen, die mich umgeben, z. B. schneide ich ihnen das Wort ab, gebe scharfe Antworten oder kritisiere leicht.	1	0
**7408**	Ich zeige weniger Zuneigung.	1	0
**7409**	Ich unternehme weniger soziale Aktivitäten mit Gruppen von Leuten.	1	0
**7410**	Ich spreche weniger mit denjenigen, die mich umgeben.	1	0
**7411**	Ich bleibe die meiste Zeit zu Hause.	1	0
**7412**	Ich gehe nicht in die Stadt.	1	0
**7413**	Ich bewege mich ohne fremde Hilfe nicht in der Dunkelheit oder in unbeleuchteten Plätzen.	1	0

**7414**	Ich nehme nur noch dann an einem Gespräch teil, wenn ich der anderen Person sehr nahe stehe oder zu ihr schaue.	1	0
**7415**	Ich habe Schwierigkeiten in der Aussprache, z. B. stottere ich, bleibe stecken, stammle oder kann die Worte nicht mehr deutlich aussprechen.	1	0
**7416**	Ich kann nicht klar sprechen, wenn ich unter Stress bin.	1	0
**7417**	Ich sage, wie schlecht oder nutzlos ich bin, z. B. dass ich für andere eine Last bin.	1	0
**7418**	Ich lache oder weine plötzlich.	1	0
**7419**	Ich bin gereizt und ungeduldig mit mir selbst, z. B. spreche ich schlecht über mich, verfluche mich, gebe mir die Schuld, wenn etwas passiert.	1	0
**7420**	Ich bekomme plötzliche Angstzustände.	1	0
**7421**	Ich kann die normale Hausarbeit, die ich eigentlich tun müsste, überhaupt nicht mehr tun.	1	0
**7422**	Ich gehe nicht mehr einkaufen, so wie ich es normalerweise tun würde.	1	0
**7423**	Ich kann nicht mehr die Wohnung oder das Haus putzen, so wie ich es normalerweise tun würde.	1	0
**7424**	Ich kann die Wäsche nicht mehr waschen, so wie ich es normalerweise tun würde.	1	0
**7425**	Ich bin durcheinander und fange mehrere Sachen gleichzeitig an.	1	0
**7426**	Ich mache mehr Fehler als üblich.	1	0
**7427**	Ich habe Schwierigkeiten, Tätigkeiten auszuführen, die Konzentration und Denken erfordern.	1	0
**7428**	Ich gehe keine Steigungen mehr und / oder vermeide abschüssige Strecken.	1	0
**7429**	Ich kann mich nur noch mit Hilfe eines Spazierstocks, Krücken, Wänden oder Möbeln fortbewegen.	1	0
**7430**	Ich laufe langsamer.	1	0

5. IG: Versorgungsqualität & Inanspruchnahme und Bewertung projektspezifischer Leistungen

Seitenüberschrift: Versorgung in NPPV

Quelle: eigen

PROG: Alle Fragen unter 5. nur für IG
Gruppe = 1 (IG)

PROG: Extra Seite mit folgendem Text, nicht fett und größere Schrift

PROG: Gruppe = 1 & Indikation = 1 | 2 | 4
Sie nehmen seit einiger Zeit aufgrund Ihrer seelischen Erkrankung an dem neuen Versorgungsmodell (NPPV) teil. In Ihrer Behandlung wurde Ihnen ein Bezugstherapeut bzw. eine Bezugstherapeutin zur Seite gestellt. Die folgenden Fragen beziehen sich auf die Behandlung bei Ihrem Bezugstherapeuten oder Ihrer Bezugstherapeutin.

Auf den folgenden Seiten möchten wir gern mehr darüber erfahren, wie Sie die Behandlung in dieser neuen Versorgungsform erleben und wie Sie die Qualität der Behandlung einschätzen.
Bitte denken Sie daran, falls Sie den Fragebogen stellvertretend für jemanden ausfüllen, alle Fragen aus Sicht der erkrankten Person zu beantworten.

PROG: Gruppe = 1 & Indikation = 3 | 5 | 6 | 7
Sie nehmen seit einiger Zeit aufgrund Ihrer Erkrankung des Nervensystems an dem neuen Versorgungsmodell (NPPV) teil. In Ihrer Behandlung wurde Ihnen ein Bezugsarzt bzw. eine Bezugsärztin zur Seite gestellt. Die folgenden Fragen beziehen sich auf die Behandlung bei Ihrem Bezugsarzt oder Ihrer Bezugsärztin.

Auf den folgenden Seiten möchten wir gern mehr darüber erfahren, wie Sie die Behandlung in dieser neuen Versorgungsform erleben und wie Sie die Qualität der Behandlung einschätzen.
Bitte denken Sie daran, falls Sie den Fragebogen stellvertretend für jemanden ausfüllen, alle Fragen aus Sicht der erkrankten Person zu beantworten.

PROG: Gruppe = 1 (für alle Fragen unter 5.; bis Frage 515_3)

PROG: Indikation = 1 | 2 | 4 & Text immer bis Frage 509_1 einblenden

Sie nehmen seit einiger Zeit aufgrund Ihrer seelischen Erkrankung an dem neuen Versorgungsmodell (NPPV) teil. Die folgenden Fragen beziehen sich auf Ihre Erfahrungen in den letzten drei Monaten mit dieser Behandlungsform, das heißt die Monate [Monate].

PROG: Indikation = 3 | 5 | 6 | 7 & Text immer bis Frage 509_1 einblenden

Sie nehmen seit einiger Zeit aufgrund Ihrer Erkrankung des Nervensystems an dem neuen Versorgungsmodell (NPPV) teil. Die folgenden Fragen beziehen sich auf Ihre Erfahrungen in den letzten drei Monaten mit dieser Behandlungsform, das heißt die Monate [Monate].

PROG: alle Indikationen

Im Folgenden ist mit „Arzt / (Psycho-)Therapeut / Neurologe / Psychiater“ immer auch die weibliche Form „Ärztin / (Psycho-)Therapeutin / Neurologin / Psychiaterin“ eingeschlossen.

509n (T0_n.a.) PROG: Indikation = 1 | 2 | 4

Nehmen Sie für Ihre Behandlung in regelmäßigen Abständen Termine bei Ihrem Bezugsarzt / Bezugspsychotherapeuten wahr?

PROG: Indikation = 3 | 5 | 6 | 7

Nehmen Sie für Ihre Behandlung in regelmäßigen Abständen Termine bei Ihrem Bezugsarzt wahr?

❑ ja (1) ❑ nein (0)

509_1n (T0_n.a.) PROG: Frage 509 = 1 (ja)

Wie häufig nehmen Sie regelmäßige Behandlungstermine wahr?

jede Woche	alle 2 Wochen	jeden Monat	alle 2-3 Monate	alle 4-6 Monate	seltener
1	2	3	4	5	6

511n PROG: Indikation = 1 | 2 | 4

Haben Sie in den letzten 3 Monaten Ihrer Behandlung Ihren Bezugsarzt / Bezugspsychotherapeuten gewechselt?

PROG: Indikation = 3 | 5 | 6 | 7

Haben Sie in den letzten 3 Monaten Ihrer Behandlung Ihren Bezugsarzt gewechselt?

❑ ja (1) ❑ nein (0)

511_1 PROG: Frage 511 = 1 (ja)

nämlich _____________ Mal (numerisch, dreistellig, > 0)

PROG: Indikation = 1 | 2 | 4; auf jeder Seite bis Frage 503 anzeigen

Bitte beziehen Sie die folgenden Fragen auf Ihren Bezugsarzt / Bezugspsychotherapeuten.

PROG: Indikation = 3 | 5 | 6 | 7; auf jeder Seite bis Frage 503 anzeigen

Bitte beziehen Sie die folgenden Fragen auf Ihren Bezugsarzt.

PROG: kein Filter

Wie schätzen Sie die Qualität der Behandlung in den letzten 3 Monaten ein?

501n (T0_n.a.) PROG: Indikation = 1 | 2 | 4

In meiner Behandlung nimmt sich mein Arzt / Psychotherapeut immer genug Zeit für mich.

PROG: Indikation = 3 | 5 | 6 | 7

In meiner Behandlung nimmt sich mein Arzt immer genug Zeit für mich.

stimmt überhaupt nicht	stimmt nicht	weder noch	stimmt	stimmt voll und ganz
1	2	3	4	5

502n (T0_n.a.)

PROG: kein Filter

In meiner Behandlung fühle ich mich sehr gut betreut.

stimmt überhaupt nicht	stimmt nicht	weder noch	stimmt	stimmt voll und ganz
1	2	3	4	5

503n (T0_n.a.)

Meine Behandlung deckt meine Bedürfnisse voll und ganz ab.

stimmt überhaupt nicht	stimmt nicht	weder noch	stimmt	stimmt voll und ganz
1	2	3	4	5

Teil des neuen Versorgungsmodells in NPPV sind verschiedene Angebote und die Möglichkeit an Gruppen oder Online-Selbsthilfe (Novego) zur Unterstützung der Behandlung teilzunehmen. Wir möchten nun von Ihnen wissen, wie Sie die Behandlung im neuen Versorgungsmodell bewerten und welche Angebote Sie wahrnehmen.

600_3

PROG: Indikation = 1 | 2 | 4

In meiner Behandlung in NPPV hat mir mein Arzt / Psychotherapeut sinnvolle therapeutische Angebote empfohlen.

PROG: Indikation = 3 | 5 | 6 | 7

In meiner Behandlung in NPPV hat mir mein Arzt sinnvolle therapeutische Angebote empfohlen.

stimmt überhaupt nicht	stimmt nicht	weder noch	stimmt	stimmt voll und ganz
1	2	3	4	5

600_4

PROG: Indikation = 1 | 2 | 4

In meiner Behandlung in NPPV hat mir mein Arzt / Psychotherapeut ausführlich erklärt, was ich bei Notfällen und Krisen, zum Beispiel, wenn es mir plötzlich schlechter geht, tun soll.

PROG: Indikation = 3 | 5 | 6 | 7

In meiner Behandlung in NPPV hat mir mein Arzt ausführlich erklärt, was ich bei Notfällen und Krisen, zum Beispiel wenn es mir plötzlich schlechter geht, tun soll.

stimmt überhaupt nicht	stimmt nicht	weder noch	stimmt	stimmt voll und ganz
1	2	3	4	5

600_5

PROG: Indikation = 1 | 2 | 4

In meiner Behandlung in NPPV hat mich mein Arzt / Psychotherapeut ausführlich informiert, dass ich in Notfällen und Krisen schnell einen Termin bei ihm bekomme.

PROG: Indikation = 3 | 5 | 6 | 7

In meiner Behandlung in NPPV hat mich mein Arzt ausführlich informiert, dass ich in Notfällen und Krisen schnell einen Termin bei ihm bekomme.

stimmt überhaupt nicht	stimmt nicht	weder noch	stimmt	stimmt voll und ganz
1	2	3	4	5

504n (T0_n.a.)

PROG: Indikation = 1 | 2 | 4

In meiner Behandlung in NPPV erklärte mir mein Arzt / Psychotherapeut meinen Therapieverlauf einfach und verständlich.

PROG: Indikation = 3 | 5 | 6 | 7

In meiner Behandlung in NPPV erklärte mir mein Arzt meinen Therapieverlauf einfach und verständlich.

stimmt überhaupt nicht	stimmt nicht	weder noch	stimmt	stimmt voll und ganz
1	2	3	4	5

510n (T0_n.a.)

PROG: Indikation = 1 | 2 | 4

In meiner Behandlung in NPPV arbeiten verschiedene Ärzte / Psychotherapeuten gut zusammen und informieren sich gegenseitig.

PROG: Indikation = 3 | 5 | 6 | 7

In meiner Behandlung in NPPV arbeiten verschiedene Ärzte gut zusammen und informieren sich gegenseitig.

stimmt überhaupt nicht	stimmt nicht	weder noch	stimmt	stimmt voll und ganz
1	2	3	4	5

510_1n (T0_n.a.)

PROG: Indikation = 1 | 2 | 4

In meiner Behandlung in NPPV vermittelt mich mein Arzt / Psychotherapeut bei Bedarf an kompetente Stellen weiter (z. B. Physio- / Ergotherapie, anderer Facharzt).

PROG: Indikation = 3 | 5 | 6 | 7

In meiner Behandlung in NPPV vermittelt mich mein Arzt bei Bedarf an kompetente Stellen weiter (z.B. Physio- / Ergotherapie, anderer Facharzt).

stimmt überhaupt nicht	stimmt nicht	weder noch	stimmt	stimmt voll und ganz
1	2	3	4	5

Gruppenangebote

601 PROG: Indikation = 1 | 2 | 4

Wurde Ihnen von Ihrem Arzt / Psychotherapeuten ein Gruppenangebot empfohlen?

PROG: Indikation = 3 | 5 | 6 | 7

Wurde Ihnen von Ihrem Arzt ein Gruppenangebot empfohlen?

Ja (1) / nein (0)

Wie wurden Sie über die Gruppenangebote informiert?

601_1 PROG: Frage 601 = 1 (ja) & Indikation = 1 | 2 | 4

Mein Arzt / Psychotherapeut hat mir ausführlich den Inhalt und Ablauf des Gruppenangebots beschrieben.

PROG: Frage 601 = 1 (ja) & Indikation = 3 | 5 | 6 | 7

Mein Arzt hat mir ausführlich den Inhalt und Ablauf des Gruppenangebots beschrieben.

stimmt überhaupt nicht	stimmt nicht	weder noch	stimmt	stimmt voll und ganz
1	2	3	4	5

601_2 PROG: Frage 601 = 1 (ja) & Indikation = 1 | 2 | 4

Mein Arzt / Psychotherapeut hat mir genau erläutert, warum das Gruppenangebot für mich hilfreich ist.

PROG: Frage 601 = 1 (ja) & Indikation = 3 | 5 | 6 | 7

Mein Arzt hat mir genau erläutert, warum das Gruppenangebot für mich hilfreich ist.

stimmt überhaupt nicht	stimmt nicht	weder noch	stimmt	stimmt voll und ganz
1	2	3	4	5

PROG: Frage 601 = 1 (ja)

Welche Gruppenangebote wurden Ihnen empfohlen und haben Sie diese bereits wahrgenommen?

PROG: Mehrfachantwort möglich

	Gruppenangebot	empfohlen	wahrgenommen
**601_31**<br>**601_32**	Gruppe für Betroffene	ja (1) / nein (0)	ja (1) / nein (0)
**601_41**<br>**601_42**	Gruppe für Angehörige	ja (1) / nein (0)	ja (1) / nein (0)

601_10 PROG: Frage 601 = 1 (ja) & mind. einmal (Frage 601_x1 = 1 (ja) & Frage 601_x2 = 0 (nein))

Sie haben angegeben, dass Sie mindestens an einem Angebot, das Ihnen empfohlen wurde, noch nicht teilgenommen haben. Haben Sie noch vor das Angebot wahrzunehmen?

ja (1) / nein (0)

PROG: PROG: Frage 601 = 1 (ja) & Frage 601_10 = 0 (nein)

Welche Gründe haben Sie dafür?

PROG: Mehrfachantwort möglich

❑ Ich habe kein Interesse daran. (1 genannt; 0 nicht genannt)

❑ Der Aufwand ist mir zu groß. (1 genannt; 0 nicht genannt)

❑ Der Weg ist mir zu weit. (1 genannt; 0 nicht genannt)

❑ Ich glaube nicht, dass mir das Angebot hilft. (1 genannt; 0 nicht genannt)

❑ Ich möchte meine Probleme nicht vor anderen Patienten besprechen. (1 genannt; 0 nicht genannt)

❑ anderer Grund (1 genannt; 0 nicht genannt)

PROG: Frage 601_13 = 1 (anderer Grund)

Und zwar: _____________

Sie haben an mindestens einem Gruppenangebot teilgenommen. Bitte geben Sie an, inwiefern Sie den Aussagen zustimmen.

601_5 PROG: Frage 601 = 1 (ja) & mind. einmal (Frage 601_x2 = 1 (ja))

Das Gruppenangebot hat mir sehr geholfen, im Alltag mit meiner Erkrankung zurecht zu kommen.

stimmt überhaupt nicht	stimmt nicht	weder noch	stimmt	stimmt voll und ganz
1	2	3	4	5

601_6 PROG: Frage 601 = 1 (ja) & mind. einmal (Frage 601_x2 = 1 (ja))

Durch das Gruppenangebot belastet mich meine Erkrankung deutlich weniger.

stimmt überhaupt nicht	stimmt nicht	weder noch	stimmt	stimmt voll und ganz
1	2	3	4	5

601_7 PROG: Frage 601 = 1 (ja) & mind. einmal (Frage 601_x2 = 1 (ja))

Durch das Gruppenangebot habe ich meine Erkrankung besser verstanden.

stimmt überhaupt nicht	stimmt nicht	weder noch	stimmt	stimmt voll und ganz
1	2	3	4	5

601_8 PROG: Frage 601 = 1 (ja) & mind. einmal (Frage 601_x2 = 1 (ja))

Der Austausch mit anderen Betroffenen beim Gruppenangebot hat mir sehr geholfen.

stimmt überhaupt nicht	stimmt nicht	weder noch	stimmt	stimmt voll und ganz

1	2	3	4	5

Online-Selbsthilfe

602 PROG: Indikation = 1 | 2 | 4

Wurde Ihnen von Ihrem Arzt / Psychotherapeuten eine Online-Selbsthilfe (Novego) empfohlen?

PROG: Indikation = 3 | 5 | 6 | 7

Wurde Ihnen von Ihrem Arzt eine Online-Selbsthilfe (Novego) empfohlen?

Ja (1) / nein (0)

Wie wurden Sie über die Online-Selbsthilfe informiert?

602_1 PROG: Frage 602 = 1 (ja) & Indikation = 1 | 2 | 4

Mein Arzt / Psychotherapeut hat mir ausführlich den Inhalt und Ablauf der Online-Selbsthilfe (Novego) beschrieben.

PROG: Frage 602 = 1 (ja) & Indikation = 3 | 5 | 6 | 7

Mein Arzt hat mir ausführlich den Inhalt und Ablauf des Online-Selbsthilfe (Novego) beschrieben.

stimmt überhaupt nicht	stimmt nicht	weder noch	stimmt	stimmt voll und ganz
1	2	3	4	5

602_2 PROG: Frage 602 = 1 (ja) & Indikation = 1 | 2 | 4

Mein Arzt / Psychotherapeut hat mir genau erläutert, warum die Online-Selbsthilfe (Novego) für mich hilfreich ist.

PROG: Frage 602 = 1 (ja) & Indikation = 3 | 5 | 6 | 7

Mein Arzt hat mir genau erläutert, warum die Online-Selbsthilfe (Novego) für mich hilfreich ist.

stimmt überhaupt nicht	stimmt nicht	weder noch	stimmt	stimmt voll und ganz
1	2	3	4	5

602_3 PROG: Frage 602 = 1 (ja)

Haben Sie das Angebot der Online-Selbsthilfe (Novego) bereits wahrgenommen?

- ❑ ja (1)
- ❑ nein, aber ich habe vor es wahrzunehmen (2)
- ❑ nein und ich habe nicht vor es wahrzunehmen (0)

602_3 1 PROG: Frage 602 = 1 (ja) & Frage 602_3 = 0 (nein)

Sie haben angegeben, dass Sie an der Online-Selbsthilfe (Novego), die Ihnen empfohlen wurde, nicht teilnehmen wollen.

Welche Gründe haben Sie dafür?

PROG: Mehrfachantwort möglich

❑ Ich habe kein Interesse daran. (1 genannt; 0 nicht genannt)

❑ Der Aufwand ist mir zu groß. (1 genannt; 0 nicht genannt)

❑ Ich glaube nicht, dass mir die Online-Selbsthilfe (Novego) hilft. (1 genannt; 0 nicht genannt)

❑ anderer Grund (1 genannt; 0 nicht genannt)

Und zwar: ____________

PROG: Frage 602 = 1 (ja) & Frage 602_3 = 1 (ja)

Sie haben bereits an einer Online-Selbsthilfe (Novego) teilgenommen. Bitte geben Sie an, inwiefern Sie den folgenden Aussagen zustimmen.

602_4 PROG: Frage 602 = 1 (ja) & Frage 602_3 = 1 (ja)

Die Online-Selbsthilfe half mir sehr, im Alltag mit meiner Erkrankung zurecht zu kommen.

stimmt überhaupt nicht	stimmt nicht	weder noch	stimmt	stimmt voll und ganz
1	2	3	4	5

602_5 PROG: Frage 602 = 1 (ja) & Frage 602_3 = 1 (ja)

Durch die Online-Selbsthilfe belastet mich meine Erkrankung deutlich weniger.

stimmt überhaupt nicht	stimmt nicht	weder noch	stimmt	stimmt voll und ganz
1	2	3	4	5

602_6 PROG: Frage 602 = 1 (ja) & Frage 602_3 = 1 (ja)

Durch die Online-Selbsthilfe verstehe ich meine Erkrankung besser.

stimmt überhaupt nicht	stimmt nicht	weder noch	stimmt	stimmt voll und ganz
1	2	3	4	5

Schübe
Quelle: eigen
PROG: Indikation =5

516n (T0_n. a.) PROG: Indikation = 5

Haben Sie in den letzten drei Monaten, das heißt [Monate], einen Schub erlebt?

❑ ja (1) ❑ nein (0)

**516_1n (T0_n.a.)**	PROG: Indikation = 5 & Frage 516 = 1 (Schub erlebt)<br>**Haben Sie in dieser Zeit eine Schubtherapie gemacht?**<br>❑ ja, ambulant bei meinem behandelnden Arzt (1)<br>❑ ja, in einem Krankenhaus (2)<br>❑ nein (0)

Entlassungsmanagement
Quelle: eigen

**512n (T0_n.a.)**	PROG: Indikation = 1 | 2 | 4<br>**Wie viele Nächte waren Sie <u>in den vergangenen 3 Monaten, d.h. in den Monaten [Monate]</u> aufgrund Ihrer seelischen Erkrankung zur stationären Behandlung in einem Krankenhaus?**<br>PROG: Indikation = 3 | 5 | 6 | 7<br>**Wie viele Nächte waren Sie <u>in den vergangenen 3 Monaten, d.h. in den Monaten [Monate]</u> aufgrund Ihrer Erkrankung des Nervensystems zur stationären Behandlung in einem Krankenhaus?**<br>____ Nächte (numerisch, 3-stellig, max: 120)
**512_1n (T0_n.a.)**	PROG: Frage 512 > 0 & Indikation = alle<br>**Versuchen Sie sich an die letzte Entlassung aus dem Krankenhaus zu erinnern.**<br>PROG: Indikation = 1 | 2 | 4<br>**Wie viel Zeit ist zwischen Ihrer Entlassung und Ihrem ersten Termin bei Ihrem niedergelassenen Arzt / Psychotherapeuten verstrichen?**<br>PROG: Indikation = 3 | 5 | 6 | 7<br>**Wie viel Zeit ist zwischen Ihrer Entlassung und Ihrem ersten Termin bei Ihrem niedergelassenen Arzt verstrichen?**

bis zu 7 Tage	1 bis 2 Wochen	3 bis 4 Wochen	1 bis 3 Monate	länger als 3 Monate
1	2	3	4	5

Behandlungsabbrüche

Quelle: eigen

PROG: teilweise nach Indikation gefiltert

PROG: kein Filter

Manchmal hat man das Gefühl, dass eine Behandlung nicht so gut hilft oder einem beispielsweise aufgrund von Nebenwirkungen nicht guttut. Manche Patienten brechen deshalb eine Therapie <u>gegen den ärztlichen Rat</u> ab. Zum Beispiel gehen Sie zu vorgesehenen Folgeterminen nicht mehr hin oder nehmen die Medikamente nicht mehr wie vereinbart.

513n (T0_n.a.) PROG: Indikation = 1 | 2 | 4

Haben Sie in den letzten 3 Monaten eine Behandlung Ihrer seelischen Erkrankung gegen ärztlichen Rat abgebrochen?

PROG: Indikation = 3 | 5 | 6 | 7

Haben Sie in den letzten 3 Monaten eine Behandlung Ihrer Erkrankung des Nervensystems gegen ärztlichen Rat abgebrochen?

❑… ja (1) ❑… nein (0)

Fragen zu Krisensituationen
Quelle: eigen

PROG: teilweise nach Indikation gefiltert

PROG: Indikation = 1 | 2 | 4, letzte drei Monate angeben

Manchmal gibt es bei einer Erkrankung Zeiten, in denen es einem plötzlich schlechter geht und man schnell einen Termin beim Arzt / Psychotherapeuten braucht.

PROG: Indikation = 3 | 5 | 6 | 7

Manchmal gibt es bei einer Erkrankung Zeiten, in denen es einem plötzlich schlechter geht und man schnell einen Termin beim Arzt braucht.

PROG: kein Filter

Denken Sie bitte für die folgenden Fragen an die letzten 3 Monate, d.h. [Monate] zurück.

515n (T0_n.a.) PROG: Indikation = 1 | 2 | 4

Gab es für Sie in den letzten 3 Monaten einen Zeitpunkt, an dem Sie aufgrund der Verschlechterung Ihrer seelischen Erkrankung schnell einen Behandlungstermin benötigten?

PROG: Indikation = 3 | 5 | 6 | 7

Gab es für Sie in den letzten 3 Monaten einen Zeitpunkt, an dem Sie aufgrund der Verschlechterung Ihrer Erkrankung des Nervensystems schnell einen Behandlungstermin benötigten?

❑… ja (1) ❑… nein (0)

515_1n (T0_n.a.) PROG: Frage 515 = 1 (ja), Indikation = 1 | 2 | 4

Wie lange mussten Sie auf diesen Behandlungstermin bei Ihrem Bezugsarzt / Bezugspsychotherapeuten warten?

PROG: Frage 515 = 1 (ja), Indikation = 3 | 5 | 6 | 7

Wie lange mussten Sie auf diesen Behandlungstermin bei Ihrem Bezugsarzt warten?

Bis zu einem Tag	2-3 Tage	4-7 Tage	7-14 Tage	länger als 14 Tage
1	2	3	4	5

515_3n PROG: Frage 515 = 1 (ja)

Haben Sie aufgrund von zu langen Wartezeiten auf einen Termin eine Notaufnahme aufgesucht?

**(T0_n.a.)**	❑… ja (1) ❑… nein (0)

6. **KG: Versorgungsqualität (indikationsspezifisch)**

Seitenüberschrift: Versorgungsqualität

Qualität / Zufriedenheit / Bezugsarzt
Quelle: eigen

PROG: Extra Seite mit folgendem Text, nicht fett und größere Schrift

PROG: Gruppe = 2 & Indikation = 1 | 2 | 4
Auf den folgenden Seiten möchten wir gern mehr über die Behandlung Ihrer seelischen Erkrankung erfahren.

Es geht darum, wie Sie Ihre Behandlung erleben und wie zufrieden Sie mit Ihrer Behandlung sind. Wir sind dabei an Ihrer persönlichen Einschätzung interessiert. Es gibt keine richtigen oder falschen Angaben.
Bitte denken Sie daran, falls Sie den Fragebogen stellvertretend für jemanden ausfüllen, alle Fragen aus Sicht der erkrankten Person zu beantworten.

PROG: Gruppe = 2 & Indikation = 3 | 5 | 6 | 7
Auf den folgenden Seiten möchten wir gern mehr über die Behandlung Ihrer Erkrankung des Nervensystems erfahren.

Es geht darum, wie Sie Ihre Behandlung erleben und wie zufrieden Sie mit Ihrer Behandlung sind. Wir sind dabei an Ihrer persönlichen Einschätzung interessiert. Es gibt keine richtigen oder falschen Angaben.
Bitte denken Sie daran, falls Sie den Fragebogen stellvertretend für jemanden ausfüllen, alle Fragen aus Sicht der erkrankten Person zu beantworten.

PROG: teilweise Filter nach Indikation und nach Gruppe

PROG: Gruppe = 2 & Indikation = 1 | 2 | 4, auf jeder Seite bis Frage 509_1 anzeigen

Die folgenden Fragen beziehen sich auf Ihre Behandlung der seelischen Erkrankung.

PROG: Gruppe = 2 & Indikation = 3 | 5 | 6 | 7, auf jeder Seite bis Frage 509_1 anzeigen

Die folgenden Fragen beziehen sich auf Ihre Behandlung der Erkrankung des Nervensystems.

PROG: alle

Im Folgenden ist mit „Arzt / (Psycho-)therapeut / Neurologe / Psychiater“ immer auch die weibliche Form „Ärztin / (Psycho-)therapeutin / Neurologin / Psychiaterin“ eingeschlossen.

500 PROG: Indikation = 1 | 2 | 4

Bei welchem Arzt / Psychotherapeuten waren Sie in den letzten 3 Monaten mit Ihrer seelischen Erkrankung vorwiegend, d.h. die meiste Zeit, in Behandlung?

PROG: Indikation = 3 | 5 | 6 | 7

Bei welchem Arzt / Psychotherapeuten waren Sie in den letzten 3 Monaten mit Ihrer Erkrankung des Nervensystems vorwiegend, d. h. die meiste Zeit, in Behandlung?

- ❑ Hausarzt (1)
- ❑ Psychiater (2)

❑ Neurologe oder Nervenarzt (3)

❑ Psychotherapeut (4)

❑ anderer Arzt / Therapeut (5)

nämlich: __________________

509 PROG: Indikation = 1 | 2 | 4

Nehmen Sie für Ihre Behandlung in regelmäßigen Abständen Termine bei diesem Arzt / Psychotherapeuten wahr?

PROG: Indikation = 3 | 5 | 6 | 7

Nehmen Sie für Ihre Behandlung in regelmäßigen Abständen Termine bei diesem Arzt wahr?

❑ ja (1) ❑ nein (0)

509_1 PROG: Frage 509 = 1 (ja)

Wie häufig nehmen Sie regelmäßige Behandlungstermine wahr?

jede Woche	alle 2 Wochen	jeden Monat	alle 2-3 Monate	alle 4-6 Monate	seltener
1	2	3	4	5	**6**

511 PROG: Indikation = 1 | 2 | 4

Haben Sie in den letzten 3 Monaten Ihrer Behandlung Ihren Arzt / Psychotherapeuten, zu dem Sie vorwiegend gehen, gewechselt?

PROG: Indikation = 3 | 5 | 6 | 7

Haben Sie in den letzten 3 Monaten Ihrer Behandlung Ihren Arzt, zu dem Sie vorwiegend gehen, gewechselt?

❑ ja (1) ❑ nein (0)

511_1 PROG: Frage 511 = 1 (ja)

nämlich _____________ Mal (numerisch, dreistellig, > 0)

PROG: Indikation = 1 | 2 | 4; auf jeder Seite bis Frage 505 anzeigen

Bitte beziehen Sie die folgenden Fragen auf diesen Arzt / Psychotherapeuten, bei dem Sie vorwiegend in Behandlung waren.

PROG: Indikation = 3 | 5 | 6 | 7; auf jeder Seite bis Frage 505 anzeigen

Bitte beziehen Sie die folgenden Fragen auf diesen Arzt, bei dem Sie vorwiegend in Behandlung waren.

PROG: kein Filter

Wie schätzen Sie die Qualität der Behandlung in den letzten 3 Monaten ein?

501 PROG: Indikation = 1 | 2 | 4

In meiner Behandlung nimmt sich mein Arzt / Psychotherapeut immer genug Zeit für mich.

PROG: Indikation = 3 | 5 | 6 | 7

In meiner Behandlung nimmt sich mein Arzt immer genug Zeit für mich.

stimmt überhaupt nicht	stimmt nicht	weder noch	stimmt	stimmt voll und ganz
1	2	3	4	5

502 PROG: kein Filter

In meiner Behandlung fühle ich mich sehr gut betreut.

stimmt überhaupt nicht	stimmt nicht	weder noch	stimmt	stimmt voll und ganz
1	2	3	4	5

503 **Meine Behandlung deckt meine Bedürfnisse voll und ganz ab.**

stimmt überhaupt nicht	stimmt nicht	weder noch	stimmt	stimmt voll und ganz
1	2	3	4	5

503_1 PROG: Indikation = 1 | 2 | 4

In meiner Behandlung hat mir mein Arzt / Psychotherapeut ausführlich erklärt, was ich bei Notfällen und Krisen, zum Beispiel wenn es mir plötzlich schlechter geht, tun soll.

PROG: Indikation = 3 | 5 | 6 | 7

In meiner Behandlung hat mir mein Arzt ausführlich erklärt, was ich bei Notfällen und Krisen, zum Beispiel wenn es mir plötzlich schlechter geht, tun soll.

stimmt überhaupt nicht	stimmt nicht	weder noch	stimmt	stimmt voll und ganz
1	2	3	4	5

503_2 PROG: Indikation = 1 | 2 | 4

In meiner Behandlung hat mich mein Arzt / Psychotherapeut ausführlich informiert, dass ich in Notfällen und Krisen schnell einen Termin bei ihm bekomme.

PROG: Indikation = 3 | 5 | 6 | 7

In meiner Behandlung hat mich mein Arzt ausführlich informiert, dass ich in Notfällen und Krisen schnell einen Termin bei ihm bekomme.

stimmt überhaupt nicht	stimmt nicht	weder noch	stimmt	stimmt voll und ganz
1	2	3	4	5

504 PROG: Indikation = 1 | 2 | 4

	**In meiner Behandlung erklärte mir mein Arzt / Psychotherapeut meinen Therapieverlauf einfach und verständlich.**<br>PROG: Indikation = 3 | 5 | 6 | 7<br>**In meiner Behandlung erklärte mir mein Arzt meinen Therapieverlauf einfach und verständlich.**

stimmt überhaupt nicht	stimmt nicht	weder noch	stimmt	stimmt voll und ganz
1	2	3	4	5

**510**	PROG: Indikation = 1 | 2 | 4<br>**In meiner Behandlung arbeiten verschiedene Ärzte / Psychotherapeuten gut zusammen und informieren sich gegenseitig.**<br>PROG: Indikation = 3 | 5 | 6 | 7<br>**In meiner Behandlung arbeiten verschiedene Ärzte gut zusammen und informieren sich gegenseitig.**

stimmt überhaupt nicht	stimmt nicht	weder noch	stimmt	stimmt voll und ganz
1	2	3	4	5

**510_1**	PROG: Indikation = 1 | 2 | 4<br>**In meiner Behandlung vermittelt mich mein Arzt / Psychotherapeut bei Bedarf an kompetente Stellen weiter (z. B. Physio- / Ergotherapie, anderer Facharzt).**<br>PROG: Indikation = 3 | 5 | 6 | 7<br>**In meiner Behandlung vermittelt mich mein Arzt bei Bedarf an kompetente Stellen weiter (z. B. Physio- / Ergotherapie, anderer Facharzt).**

stimmt überhaupt nicht	stimmt nicht	weder noch	stimmt	stimmt voll und ganz
1	2	3	4	5

**505**	PROG: Indikation = 1 | 2 | 4<br>**In meiner Behandlung empfahl mir mein Arzt / Psychotherapeut sinnvolle therapeutische Angebote.**<br>PROG: Indikation = 3 | 5 | 6 | 7<br>**In meiner Behandlung empfahl mir mein Arzt sinnvolle therapeutische Angebote.**

stimmt überhaupt nicht	stimmt nicht	weder noch	stimmt	stimmt voll und ganz
1	2	3	4	5

Gruppenangebote

**506**	PROG: Indikation = 1 | 2 | 4

Wurden Ihnen in Ihrer Behandlung von Ihrem Arzt / Psychotherapeuten Gruppenangebote, die nicht in einem Krankenhaus stattfanden, zur Unterstützung der Therapie empfohlen?

PROG: Indikation = 3 | 5 | 6 | 7

Wurden Ihnen in Ihrer Behandlung von Ihrem Arzt Gruppenangebote, die nicht in einem Krankenhaus stattfanden, zur Unterstützung der Therapie empfohlen?

❑ ja (1) ❑ nein (0)

Wie wurden Sie über das Gruppenangebot informiert?

506_01 PROG: Frage 506 = 1 (ja) & Indikation = 1 | 2 | 4

Mein Arzt / Psychotherapeut beschrieb mir ausführlich den Inhalt und Ablauf des Gruppenangebots.

PROG: Frage 506 = 1 (ja) & Indikation = 3 | 5 | 6 | 7

Mein Arzt beschrieb mir ausführlich den Inhalt und Ablauf des Gruppenangebots.

stimmt überhaupt nicht	stimmt nicht	weder noch	stimmt	stimmt voll und ganz
1	2	3	4	5

506_02 PROG: Frage 506 = 1 (ja) & Indikation = 1 | 2 | 4

Mein Arzt / Psychotherapeut erläuterte mir genau, warum das Gruppenangebot für mich hilfreich ist.

PROG: Frage 506 = 1 (ja) & Indikation = 3 | 5 | 6 | 7

Mein Arzt erläuterte mir genau, warum das Gruppenangebot für mich hilfreich ist.

stimmt überhaupt nicht	stimmt nicht	weder noch	stimmt	stimmt voll und ganz
1	2	3	4	5

506_03 PROG: Frage 506 = 1 (ja) & Indikation = 1 | 2 | 4

Mein Arzt / Psychotherapeut bzw. das Praxisteam unterstützte mich sehr dabei, das Gruppenangebot wahrzunehmen.

PROG: Frage 506 = 1 (ja) & Indikation = 3 | 5 | 6 | 7

Mein Arzt bzw. das Praxisteam unterstützte mich sehr dabei, das Gruppenangebot wahrzunehmen

stimmt überhaupt nicht	stimmt nicht	weder noch	stimmt	stimmt voll und ganz
1	2	3	4	5

PROG: Frage 506 = 1 (ja)

Welche Gruppenangebote wurden Ihnen empfohlen und haben Sie diese wahrgenommen?

Gruppenangebot	empfohlen	wahrgenommen

506_11<br>506_12	Gruppenangebot für Betroffene	ja (1) / nein (0)	ja (1) / nein (0)
506_21<br>506_22	Gruppenangebot für Angehörige	ja (1) / nein (0)	ja (1) / nein (0)

506_5 PROG: Frage 506 = 1 (ja) & mind. einmal (Frage 506_x1 = 1 (ja) & Frage 506_x2 = 0 (nein))

506_6 **Sie haben angegeben, dass Sie mindestens an einem Gruppenangebot, das Ihnen empfohlen wurde, nicht teilgenommen haben.**

506_7

506_71 **Welche Gründe hatten Sie dafür?**

506_8 PROG: Mehrfachantwort möglich

506_8o

- ❑ Ich habe kein Interesse daran. (1 genannt; 0 nicht genannt)
- ❑ Der Aufwand ist mir zu groß. (1 genannt; 0 nicht genannt)
- ❑ Der Weg ist mir zu weit. (1 genannt; 0 nicht genannt)
- ❑ Ich glaube nicht, dass mir das Angebot hilft. (1 genannt; 0 nicht genannt)
- ❑ Ich möchte meine Probleme nicht vor anderen Patienten besprechen. (1 genannt; 0 nicht genannt)
- ❑ anderer Grund (1 genannt; 0 nicht genannt)

PROG: Frage 506_8 = 1 (anderer Grund)

Und zwar: _____________

PROG: Frage 506 = 1 (ja) & mind. einmal (Frage 506_x2 = 1 (ja))

Sie haben an mindestens einem Gruppenangebot teilgenommen. Bitte geben Sie an, inwiefern Sie den folgenden Aussagen zustimmen.

506_9 PROG: Frage 506 = 1 (ja) & mind. einmal (Frage 506_x2 = 1 (ja))

Das Gruppenangebot hat mir sehr geholfen, im Alltag mit meiner Erkrankung zurecht zu kommen.

stimmt überhaupt nicht	stimmt nicht	weder noch	stimmt	stimmt voll und ganz
1	2	3	4	5

506_010 PROG: Frage 506 = 1 (ja) & mind. einmal (Frage 506_x2 = 1 (ja))

Durch das Gruppenangebot belastet mich meine Erkrankung deutlich weniger.

stimmt überhaupt nicht	stimmt nicht	weder noch	stimmt	stimmt voll und ganz
1	2	3	4	5

506_011 PROG: Frage 506 = 1 (ja) & mind. einmal (506_x2 = 1 (ja))

Durch das Gruppenangebot verstehe ich meine Erkrankung besser.

stimmt überhaupt nicht	stimmt nicht	weder noch	stimmt	stimmt voll und ganz
1	2	3	4	5

506_012 PROG: Frage 506 = 1 (ja) & mind. einmal (Frage 506_x2 = 1 (ja))

Der Austausch mit anderen Betroffenen beim Gruppenangebot hat mir sehr geholfen.

stimmt überhaupt nicht	stimmt nicht	weder noch	stimmt	stimmt voll und ganz
1	2	3	4	5

Onlineangebote

507 PROG: kein Filter

Wurde Ihnen in ihrer Behandlung Online-Selbsthilfe zur Unterstützung der Therapie empfohlen?

❑ ja (1) ❑ nein (0)

Wie wurden Sie über die Online-Selbsthilfe informiert?

507_01 PROG: Frage 507 = 1 (ja) & Indikation = 1 | 2 | 4

Mein Arzt / Psychotherapeut beschrieb mir ausführlich den Inhalt und Ablauf der Online-Selbsthilfe.

PROG: Frage 507 = 1 (ja) & Indikation = 3 | 5 | 6 | 7

Mein Arzt beschrieb mir ausführlich den Inhalt und Ablauf der Online-Selbsthilfe.

stimmt überhaupt nicht	stimmt nicht	weder noch	stimmt	stimmt voll und ganz
1	2	3	4	5

507_02 PROG: Frage 507 = 1 (ja) & Indikation = 1 | 2 | 4

Mein Arzt / Psychotherapeut erläuterte mir genau, warum die Online-Selbsthilfe für mich hilfreich ist.

PROG: Frage 507 = 1 (ja) & Indikation = 3 | 5 | 6 | 7

Mein Arzt erläuterte mir genau, warum die Online-Selbsthilfe für mich hilfreich ist.

stimmt überhaupt nicht	stimmt nicht	weder noch	stimmt	stimmt voll und ganz
1	2	3	4	5

507_1 PROG: Frage 507 = 1 (ja)

Haben Sie dieses Angebot der Online-Selbsthilfe wahrgenommen?

❑ ja (1) ❑ nein (0)

507_5 PROG: Frage 507 = 1 (ja) & Frage 507_1 = 0 (nein)

507_6 **Sie haben angegeben, dass Sie an einer Online-Selbsthilfe, die Ihnen empfohlen wurde, nicht teilgenommen haben.**

507_7 **Welche Gründe haben Sie dafür?**

507_8 PROG: Mehrfachantwort möglich

507_8o

- ❑ Ich habe kein Interesse daran. (1 genannt; 0 nicht genannt)
- ❑ Der Aufwand ist mir zu groß. (1 genannt; 0 nicht genannt)
- ❑ Ich glaube nicht, dass mir die Online-Selbsthilfe hilft. (1 genannt; 0 nicht genannt)
- ❑ anderer Grund (1 genannt; 0 nicht genannt)

PROG: Frage 507_8 = 1 (anderer Grund)

Und zwar: _____________

PROG: Frage 507 = 1 (ja) & Frage 507_1 = 1 (ja)

Sie haben bereits an einer Online-Selbsthilfe teilgenommen. Bitte geben Sie an, inwiefern Sie den folgenden Aussagen zustimmen.

507_9 PROG: Frage 507 = 1 (ja) & Frage 507_1 = 0 (nein)

Die Online-Selbsthilfe hat mir sehr geholfen, im Alltag mit meiner Erkrankung zurecht zu kommen.

stimmt überhaupt nicht	stimmt nicht	weder noch	stimmt	stimmt voll und ganz
1	2	3	4	5

507_010 PROG: Frage 507 = 1 (ja) & Frage 507_1 = 0 (nein)

Durch die Online-Selbsthilfe belastet mich meine Erkrankung deutlich weniger.

stimmt überhaupt nicht	stimmt nicht	weder noch	stimmt	stimmt voll und ganz
1	2	3	4	5

507_011 PROG: Frage 507 = 1 (ja) & Frage 507_1 = 0 (nein)

Durch die Online-Selbsthilfe verstehe ich meine Erkrankung besser.

stimmt überhaupt nicht	stimmt nicht	weder noch	stimmt	stimmt voll und ganz
1	2	3	4	5

Schübe
Quelle: eigen
PROG: Indikation =5

516 PROG: Indikation = 5

Haben Sie in den letzten drei Monaten, d.h. [Monate] einen Schub erlebt?

❑ ja (1) ❑ nein (0)

516_1 PROG: Indikation = 5 & Frage 516 = 1 (Schub erlebt)

Haben Sie in dieser Zeit eine Schubtherapie gemacht?

❑ ja, ambulant bei meinem behandelnden Arzt (1)

❑ ja, in einem Krankenhaus (2)

❑ nein (0)

Entlassungsmanagement
Quelle: eigen

512 PROG: Indikation = 1 | 2 | 4

Wie viele Nächte waren Sie in den vergangenen 3 Monaten, d.h. [Monate] aufgrund Ihrer seelischen Erkrankung zur stationären Behandlung in einem Krankenhaus?

PROG: Indikation = 3 | 5 | 6 | 7

Wie viele Nächte waren Sie in den vergangenen 3 Monaten, d.h. [Monate] aufgrund Ihrer Erkrankung des Nervensystems zur stationären Behandlung in einem Krankenhaus?

____ Nächte (numerisch, 3-stellig, max: 120)

512_1 PROG: Frage 512 > 0 & Indikation = alle

Versuchen Sie sich an die letzte Entlassung aus dem Krankenhaus zu erinnern.

PROG: Indikation = 1 | 2 | 4

Wie viel Zeit ist zwischen Ihrer Entlassung und Ihrem ersten Termin bei Ihrem niedergelassenen Arzt / Psychotherapeuten verstrichen?

PROG: Indikation = 3 | 5 | 6 | 7

Wie viel Zeit ist zwischen Ihrer Entlassung und Ihrem ersten Termin bei Ihrem niedergelassenen Arzt verstrichen?

bis zu 7 Tage	1 bis 2 Wochen	3 bis 4 Wochen	1 bis 3 Monate	länger als 3 Monate
1	2	3	4	5

Behandlungsabbrüche

Quelle: eigen

PROG: teilweise nach Indikation gefiltert

PROG: kein Filter

Manchmal hat man das Gefühl, dass eine Behandlung nicht so gut hilft oder einem beispielsweise aufgrund von Nebenwirkungen nicht guttut. Manche Patienten brechen deshalb eine Therapie gegen den

ärztlichen Rat ab. Zum Beispiel gehen Sie zu vorgesehenen Folgeterminen nicht mehr hin oder nehmen die Medikamente nicht mehr wie vereinbart.

513 PROG: Indikation = 1 | 2 | 4

Haben Sie in den letzten 3 Monaten die Behandlung Ihrer seelischen Erkrankung gegen ärztlichen Rat abgebrochen?

PROG: Indikation = 3 | 5 | 6 | 7

Haben Sie in den letzten 3 Monaten die Behandlung Ihrer Erkrankung des Nervensystems gegen ärztlichen Rat abgebrochen?

❑... ja (1) ❑... nein (0)

Fragen zu Krisensituationen
Quelle: eigen

PROG: teilweise nach Indikation gefiltert

PROG: Indikation = 1 | 2 | 4

Manchmal gibt es bei einer Erkrankung Zeiten, in denen es einem plötzlich schlechter geht und man schnell einen Termin beim Arzt / Psychotherapeuten braucht.

PROG: Indikation = 3 | 5 | 6 | 7

Manchmal gibt es bei einer Erkrankung Zeiten, in denen es einem plötzlich schlechter geht und man schnell einen Termin beim Arzt braucht.

PROG: kein Filter

Denken Sie bitte für die folgenden Fragen an die letzten 3 Monate, d.h. [Monate] zurück.

515 PROG: Indikation = 1 | 2 | 4

Gab es für Sie in den letzten 3 Monaten einen Zeitpunkt, an dem Sie aufgrund der Verschlechterung Ihrer seelischen Erkrankung schnell einen Behandlungstermin benötigten?

PROG: Indikation = 3 | 5 | 6 | 7

Gab es für Sie in den letzten 3 Monaten einen Zeitpunkt, an dem Sie aufgrund der Verschlechterung Ihrer Erkrankung des Nervensystems schnell einen Behandlungstermin benötigten?

❑... ja (1) ❑... nein (0)

515_1 PROG: Frage 515 = 1 (ja), Indikation = 1 | 2 | 4

Wie lange mussten Sie auf diesen Behandlungstermin bei Ihrem niedergelassenen Arzt / Psychotherapeuten warten?

PROG: Frage 515 = 1 (ja), Indikation = 3 | 5 | 6 | 7

Wie lange mussten Sie auf diesen Behandlungstermin bei Ihrem niedergelassenen Arzt warten?

Bis zu einem Tag	2-3 Tage	4-7 Tage	7-14 Tage	länger als 14 Tage
1	2	3	4	5

515_3 PROG: Frage 515 = 1 (ja)

Haben Sie aufgrund von zu langen Wartezeiten auf einen Termin eine Notaufnahme aufgesucht?

❑… ja (1) ❑… nein (0)

7. Abschluss

710 PROG: kein Filter

Bitte geben Sie zum Abschluss noch an, wie Sie den Fragebogen ausgefüllt haben.

❑ vorwiegend alleine (1)

❑ zusammen mit einer anderen Person (2)

❑ vorwiegend eine andere Person (3)

711o **Haben Sie noch weitere Anregungen für uns?**

VIELEN DANK FÜR IHRE TEILNAHME!

Die Befragung zu Ihrer Versorgung ist hiermit vollständig beendet. Wir danken Ihnen noch einmal herzlich für Ihre Unterstützung!
