# Supplementary material for: A Stepped Health Services Intervention to Improve Care for Mental and Neurological Diseases: Protocol for a Prospective Cohort Trial
Source: JMIR Res Protoc. 2023 Jan 17;12:e37569. doi: 10.2196/37569 (PMC9890347; doi:10.2196/37569)
Supplement: Multimedia Appendix 2 [file resprot_v12i1e37569_app2.pdf]

Fragebogen zur Evaluation des Projekts „Verbesserte Versorgung psychischer und neurologischer Erkrankungen“ (NPPV)

Erhebungswelle 2019

Im Auftrag der Kassenärztlichen Vereinigung Nordrhein

Kassenärztliche Vereinigung Nordrhein

ZENTRALINSTITUT FÜR DIE KASSENÄRZTLICHE VERSORGUNG IN DEUTSCHLAND

Hinweise:

Da der Fragebogen mit moderner Scanner-Technik ausgewertet wird, bitten wir Sie,

- einen schwarzen oder blauen Kugelschreiber zu verwenden,
- Ihre Angaben gut lesbar und Markierungen innerhalb der Kästchen zu machen,
- keine Anmerkungen außerhalb von Erfassungsfeldern in den Fragebogen zu schreiben,
- eventuelle Korrekturen wie folgt vorzunehmen:
  - falsch gesetzte Kreuze in Kästchen komplett schwärzen,
  - falsch eingetragene Werte komplett durchstreichen, den korrigierten Wert neben das durchgestrichene Feld neu eintragen und eindeutig zuordnen.

Aus Gründen der besseren Lesbarkeit wird auf eine neutrale Sprachform zurückgegriffen. Sämtliche Personenbezeichnungen (z. B. Therapeut, Arzt, Patient, Kollege) gelten gleichermaßen für alle Geschlechter.

Copyright © Zentralinstitut für die kassenärztliche Versorgung

TEIL A

Angaben zum Versorgungsprojekt NPPV

Das Versorgungsprojekt NPPV wurde mit dem Ziel geschaffen, die ambulante Versorgung von Menschen mit neurologischen und psychischen Erkrankungen zu verbessern. Anhand des vorliegenden Fragebogens sollen erarbeitete Strukturen und Prozesse bewertet werden. Mit Ihrer Teilnahme an der Befragung tragen Sie dazu bei, das Versorgungsprojekt NPPV auch in Zukunft weiterentwickeln zu können.

Copyright © Zentralinstitut für die kassenärztliche Versorgung

Angaben zum Status Ihrer NPPV-Teilnahme

A1 Seit wann nehmen Sie am NPPV-Projekt teil? M M / J J J J

A2 Ungefähr wie viele Ihrer Patienten sind derzeit in das NPPV-Projekt eingeschrieben? ca. ☐☐☐

A3 Im Vergleich zu einem Patienten, der nicht im NPPV-Projekt eingeschrieben ist, fallen die Kontaktzahlen eines **NPPV-Patienten** schätzungsweise wie folgt aus:

☐ ca. ___ Kontakte **weniger** pro Quartal	☐ ca. **gleich** viele Kontakte pro Quartal	☐ ca. ___ Kontakte **mehr** pro Quartal

Strukturen und Prozesse: Allgemein

A4.1 Wenn Sie an das Projekt NPPV denken, wie schätzen Sie …

	sehr gut	gut	befriedigend	ausreichend	mangelhaft
den Austausch/die Zusammenarbeit zwischen allen Fachgruppen innerhalb des NPPV Projekts ein?	☐	☐	☐	☐	☐
den fallbezogenen Austausch zwischen Bezugsarzt/-therapeut und anderen an der Versorgung beteiligten Behandlern ein?	☐	☐	☐	☐	☐
die Einbindung therapeutischer Angebote, wie bspw. Gruppen- und Online-Selbsthilfeangebote (Novego), ein?	☐	☐	☐	☐	☐
die Unterstützung der Netzwerke durch Koordinationsstellen oder IT-Strukturen hinsichtlich der Verkürzung von Wartezeiten ein?	☐	☐	☐	☐	☐
die Unterstützung der Netzwerke durch Koordinationsstellen oder IT-Strukturen hinsichtlich der Priorisierung von Behandlungsfällen ein?	☐	☐	☐	☐	☐

Strukturen und Prozesse: IT-Systeme

A4.2 Wenn Sie an das Projekt NPPV denken, wie bewerten Sie …

	sehr gut	gut	befriedigend	ausreichend	mangelhaft
die Vertragssoftware IVPnet im Allgemeinen?	☐	☐	☐	☐	☐
die Vertragssoftware IVPnet im Hinblick auf die Verwaltung von Patientendaten?	☐	☐	☐	☐	☐
die Vertragssoftware IVPnet im Hinblick auf eine bessere Umsetzung und Verwaltung von Behandlungspfaden?	☐	☐	☐	☐	☐
die Vertragssoftware IVPnet im Hinblick auf den Informationsaustausch zwischen allen an der Versorgung Beteiligten?	☐	☐	☐	☐	☐
die Sicherheit der Vertragssoftware IVPnet (u. A. Einhaltung des Datenschutzes)?	☐	☐	☐	☐	☐

Copyright © Zentralinstitut für die kassenärztliche Versorgung

Ergebniseinschätzung: Arbeitsbelastung

A5.1 Durch die Implementierung des Projekts NPPV hat sich…

	stark verringert	verringert	nicht verändert	erhöht	stark erhöht
die allgemeine Arbeitsbelastung	☐	☐	☐	☐	☐
die aufgewendete durchschnittliche Anzahl der Wochenstunden für die Patientenversorgung	☐	☐	☐	☐	☐
die Arbeitsbelastung durch Dokumentationen	☐	☐	☐	☐	☐
die Zufriedenheit des Praxispersonals	☐	☐	☐	☐	☐

Ergebniseinschätzung: Versorgungsqualität

A5.2 Durch die Implementierung des Projekts NPPV hat sich …

	stark verringert	verringert	nicht verändert	erhöht	stark erhöht
die Zahl der Therapieabbrüche durch den Patienten	☐	☐	☐	☐	☐
die Zahl der nötigen Krankenhauseinweisungen	☐	☐	☐	☐	☐
die Produktivität in der Praxis	☐	☐	☐	☐	☐
der Verweis an weiterführende therapeutische Angebote	☐	☐	☐	☐	☐
die Ausrichtung auf präventive Maßnahmen	☐	☐	☐	☐	☐
	**stark verbessert**	**verbessert**	**nicht verändert**	**vermindert**	**stark vermindert**
die Qualität der Patientenversorgung	☐	☐	☐	☐	☐
die Beziehung/Bindung zu den von Ihnen betreuten Patienten im Allgemeinen	☐	☐	☐	☐	☐
die Möglichkeit, zielgenaue und flexible Therapie-optionen bereitzustellen	☐	☐	☐	☐	☐
der allgemeine Therapieerfolg bei Patienten	☐	☐	☐	☐	☐
die Möglichkeit, Krisen und Krankheitsschübe der Patienten schneller und besser zu behandeln (intensivierte ambulante Komplexbehandlung)	☐	☐	☐	☐	☐
die Zusammenarbeit mit den Arbeitsstätten der Patienten	☐	☐	☐	☐	☐
die Zusammenarbeit mit ambulanten Spezialkliniken	☐	☐	☐	☐	☐
die fachärztliche/therapeutische Versorgungsintensität	☐	☐	☐	☐	☐
die (über)regionale Vernetzung	☐	☐	☐	☐	☐
der Überblick der Patienten über ihren eigenen Gesundheitszustand	☐	☐	☐	☐	☐

Copyright © Zentralinstitut für die kassenärztliche Versorgung

Persönliche Bewertung

A6.1 Wie wichtig sind Ihnen folgende Ziele bei der Teilnahme am Projekt NPPV?

	sehr	ziemlich	wenig	gar nicht
Steigerung der Versorgungsqualität spezifischer Patientengruppen	☐	☐	☐	☐
Verringerung von Therapieabbrüchen	☐	☐	☐	☐
Effizientere Steuerung der Patientenversorgung in der Praxis	☐	☐	☐	☐
Steigerung des Praxisumsatzes	☐	☐	☐	☐
Steigerung der Patientenbindung und -zufriedenheit	☐	☐	☐	☐
Steigerung der eigenen Arbeitszufriedenheit	☐	☐	☐	☐
Möglichkeit ein innovatives Konzept mitzugestalten	☐	☐	☐	☐
Berufsgruppenübergreifende Vernetzung	☐	☐	☐	☐
Erweiterung der therapeutischen Versorgung durch Gruppen- und Online-Selbsthilfeangebote (Novego)	☐	☐	☐	☐

Sonstiges (bitte benennen):

A6.2 Wie zufrieden sind Sie mit …

	sehr zufrieden	zufrieden	teils, teils oder neutral	unzufrieden	sehr unzufrieden
Ihrer momentanen beruflichen Situation?	☐	☐	☐	☐	☐
dem Projekt NPPV im Allgemeinen?	☐	☐	☐	☐	☐
der Vergütung des erhöhten Versorgungsaufwands innerhalb des Projekts NPPV?	☐	☐	☐	☐	☐
dem Erreichen der Ziele, die Sie mit der Teilnahme beim Projekt NPPV verfolgen?	☐	☐	☐	☐	☐
den Schulungen für das Projekt NPPV?	☐	☐	☐	☐	☐
den Netzwerktreffen für das Projekt NPPV?	☐	☐	☐	☐	☐
den Qualitätszirkeln für das Projekt NPPV?	☐	☐	☐	☐	☐
der Unterstützung der Koordinationsstellen hinsichtlich der Umsetzung der allgemeinen Strukturen und Prozesse des Projekts NPPV?	☐	☐	☐	☐	☐

Sonstiges (bitte benennen):

Copyright © Zentralinstitut für die kassenärztliche Versorgung

A6.3 Wie zutreffend sind diese Aussagen aus Ihrer Sicht?

	sehr	ziemlich	wenig	gar nicht
Ich werde das Projekt auch in Zukunft weiter unterstützen	☐	☐	☐	☐
Das Projekt ist für die Erkrankten angemessen	☐	☐	☐	☐
Es fällt mir leicht, leitliniengerechte Behandlungspfade/ definierte Prozesse des Projekts einzuhalten	☐	☐	☐	☐
Durch das Projekt habe ich angefangen, therapeutische Gruppenangebote für Patienten anzubieten	☐	☐	☐	☐
Die meisten Patienten profitieren von dem Projekt	☐	☐	☐	☐
Das Projekt trägt zur Entlastung in der Patientenversorgung bei	☐	☐	☐	☐
Meine Fachkompetenzen sind ausreichend, um das Projekt leitliniengerecht auszuführen	☐	☐	☐	☐
Ich erhalte alle notwendigen Informationen, die für meine Arbeit innerhalb des Projekts essentiell sind	☐	☐	☐	☐
Die Patienten erhalten durch das Projekt mehr Werkzeuge für eine erfolgreiche Therapie	☐	☐	☐	☐
Die Öffentlichkeitsarbeit für das Projekt ist ausreichend	☐	☐	☐	☐
Ich würde das Projekt Kollegen weiterempfehlen	☐	☐	☐	☐
**… bzw. wie zutreffend bewerten Sie diese Gründe für den Austritt der Patienten aus dem NPPV-Projekt?**				
Untauglichkeit der Patienten wegen kognitiver Einschränkungen, Demenz o. Ä.	☐	☐	☐	☐
Patienten erkennen die Relevanz der Therapieempfehlung nicht	☐	☐	☐	☐
Angst der Patienten sich selbst/sich der Familie/dem sozialen Umfeld zu öffnen	☐	☐	☐	☐
Mangelnde Unterstützung der Familie/des sozialen Umfeldes/ der Arbeitsstätte der Patienten	☐	☐	☐	☐
Schwierigkeiten der Patienten, ihre Situation zu akzeptieren	☐	☐	☐	☐
Ungenügender Zugang zum Versorgernetz oder anderen Therapieeinrichtungen	☐	☐	☐	☐
Mangelndes Wissen über das Projekt hinsichtlich seiner Ziele, Strukturen und Prozesse	☐	☐	☐	☐
Überforderung der Patienten durch Evaluationen	☐	☐	☐	☐
Sonstiges (bitte benennen):				

A6.4 Was sollte aus Ihrer Sicht in Zukunft am Projekt NPPV verbessert werden?

Copyright © Zentralinstitut für die kassenärztliche Versorgung

TEIL B

Angaben zur Person

Zum Abschluss bitten wir Sie um Angaben zu Ihrer Person.
Alle Daten werden anonym erfasst und nur für wissenschaftliche Forschungszwecke ausgewertet.

Copyright © Zentralinstitut für die kassenärztliche Versorgung

Angaben zum Versorger und der Versorgungstätigkeit

B1 **In welchem Jahr sind Sie geboren?** J J J J

B2 **Seit wie vielen Jahren sind Sie als niedergelassener Arzt/Psychotherapeut tätig?** ☐☐ Jahre

B3 **Welches Geschlecht haben Sie?** weiblich ☐ männlich ☐

B4 **Welche waren, bezogen auf den Umsatz, Ihre wichtigsten Zulassungsfachgebiete am Stichtag 31.12.2018?**
Die Kodierung der Zulassungsfachgebiete finden Sie auf Seite 11.

1. Zulassungsfachgebiet	2. Zulassungsfachgebiet	3. Zulassungsfachgebiet
☐☐☐	☐☐☐	☐☐☐

B5 **Welche Facharztbezeichnung gemäß Weiterbildungsordnung hatten Sie am Stichtag 31.12.2018?**
Die Kodierung der Facharztbezeichnung finden Sie auf Seite 11.

1. Facharztbezeichnung	2. Facharztbezeichnung	3. Facharztbezeichnung
☐☐☐	☐☐☐	☐☐☐

B6 **Wie viele ärztliche bzw. psychotherapeutisch tätige Vollzeit-, und Teilzeitbeschäftigte haben am Stichtag 31.12.2018 in Ihrer Praxis gearbeitet - Sie selbst mit eingerechnet?** Bitte machen Sie die Angabe in Stellen.

Vollzeit	☐☐
Teilzeit	☐☐

B7 **Welche Beschäftigungsform traf auf Sie am Stichtag 31.12.2018 zu?**

selbstständig	☐
angestellt	☐

B8 **Wie viele Patienten wurden im 4. Quartal 2018 in Ihrer Praxis versorgt?** ☐☐☐☐

B9 **Wie viele Wochenstunden haben Sie im Jahr 2018 durchschnittlich für die Patientenversorgung aufgewendet und wie verteilen sich diese auf ärztliche/psychotherapeutische Tätigkeiten?**

**Wochenstunden für Patientenversorgung**		**auf ärztliche/psychotherapeutische Tätigkeiten verteilt, entfallen von den Wochenstunden ...**				
☐☐	=	☐☐ Std./Wo.	+	☐☐ Std./Wo.	+	☐☐ Std./Wo.
**ohne Fortbildungen, ohne Praxismanagement**		**mit Patienten** (Beratung, Untersuchung und Behandlung)		**ohne Patienten** (z. B. Dokumentationen und Gutachten)		**Notfalleinsätze** (ohne Zeiten der reinen Bereitschaft)

B10 **Arbeiten Sie derzeit in einem Arztnetz bzw. sind Sie als Psychotherapeut in einem Arztnetz eingebunden?** ja ☐ nein ☐

Copyright © Zentralinstitut für die kassenärztliche Versorgung

**B11**	**Welche Organisationsform traf auf Ihre Praxis am Stichtag 31.12.2018 zu?**
Einzelpraxis	☐
Örtliche Berufsausübungsgemeinschaft	☐
Überörtliche Berufsausübungsgemeinschaft	☐
Medizinisches Versorgungszentrum	☐
Andere (bitte benennen):	

B12 An welchen besonderen Versorgungsformen nimmt bzw. nahm Ihre Praxis in der Vergangenheit teil?

	ja	nein
Strukturierte Behandlungsprogramme (DMP) gemäß § 137f SGB V	☐	☐
Integrierte Versorgung gemäß § 140a – d SGB V *	☐	☐
Besondere Versorgung gemäß § 140a SGB V **	☐	☐
Besondere ambulante ärztliche Versorgung gemäß § 73c SGB V	☐	☐
Ambulante spezialfachärztliche Versorgung (ASV) gemäß § 116b SGB V	☐	☐

*Verträge, die vor Juli 2015 abgeschlossen wurden. **Verträge, die auf Basis der neuen Fassung des § 140a SGB V abgeschlossen wurden.

B13 Unabhängig des NPPV Projekts - Wie zufrieden sind Sie innerhalb der Regelversorgung mit ...

	sehr zufrieden	zufrieden	teils, teils oder neutral	unzufrieden	sehr unzufrieden
Ihren momentanen Arbeitsbedingungen?	☐	☐	☐	☐	☐
Ihrer Arbeit im Allgemeinen?	☐	☐	☐	☐	☐
der Zeit, die Ihnen für die Behandlung Ihrer Patienten zur Verfügung steht?	☐	☐	☐	☐	☐
der erfahrenen Anerkennung, die Sie für Ihre Leistungen und Mühen erhalten?	☐	☐	☐	☐	☐
Ihrem monatlichen Einkommen aus Ihrer Arbeit?	☐	☐	☐	☐	☐

B14 Was möchten Sie uns mitteilen? Hier finden Sie Platz für Anmerkungen und sonstige Kommentare.

Copyright © Zentralinstitut für die kassenärztliche Versorgung

KODIERUNG

von Zulassungsfachgebiet und Facharztbezeichnung

Allgemeine Chirurgie ... A01
Allgemeinmedizin ... A02
Anästhesiologie ... A03
Anästhesiologie und Intensivtherapie ... A04
Anatomie ... A05
Angiologie ... A06
Arbeitshygiene ... A07
Arbeitsmedizin ... A08
Arzt ... A09
Arzt für Kinder- und Jugendmedizin ... A10
Audiologie ... A11
Augenheilkunde ... A12

Blutspende- und Transfusionsmedizin ... B01

Chirurgie ... C01

Diabetologie ... D01
Diagnostische Radiologie ... D02

Echokardiologie herznaher Gefäße ... E01
Endokrinologie ... E02
Endokrinologie und Diabetologie ... E03
Experimentelle und diagnostische Mikrobiologie ... E04

Fachbiologie der Medizin ... F01
Fachwissenschaft Chemie und Labordiagnostik ... F02
Fachwissenschaft Genetik ... F03
Fachwissenschaft Immunologie ... F04
Fachwissenschaft Zytologie/Histologie ... F05
Forensische Psychiatrie ... F06
Frauenheilkunde und Geburtshilfe ... F07

Gastroenterologie ... G01
Gefäßchirurgie ... G02
Geriatrie ... G03
Gynäkologische Endokrinologie und Reproduktionsmedizin ... G04
Gynäkologische Onkologie ... G05

Hals-Nasen-Ohrenheilkunde ... H01
Hämatologie ... H02
Hämatologie und internistische Onkologie ... H03
Haut- und Geschlechtskrankheiten ... H04
Herz- und Gefäßchirurgie ... H05
Herzchirurgie ... H06
Humangenetik ... H07
Hygiene ... H08
Hygiene und Umweltmedizin ... H09

Immunologie ... I01
Infektiologie ... I02
Infektions- und Tropenmedizin ... I03
Innere Medizin ... I04
Innere Medizin: Angiologie ... I05
Innere Medizin: Endokrinologie und Diabetologie ... I06
Innere Medizin: Gastroenterologie ... I07
Innere Medizin: Hämatologie und Onkologie ... I08
Innere Medizin: Kardiologie ... I09
Innere Medizin: Nephrologie ... I10
Innere Medizin: Pneumologie ... I11
Innere Medizin: Rheumatologie ... I12
Innere und Allgemeinmedizin (Hausarzt) ... I13

Kardiologie ... K01
Kardiologie und Angiologie ... K02
Kieferchirurgie ... K03
Kinder- und Jugendlichen-Psychotherapeut ... K04
Kinder- und Jugendpsychiatrie ... K05
Kinder- und Jugendpsychiatrie und -psychotherapie ... K06
Kinderchirurgie ... K07
Kinderdiabetologie ... K08
Kinderendokrinologie und -diabetologie ... K09
Kindergastroenterologie ... K10
Kinderhämatologie ... K11
Kinderhämatologie und -onkologie ... K12
Kinderheilkunde ... K13
Kinderheilkunde und Jugendmedizin ... K14
Kinderkardiologie ... K15
Kinder-Lungen- und Bronchialheilkunde ... K16
Kindernephrologie ... K17
Kinderneuropsychiatrie ... K18
Kinderpneumologie ... K19
Kinderradiologie ... K20
Kinderrheumatologie ... K21
Kinder- und Jugendarzt ... K22
Kinder- und Jugendmedizin ... K23
Klinische Pharmakologie ... K24

Laboratoriumsmedizin ... L01
Lungen- und Bronchialheilkunde ... L02
Lungenarzt ... L03

Magenarzt ... M01
Mikrobiologie ... M02
Mikrobiologie und Infektionsepidemiologie ... M03
Mikrobiologie, Virologie und Infektionsepidemiologie ... M04
Mund-Kiefer-Gesichtschirurgie ... M05

Neonatologie ... N01
Nephrologie ... N02
Nervenheilkunde ... N03
Neurochirurgie ... N04
Neurologie ... N05
Neurologie und Psychiatrie ... N06
Neurologie, Psychiatrie und Psychotherapie ... N07
Neuropädiatrie ... N08
Neuropathologie ... N09
Neuroradiologie ... N10
Nuklearmedizin ... N11

Orthopädie ... O01
Orthopädie und Unfallchirurgie ... O02

Pathologie ... P01
Pathologische Anatomie ... P02
Pharmakologie und Toxikologie ... P03
Phoniatrie ... P04
Phoniatrie und Pädaudiologie ... P05
Physikalische und Rehabilitative Medizin ... P06
Physiologie ... P07
Physiotherapie ... P08
Plastische Chirurgie ... P09
Plastische und Ästhetische Chirurgie ... P10
Pneumologie ... P11
Praktischer Arzt ... P12
Psychiatrie ... P13
Psychiatrie und Psychotherapie ... P14
Psychologischer Psychotherapeut ... P15
Psychosomatische Medizin und Psychotherapie ... P16
Psychotherapeutisch tätiger Arzt ... P17
Psychotherapeutische Medizin ... P18
Psychotherapie ... P19

Radiologie ... R01
Radiologische Diagnostik ... R02
Rechtsmedizin ... R03
Rheumatologie ... R04

Spezielle Geburtshilfe und Perinatalmedizin ... S01
Sozialhygiene ... S02
Sportmedizin ... S03
Sprach-, Stimm- und kindliche Hörstörungen ... S04
Strahlentherapie ... S05
Strahlentherapie und Radiologische Diagnostik ... S06

Thorax- und Kardiovascularchirurgie ... T01
Thoraxchirurgie ... T02
Transfusionsmedizin ... T03

Unfallchirurgie ... U01
Urologie ... U02

Visceralchirurgie ... V01

Copyright © Zentralinstitut für die kassenärztliche Versorgung

Herzlichen Dank für Ihre Teilnahme und wertvolle Unterstützung.

Fragebogen zur Evaluation des Projekts „Verbesserte Versorgung psychischer und neurologischer Erkrankungen“ (NPPV)

Erhebungswelle 2020

Im Auftrag der Kassenärztlichen Vereinigung Nordrhein

Kassenärztliche Vereinigung Nordrhein

ZENTRALINSTITUT FÜR DIE KASSENÄRZTLICHE VERSORGUNG IN DEUTSCHLAND

Hinweise:

Da der Fragebogen mit moderner Scanner-Technik ausgewertet wird, bitten wir Sie,

- einen schwarzen oder blauen Kugelschreiber zu verwenden,
- Ihre Angaben gut lesbar und Markierungen innerhalb der Kästchen zu machen,
- keine Anmerkungen außerhalb von Erfassungsfeldern in den Fragebogen zu schreiben,
- eventuelle Korrekturen wie folgt vorzunehmen:
  - falsch gesetzte Kreuze in Kästchen komplett schwärzen,
  - falsch eingetragene Werte komplett durchstreichen, den korrigierten Wert neben das durchgestrichene Feld neu eintragen und eindeutig zuordnen.

Aus Gründen der besseren Lesbarkeit verwenden wir in diesem Fragebogen zumeist die Sprachform des generischen Maskulinums. Wir weisen ausdrücklich darauf hin, dass die Verwendung der männlichen Form geschlechterunabhängig verstanden werden soll.

TEIL A

Angaben zum Versorgungsprojekt NPPV

Das Versorgungsprojekt NPPV wurde mit dem Ziel geschaffen, die ambulante Versorgung von Menschen mit neurologischen und psychischen Erkrankungen zu verbessern. Anhand des vorliegenden Fragebogens sollen erarbeitete Strukturen und Prozesse bewertet werden. Mit Ihrer Teilnahme an der Befragung tragen Sie dazu bei, das Versorgungsprojekt NPPV auch in Zukunft weiterentwickeln zu können.

Copyright © Zentralinstitut für die kassenärztliche Versorgung

Angaben zum Status Ihrer NPPV-Teilnahme

A1 Seit wann nehmen Sie am NPPV-Projekt teil? M M J J J J

A2 Ungefähr wie viele Ihrer Patienten sind derzeit in das NPPV-Projekt eingeschrieben? ☐☐☐

A3 Im Vergleich zu einem Patienten, der nicht im NPPV-Projekt eingeschrieben ist, fallen die Kontaktzahlen eines **NPPV-Patienten** schätzungsweise wie folgt aus:

☐ ca. ___ Kontakte **weniger** pro Quartal
☐ ca. **gleich** viele Kontakte pro Quartal
☐ ca. ___ Kontakte **mehr** pro Quartal

Strukturen und Prozesse: Allgemein

A4.1 Wenn Sie an das Projekt NPPV denken, wie schätzen Sie …

	sehr gut	gut	befriedigend	ausreichend	mangelhaft
den Austausch/die Zusammenarbeit zwischen allen Fachgruppen innerhalb des NPPV Projekts ein?	☐	☐	☐	☐	☐
den fallbezogenen Austausch zwischen Bezugsarzt/-therapeut und anderen an der Versorgung beteiligten Behandlern ein?	☐	☐	☐	☐	☐
die Einbindung therapeutischer Angebote, wie bspw. Gruppen- und Online-Selbsthilfeangebote (Novego), ein?	☐	☐	☐	☐	☐
die Unterstützung der Netzwerke durch Koordinationsstellen oder IT-Strukturen hinsichtlich der Verkürzung von Wartezeiten ein?	☐	☐	☐	☐	☐
die Unterstützung der Netzwerke durch Koordinationsstellen oder IT-Strukturen hinsichtlich der Priorisierung von Behandlungsfällen ein?	☐	☐	☐	☐	☐

Strukturen und Prozesse: IT-Systeme

A4.2 Wenn Sie an das Projekt NPPV denken, wie bewerten Sie …

	sehr gut	gut	befriedigend	ausreichend	mangelhaft
die Vertragssoftware IVPnet im Allgemeinen?	☐	☐	☐	☐	☐
die Vertragssoftware IVPnet im Hinblick auf die Verwaltung von Patientendaten?	☐	☐	☐	☐	☐
die Vertragssoftware IVPnet im Hinblick auf eine bessere Umsetzung und Verwaltung von Behandlungspfaden?	☐	☐	☐	☐	☐
die Vertragssoftware IVPnet im Hinblick auf den Informationsaustausch zwischen allen an der Versorgung Beteiligten?	☐	☐	☐	☐	☐
die Sicherheit der Vertragssoftware IVPnet (u. A. Einhaltung des Datenschutzes)?	☐	☐	☐	☐	☐

Copyright © Zentralinstitut für die kassenärztliche Versorgung

Angaben zum Versorgungsgeschehen während der Corona-Pandemie

A5.1 Wie zutreffend sind diese Aussagen aus Ihrer Sicht?
Die Strukturen und Prozesse des NPPV-Projekts helfen mir während der Corona-Pandemie…

	sehr	ziemlich	wenig	gar nicht
NPPV-Patienten weiterhin leitliniengerecht zu versorgen (u. A. mittels telemedizinischer bzw. E-Health Angebote)	☐	☐	☐	☐
den Austausch zu anderen Versorgern aufrecht zu erhalten (u. A. durch Koordinationsstellen oder Netzwerktreffen)	☐	☐	☐	☐
Umstellungen in der Regelversorgung schneller umzusetzen (z.B. die Videosprechstunde)	☐	☐	☐	☐

A5.2 Haben Sie <u>während</u> der pandemiebedingten Ausgangsbeschränkungen im Frühjahr 2020 NPPV-Leistungen mittels telemedizinischer Anwendungen angeboten?

ja	☐
nein	☐

A5.3 Haben Sie <u>vor</u> den pandemiebedingten Ausgangsbeschränkungen im Frühjahr 2020 NPPV-Leistungen mittels telemedizinischer Anwendungen angeboten?

ja	☐
nein	☐

A5.4 Welche telemedizinischen Anwendungen bieten Sie Ihren NPPV-Patienten an?

Telefonsprechstunde	☐
Videosprechstunde	☐
Andere (bitte benennen):	

A5.5 Welche Leistungen bieten Sie innerhalb der telemedizinischen Anwendungen Ihren NPPV-Patienten an?

Gruppenangebote	☐
Bezugskontakte	☐
Krisensprechstunde	☐
Psychotherapeutische Behandlungsformen	☐
E-Mental-Health (Novego)	☐
Andere (bitte benennen):	

Copyright © Zentralinstitut für die kassenärztliche Versorgung

Ergebniseinschätzung: Arbeitsbelastung

A6.1 Seit der Implementierung des Projekts NPPV hat sich...

	stark verringert	verringert	nicht verändert	erhöht	stark erhöht
die allgemeine Arbeitsbelastung	☐	☐	☐	☐	☐
die aufgewendete durchschnittliche Anzahl der Wochenstunden für die Patientenversorgung	☐	☐	☐	☐	☐
die Arbeitsbelastung durch Dokumentationen	☐	☐	☐	☐	☐
die Zufriedenheit des Praxispersonals	☐	☐	☐	☐	☐

Ergebniseinschätzung: Versorgungsqualität

A6.2 Seit der Implementierung des Projekts NPPV hat sich ...

	stark verringert	verringert	nicht verändert	erhöht	stark erhöht
die Zahl der Therapieabbrüche durch den Patienten	☐	☐	☐	☐	☐
die Zahl der nötigen Krankenhauseinweisungen	☐	☐	☐	☐	☐
die Produktivität in der Praxis	☐	☐	☐	☐	☐
der Verweis an weiterführende therapeutische Angebote	☐	☐	☐	☐	☐
die Ausrichtung auf präventive Maßnahmen	☐	☐	☐	☐	☐
	**stark verbessert**	**verbessert**	**nicht verändert**	**vermindert**	**stark vermindert**
die Qualität der Patientenversorgung	☐	☐	☐	☐	☐
die Beziehung/Bindung zu den von Ihnen betreuten Patienten im Allgemeinen	☐	☐	☐	☐	☐
die Möglichkeit, zielgenaue und flexible Therapieoptionen bereitzustellen	☐	☐	☐	☐	☐
der allgemeine Therapieerfolg bei Patienten	☐	☐	☐	☐	☐
die Möglichkeit, Krisen und Krankheitsschübe der Patienten schneller und besser zu behandeln (intensivierte ambulante Komplexbehandlung)	☐	☐	☐	☐	☐
die Zusammenarbeit mit den Arbeitsstätten der Patienten	☐	☐	☐	☐	☐
die Zusammenarbeit mit ambulanten Spezialkliniken	☐	☐	☐	☐	☐
die fachärztliche/therapeutische Versorgungsintensität	☐	☐	☐	☐	☐
die (über)regionale Vernetzung	☐	☐	☐	☐	☐
der Überblick der Patienten über ihren eigenen Gesundheitszustand	☐	☐	☐	☐	☐

Copyright © Zentralinstitut für die kassenärztliche Versorgung

Persönliche Bewertung

A7.1 Wie wichtig sind Ihnen folgende Ziele bei der Teilnahme am Projekt NPPV?

	sehr	ziemlich	wenig	gar nicht
Steigerung der Versorgungsqualität spezifischer Patientengruppen	☐	☐	☐	☐
Verringerung von Therapieabbrüchen	☐	☐	☐	☐
Effizientere Steuerung der Patientenversorgung in der Praxis	☐	☐	☐	☐
Steigerung des Praxisumsatzes	☐	☐	☐	☐
Steigerung der Patientenbindung und -zufriedenheit	☐	☐	☐	☐
Steigerung der eigenen Arbeitszufriedenheit	☐	☐	☐	☐
Möglichkeit ein innovatives Konzept mitzugestalten	☐	☐	☐	☐
Berufsgruppenübergreifende Vernetzung	☐	☐	☐	☐
Erweiterung der therapeutischen Versorgung durch Gruppen- und Online-Selbsthilfeangebote (Novego)	☐	☐	☐	☐

Sonstiges (bitte benennen):

A7.2 Wie zufrieden sind Sie mit …

	sehr zufrieden	zufrieden	teils, teils oder neutral	unzufrieden	sehr unzufrieden
Ihrer momentanen beruflichen Situation?	☐	☐	☐	☐	☐
dem Projekt NPPV im Allgemeinen?	☐	☐	☐	☐	☐
der leistungsgerechten Vergütung des erhöhten Versorgungsaufwands innerhalb des Projekts NPPV?	☐	☐	☐	☐	☐
dem Erreichen der Ziele, die Sie mit der Teilnahme beim Projekt NPPV verfolgen?	☐	☐	☐	☐	☐
den Schulungen für das Projekt NPPV?	☐	☐	☐	☐	☐
den Netzwerktreffen für das Projekt NPPV?	☐	☐	☐	☐	☐
den Qualitätszirkeln für das Projekt NPPV?	☐	☐	☐	☐	☐
der Unterstützung der Koordinationsstellen hinsichtlich der Umsetzung der allgemeinen Strukturen und Prozesse des Projekts NPPV?	☐	☐	☐	☐	☐

Sonstiges (bitte benennen):

Copyright © Zentralinstitut für die kassenärztliche Versorgung

A7.3 Wie zutreffend sind diese Aussagen aus Ihrer Sicht?

	sehr	ziemlich	wenig	gar nicht
Ich werde das Projekt auch in Zukunft weiter unterstützen	☐	☐	☐	☐
Das Projekt ist für die Erkrankten angemessen	☐	☐	☐	☐
Es fällt mir leicht, leitliniengerechte Behandlungspfade/ definierte Prozesse des Projekts einzuhalten	☐	☐	☐	☐
Durch das Projekt habe ich angefangen, therapeutische Gruppenangebote für Patienten anzubieten	☐	☐	☐	☐
Die meisten Patienten profitieren von dem Projekt	☐	☐	☐	☐
Das Projekt trägt zur Entlastung in der Patientenversorgung bei	☐	☐	☐	☐
Meine Fachkompetenzen sind ausreichend, um das Projekt leitliniengerecht auszuführen	☐	☐	☐	☐
Ich erhalte alle notwendigen Informationen, die für meine Arbeit innerhalb des Projekts essentiell sind	☐	☐	☐	☐
Die Patienten erhalten durch das Projekt mehr Werkzeuge für eine erfolgreiche Therapie	☐	☐	☐	☐
Die Öffentlichkeitsarbeit für das Projekt ist ausreichend	☐	☐	☐	☐
Ich würde das Projekt Kollegen weiterempfehlen	☐	☐	☐	☐
**… bzw. wie zutreffend bewerten Sie diese Gründe für den Austritt der Patienten aus dem NPPV-Projekt?**				
Untauglichkeit der Patienten wegen kognitiver Einschränkungen, Demenz o. Ä.	☐	☐	☐	☐
Patienten erkennen die Relevanz der Therapieempfehlung nicht	☐	☐	☐	☐
Angst der Patienten sich selbst/sich der Familie/dem sozialen Umfeld zu öffnen	☐	☐	☐	☐
Mangelnde Unterstützung der Familie/des sozialen Umfeldes/ der Arbeitsstätte der Patienten	☐	☐	☐	☐
Schwierigkeiten der Patienten, ihre Situation zu akzeptieren	☐	☐	☐	☐
Ungenügender Zugang zum Versorgernetz oder anderen Therapieeinrichtungen	☐	☐	☐	☐
Mangelndes Wissen über das Projekt hinsichtlich seiner Ziele, Strukturen und Prozesse	☐	☐	☐	☐
Überforderung der Patienten durch Evaluationen	☐	☐	☐	☐

Sonstiges (bitte benennen):

A7.4 Was sollte aus Ihrer Sicht in Zukunft am Projekt NPPV verbessert werden?

Copyright © Zentralinstitut für die kassenärztliche Versorgung

TEIL B

Angaben zur Person

Zum Abschluss bitten wir Sie um Angaben zu Ihrer Person.
Alle Daten werden anonym erfasst und nur für wissenschaftliche Forschungszwecke ausgewertet.

Copyright © Zentralinstitut für die kassenärztliche Versorgung

Angaben zum Versorger und der Versorgungstätigkeit

B1 **In welchem Jahr sind Sie geboren?** J J J J

B2 **Seit wie vielen Jahren sind Sie als niedergelassener Arzt/Psychotherapeut tätig?** ☐☐ Jahre

B3 **Welches Geschlecht haben Sie?** weiblich ☐ männlich ☐

B4 **Welche waren, bezogen auf den Umsatz, Ihre wichtigsten Zulassungsfachgebiete am Stichtag 31.12.2019?**
Die Kodierung der Zulassungsfachgebiete finden Sie auf Seite 11.

1. Zulassungsfachgebiet ☐☐☐
2. Zulassungsfachgebiet ☐☐☐
3. Zulassungsfachgebiet ☐☐☐

B5 **Welche Facharztbezeichnung gemäß Weiterbildungsordnung hatten Sie am Stichtag 31.12.2019?**
Die Kodierung der Facharztbezeichnung finden Sie auf Seite 11.

1. Facharztbezeichnung ☐☐☐
2. Facharztbezeichnung ☐☐☐
3. Facharztbezeichnung ☐☐☐

B6 **Wie viele ärztliche bzw. psychotherapeutisch tätige Vollzeit-, und Teilzeitbeschäftigte haben am Stichtag 31.12.2019 in Ihrer Praxis gearbeitet - Sie selbst mit eingerechnet?** Bitte machen Sie die Angabe in Stellen.

Vollzeit ☐☐

Teilzeit ☐☐

B7 **Welche Beschäftigungsform traf auf Sie am Stichtag 31.12.2019 zu?**

selbstständig ☐

angestellt ☐

B8 **Wie viele Patienten wurden im 4. Quartal 2019 in Ihrer Praxis versorgt?** ☐☐☐☐

B9 **Wie viele Wochenstunden haben Sie im Jahr 2019 durchschnittlich für die Patientenversorgung aufgewendet und wie verteilen sich diese auf ärztliche/psychotherapeutische Tätigkeiten?**

Wochenstunden für Patientenversorgung

☐☐ =

ohne Fortbildungen, ohne Praxismanagement

auf ärztliche/psychotherapeutische Tätigkeiten verteilt, entfallen von den Wochenstunden ...

☐☐ Std./Wo. + ☐☐ Std./Wo. + ☐☐ Std./Wo.

mit Patienten (Beratung, Untersuchung und Behandlung)

ohne Patienten (z. B. Dokumentationen und Gutachten)

Notfalleinsätze (ohne Zeiten der reinen Bereitschaft)

B10 **Arbeiten Sie derzeit in einem Arztnetz bzw. sind Sie als Psychotherapeut in einem Arztnetz eingebunden?** ja ☐ nein ☐

Copyright © Zentralinstitut für die kassenärztliche Versorgung

B11	Welche Organisationsform traf auf Ihre Praxis am Stichtag 31.12.2019 zu?	
	Einzelpraxis	☐
	Örtliche Berufsausübungsgemeinschaft	☐
	Überörtliche Berufsausübungsgemeinschaft	☐
	Medizinisches Versorgungszentrum	☐
	Andere (bitte benennen):	

B12	An welchen besonderen Versorgungsformen nimmt bzw. nahm Ihre Praxis in der Vergangenheit teil?		
		**ja**	**nein**
	Strukturierte Behandlungsprogramme (DMP) gemäß § 137f SGB V	☐	☐
	Integrierte Versorgung gemäß § 140a – d SGB V *	☐	☐
	Besondere Versorgung gemäß § 140a SGB V **	☐	☐
	Besondere ambulante ärztliche Versorgung gemäß § 73c SGB V	☐	☐
	Ambulante spezialfachärztliche Versorgung (ASV) gemäß § 116b SGB V	☐	☐

*Verträge, die vor Juli 2015 abgeschlossen wurden. **Verträge, die auf Basis der neuen Fassung des § 140a SGB V abgeschlossen wurden.

B13	Unabhängig des NPPV Projekts - Wie zufrieden sind Sie innerhalb der Regelversorgung mit ...					
		**sehr zu-frieden**	**zufrieden**	**teils, teils oder neutral**	**unzufrie-den**	**sehr un-zufrieden**
	Ihren momentanen Arbeitsbedingungen?	☐	☐	☐	☐	☐
	Ihrer Arbeit im Allgemeinen?	☐	☐	☐	☐	☐
	der Zeit, die Ihnen für die Behandlung Ihrer Patienten zur Verfügung steht?	☐	☐	☐	☐	☐
	der erfahrenen Anerkennung, die Sie für Ihre Leistungen und Mühen erhalten?	☐	☐	☐	☐	☐
	Ihrem monatlichen Einkommen aus Ihrer Arbeit?	☐	☐	☐	☐	☐

B14 **Was möchten Sie uns mitteilen? Hier finden Sie Platz für Anmerkungen und sonstige Kommentare.**

Copyright © Zentralinstitut für die kassenärztliche Versorgung

KODIERUNG

von Zulassungsfachgebiet und Facharztbezeichnung

Allgemeine Chirurgie	A01
Allgemeinmedizin	A02
Anästhesiologie	A03
Anästhesiologie und Intensivtherapie	A04
Anatomie	A05
Angiologie	A06
Arbeitshygiene	A07
Arbeitsmedizin	A08
Arzt	A09
Arzt für Kinder- und Jugendmedizin	A10
Audiologie	A11
Augenheilkunde	A12
Blutspende- und Transfusionsmedizin	B01
Chirurgie	C01
Diabetologie	D01
Diagnostische Radiologie	D02
Echokardiologie herznaher Gefäße	E01
Endokrinologie	E02
Endokrinologie und Diabetologie	E03
Experimentelle und diagnostische Mikrobiologie	E04
Fachbiologie der Medizin	F01
Fachwissenschaft Chemie und Labordiagnostik	F02
Fachwissenschaft Genetik	F03
Fachwissenschaft Immunologie	F04
Fachwissenschaft Zytologie/Histologie	F05
Forensische Psychiatrie	F06
Frauenheilkunde und Geburtshilfe	F07
Gastroenterologie	G01
Gefäßchirurgie	G02
Geriatrie	G03
Gynäkologische Endokrinologie und Reproduktionsmedizin	G04
Gynäkologische Onkologie	G05
Hals-Nasen-Ohrenheilkunde	H01
Hämatologie	H02
Hämatologie und internistische Onkologie	H03
Haut- und Geschlechtskrankheiten	H04
Herz- und Gefäßchirurgie	H05
Herzchirurgie	H06
Humangenetik	H07
Hygiene	H08
Hygiene und Umweltmedizin	H09
Immunologie	I01
Infektiologie	I02
Infektions- und Tropenmedizin	I03
Innere Medizin	I04
Innere Medizin: Angiologie	I05
Innere Medizin: Endokrinologie und Diabetologie	I06
Innere Medizin: Gastroenterologie	I07
Innere Medizin: Hämatologie und Onkologie	I08
Innere Medizin: Kardiologie	I09
Innere Medizin: Nephrologie	I10
Innere Medizin: Pneumologie	I11
Innere Medizin: Rheumatologie	I12
Innere und Allgemeinmedizin (Hausarzt)	I13
Kardiologie	K01
Kardiologie und Angiologie	K02
Kieferchirurgie	K03
Kinder- und Jugendlichen-Psychotherapeut	K04
Kinder- und Jugendpsychiatrie	K05
Kinder- und Jugendpsychiatrie und -psychotherapie	K06
Kinderchirurgie	K07
Kinderdiabetologie	K08
Kinderendokrinologie und -diabetologie	K09
Kindergastroenterologie	K10
Kinderhämatologie	K11
Kinderhämatologie und -onkologie	K12
Kinderheilkunde	K13
Kinderheilkunde und Jugendmedizin	K14
Kinderkardiologie	K15
Kinder-Lungen- und Bronchialheilkunde	K16
Kindernephrologie	K17
Kinderneuropsychiatrie	K18
Kinderpneumologie	K19
Kinderradiologie	K20
Kinderrheumatologie	K21
Kinder- und Jugendarzt	K22
Kinder- und Jugendmedizin	K23
Klinische Pharmakologie	K24
Laboratoriumsmedizin	L01
Lungen- und Bronchialheilkunde	L02
Lungenarzt	L03
Magenarzt	M01
Mikrobiologie	M02
Mikrobiologie und Infektionsepidemiologie	M03
Mikrobiologie, Virologie und Infektionsepidemiologie	M04
Mund-Kiefer-Gesichtschirurgie	M05
Neonatologie	N01
Nephrologie	N02
Nervenheilkunde	N03
Neurochirurgie	N04
Neurologie	N05
Neurologie und Psychiatrie	N06
Neurologie, Psychiatrie und Psychotherapie	N07
Neuropädiatrie	N08
Neuropathologie	N09
Neuroradiologie	N10
Nuklearmedizin	N11
Orthopädie	O01
Orthopädie und Unfallchirurgie	O02
Pathologie	P01
Pathologische Anatomie	P02
Pharmakologie und Toxikologie	P03
Phoniatrie	P04
Phoniatrie und Pädaudiologie	P05
Physikalische und Rehabilitative Medizin	P06
Physiologie	P07
Physiotherapie	P08
Plastische Chirurgie	P09
Plastische und Ästhetische Chirurgie	P10
Pneumologie	P11
Praktischer Arzt	P12
Psychiatrie	P13
Psychiatrie und Psychotherapie	P14
Psychologischer Psychotherapeut	P15
Psychosomatische Medizin und Psychotherapie	P16
Psychotherapeutisch tätiger Arzt	P17
Psychotherapeutische Medizin	P18
Psychotherapie	P19
Radiologie	R01
Radiologische Diagnostik	R02
Rechtsmedizin	R03
Rheumatologie	R04
Spezielle Geburtshilfe und Perinatalmedizin	S01
Sozialhygiene	S02
Sportmedizin	S03
Sprach-, Stimm- und kindliche Hörstörungen	S04
Strahlentherapie	S05
Strahlentherapie und Radiologische Diagnostik	S06
Thorax- und Kardiovascularchirurgie	T01
Thoraxchirurgie	T02
Transfusionsmedizin	T03
Unfallchirurgie	U01
Urologie	U02
Visceralchirurgie	V01

Copyright © Zentralinstitut für die kassenärztliche Versorgung

Herzlichen Dank für Ihre Teilnahme und wertvolle Unterstützung.

Fragebogen zur Evaluation des Projekts „Verbesserte Versorgung psychischer und neurologischer Erkrankungen“ (NPPV)

Erhebungswelle 2021

Im Auftrag der Kassenärztlichen Vereinigung Nordrhein

Kassenärztliche
Vereinigung
NORDRHEIN

ZENTRALINSTITUT FÜR DIE
KASSENÄRZTLICHE VERSORGUNG
IN DEUTSCHLAND

Hinweise:

Da der Fragebogen mit moderner Scanner-Technik ausgewertet wird, bitten wir Sie,

- einen schwarzen oder blauen Kugelschreiber zu verwenden,
- Ihre Angaben gut lesbar und Markierungen innerhalb der Kästchen zu machen,
- keine Anmerkungen außerhalb von Erfassungsfeldern in den Fragebogen zu schreiben,
- eventuelle Korrekturen wie folgt vorzunehmen:
  - falsch gesetzte Kreuze in Kästchen komplett schwärzen,
  - falsch eingetragene Werte komplett durchstreichen, den korrigierten Wert neben das durchgestrichene Feld neu eintragen und eindeutig zuordnen.

Aus Gründen der besseren Lesbarkeit verwenden wir in diesem Fragebogen zumeist die Sprachform des generischen Maskulinums. Wir weisen ausdrücklich darauf hin, dass die Verwendung der männlichen Form geschlechterunabhängig verstanden werden soll.

TEIL A

Angaben zum Versorgungsprojekt NPPV

Das Versorgungsprojekt NPPV wurde mit dem Ziel geschaffen, die ambulante Versorgung von Menschen mit neurologischen und psychischen Erkrankungen zu verbessern. Anhand des vorliegenden Fragebogens sollen erarbeitete Strukturen und Prozesse bewertet werden. Mit Ihrer Teilnahme an der Befragung tragen Sie dazu bei, das Versorgungsprojekt NPPV auch in Zukunft weiterentwickeln zu können.

Copyright © Zentralinstitut für die kassenärztliche Versorgung

Angaben zum Status Ihrer NPPV-Teilnahme

A1 Seit wann nehmen Sie am NPPV-Projekt teil? M M J J J J

A2 Ungefähr wie viele Ihrer Patienten sind derzeit in das NPPV-Projekt eingeschrieben?

A3 Im Vergleich zu einem Patienten, der nicht im NPPV-Projekt eingeschrieben ist, fallen die Kontaktzahlen eines **NPPV-Patienten** schätzungsweise wie folgt aus:

- ☐ ca. ___ Kontakte **weniger** pro Quartal
- ☐ ca. **gleich** viele Kontakte pro Quartal
- ☐ ca. ___ Kontakte **mehr** pro Quartal

Strukturen und Prozesse: Allgemein

A4.1 Wenn Sie an das Projekt NPPV denken, wie schätzen Sie …

	sehr gut	gut	befriedigend	ausreichend	mangelhaft
den Austausch/die Zusammenarbeit zwischen allen Fachgruppen innerhalb des NPPV Projekts ein?	☐	☐	☐	☐	☐
den fallbezogenen Austausch zwischen Bezugsarzt/-therapeut und anderen an der Versorgung beteiligten Behandlern ein?	☐	☐	☐	☐	☐
die Einbindung therapeutischer Angebote, wie bspw. Gruppen- und Online-Selbsthilfeangebote (Novego), ein?	☐	☐	☐	☐	☐
die Unterstützung der Netzwerke durch Koordinationsstellen oder IT-Strukturen hinsichtlich der Verkürzung von Wartezeiten ein?	☐	☐	☐	☐	☐
die Unterstützung der Netzwerke durch Koordinationsstellen oder IT-Strukturen hinsichtlich der Priorisierung von Behandlungsfällen ein?	☐	☐	☐	☐	☐

Strukturen und Prozesse: IT-Systeme

A4.2 Wenn Sie an das Projekt NPPV denken, wie bewerten Sie …

	sehr gut	gut	befriedigend	ausreichend	mangelhaft
die Vertragssoftware IVPnet im Allgemeinen?	☐	☐	☐	☐	☐
die Vertragssoftware IVPnet im Hinblick auf die Verwaltung von Patientendaten?	☐	☐	☐	☐	☐
die Vertragssoftware IVPnet im Hinblick auf eine bessere Umsetzung und Verwaltung von Behandlungspfaden?	☐	☐	☐	☐	☐
die Vertragssoftware IVPnet im Hinblick auf den Informationsaustausch zwischen allen an der Versorgung Beteiligten?	☐	☐	☐	☐	☐
die Sicherheit der Vertragssoftware IVPnet (u. A. Einhaltung des Datenschutzes)?	☐	☐	☐	☐	☐

Copyright © Zentralinstitut für die kassenärztliche Versorgung

Angaben zum Versorgungsgeschehen während der Corona-Pandemie

A5.1 Wie zutreffend sind diese Aussagen aus Ihrer Sicht?
Die Strukturen und Prozesse des NPPV-Projekts helfen mir während der Corona-Pandemie…

	sehr	ziemlich	wenig	gar nicht
NPPV-Patienten weiterhin leitliniengerecht zu versorgen (u. A. mittels telemedizinischer bzw. E-Health Angebote)	☐	☐	☐	☐
den Austausch zu anderen Versorgern aufrecht zu erhalten (u. A. durch Koordinationsstellen oder Netzwerktreffen)	☐	☐	☐	☐
Umstellungen in der Regelversorgung schneller umzusetzen (z.B. die Videosprechstunde)	☐	☐	☐	☐

A5.2 Haben Sie <u>während</u> der pandemiebedingten Kontaktbeschränkungen im Jahr 2020 oder im Jahr 2021 NPPV-Leistungen mittels telemedizinischer Anwendungen angeboten?

ja ☐ nein ☐

A5.3 Haben Sie <u>vor</u> den ersten pandemiebedingten Kontaktbeschränkungen im Frühjahr 2020 NPPV-Leistungen mittels telemedizinischer Anwendungen angeboten?

ja ☐ nein ☐

A5.4 Welche telemedizinischen Anwendungen bieten Sie Ihren NPPV-Patienten an?

Telefonsprechstunde	☐
Videosprechstunde	☐
Andere (bitte benennen):	

A5.5 Welche Leistungen bieten Sie innerhalb der telemedizinischen Anwendungen Ihren NPPV-Patienten an?

Gruppenangebote	☐
Bezugskontakte	☐
Krisensprechstunde	☐
Psychotherapeutische Behandlungsformen	☐
E-Mental-Health (Novego)	☐
Andere (bitte benennen):	

A5.6 Welche Probleme entstanden durch die Corona-Pandemie in Bezug auf Ihre ärztliche/therapeut. Tätigkeit?

(temporäre) Praxisschließung	☐
vermehrte Ausgaben für Hygieneartikel	☐
Wegfall von Patientenkontakten	☐
Andere (bitte benennen):	

Copyright © Zentralinstitut für die kassenärztliche Versorgung

Ergebniseinschätzung: Arbeitsbelastung

A6.1 Seit der Implementierung des Projekts NPPV hat sich…

	stark verringert	verringert	nicht verändert	erhöht	stark erhöht
die allgemeine Arbeitsbelastung	☐	☐	☐	☐	☐
die aufgewendete durchschnittliche Anzahl der Wochenstunden für die Patientenversorgung	☐	☐	☐	☐	☐
die Arbeitsbelastung durch Dokumentationen	☐	☐	☐	☐	☐
die Zufriedenheit des Praxispersonals	☐	☐	☐	☐	☐

Ergebniseinschätzung: Versorgungsqualität

A6.2 Seit der Implementierung des Projekts NPPV hat sich …

	stark verringert	verringert	nicht verändert	erhöht	stark erhöht
die Zahl der Therapieabbrüche durch den Patienten	☐	☐	☐	☐	☐
die Zahl der nötigen Krankenhauseinweisungen	☐	☐	☐	☐	☐
die Produktivität in der Praxis	☐	☐	☐	☐	☐
der Verweis an weiterführende therapeutische Angebote	☐	☐	☐	☐	☐
die Ausrichtung auf präventive Maßnahmen	☐	☐	☐	☐	☐
	**stark verbessert**	**verbessert**	**nicht verändert**	**vermindert**	**stark vermindert**
die Qualität der Patientenversorgung	☐	☐	☐	☐	☐
die Beziehung/Bindung zu den von Ihnen betreuten Patienten im Allgemeinen	☐	☐	☐	☐	☐
die Möglichkeit, zielgenaue und flexible Therapie-optionen bereitzustellen	☐	☐	☐	☐	☐
der allgemeine Therapieerfolg bei Patienten	☐	☐	☐	☐	☐
die Möglichkeit, Krisen und Krankheitsschübe der Patienten schneller und besser zu behandeln (intensivierte ambulante Komplexbehandlung)	☐	☐	☐	☐	☐
die Zusammenarbeit mit den Arbeitsstätten der Patienten	☐	☐	☐	☐	☐
die Zusammenarbeit mit ambulanten Spezialkliniken	☐	☐	☐	☐	☐
die fachärztliche/therapeutische Versorgungsintensität	☐	☐	☐	☐	☐
die (über)regionale Vernetzung	☐	☐	☐	☐	☐
der Überblick der Patienten über ihren eigenen Gesundheitszustand	☐	☐	☐	☐	☐

Copyright © Zentralinstitut für die kassenärztliche Versorgung

Persönliche Bewertung

A7.1 Wie wichtig sind Ihnen folgende Ziele bei der Teilnahme am Projekt NPPV?

	sehr	ziemlich	wenig	gar nicht
Steigerung der Versorgungsqualität spezifischer Patientengruppen	☐	☐	☐	☐
Verringerung von Therapieabbrüchen	☐	☐	☐	☐
Effizientere Steuerung der Patientenversorgung in der Praxis	☐	☐	☐	☐
Steigerung des Praxisumsatzes	☐	☐	☐	☐
Steigerung der Patientenbindung und -zufriedenheit	☐	☐	☐	☐
Steigerung der eigenen Arbeitszufriedenheit	☐	☐	☐	☐
Möglichkeit ein innovatives Konzept mitzugestalten	☐	☐	☐	☐
Berufsgruppenübergreifende Vernetzung	☐	☐	☐	☐
Erweiterung der therapeutischen Versorgung durch Gruppen- und Online-Selbsthilfeangebote (Novego)	☐	☐	☐	☐
Sonstiges (bitte benennen):				

A7.2 Wie zufrieden sind Sie mit …

	sehr zufrieden	zufrieden	teils, teils oder neutral	unzufrieden	sehr unzufrieden
Ihrer momentanen beruflichen Situation?	☐	☐	☐	☐	☐
dem Projekt NPPV im Allgemeinen?	☐	☐	☐	☐	☐
der leistungsgerechten Vergütung des erhöhten Versorgungsaufwands innerhalb des Projekts NPPV?	☐	☐	☐	☐	☐
dem Erreichen der Ziele, die Sie mit der Teilnahme beim Projekt NPPV verfolgen?	☐	☐	☐	☐	☐
den Schulungen für das Projekt NPPV?	☐	☐	☐	☐	☐
den Netzwerktreffen für das Projekt NPPV?	☐	☐	☐	☐	☐
den Qualitätszirkeln für das Projekt NPPV?	☐	☐	☐	☐	☐
der Unterstützung der Koordinationsstellen hinsichtlich der Umsetzung der allgemeinen Strukturen und Prozesse des Projekts NPPV?	☐	☐	☐	☐	☐
Sonstiges (bitte benennen):					

Copyright © Zentralinstitut für die kassenärztliche Versorgung

A7.3 Wie zutreffend sind diese Aussagen aus Ihrer Sicht?

	sehr	ziemlich	wenig	gar nicht
Ich werde das Projekt auch in Zukunft weiter unterstützen	☐	☐	☐	☐
Das Projekt ist für die Erkrankten angemessen	☐	☐	☐	☐
Es fällt mir leicht, leitliniengerechte Behandlungspfade/ definierte Prozesse des Projekts einzuhalten	☐	☐	☐	☐
Durch das Projekt habe ich angefangen, therapeutische Gruppenangebote für Patienten anzubieten	☐	☐	☐	☐
Die meisten Patienten profitieren von dem Projekt	☐	☐	☐	☐
Das Projekt trägt zur Entlastung in der Patientenversorgung bei	☐	☐	☐	☐
Meine Fachkompetenzen sind ausreichend, um das Projekt leitliniengerecht auszuführen	☐	☐	☐	☐
Ich erhalte alle notwendigen Informationen, die für meine Arbeit innerhalb des Projekts essentiell sind	☐	☐	☐	☐
Die Patienten erhalten durch das Projekt mehr Werkzeuge für eine erfolgreiche Therapie	☐	☐	☐	☐
Die Öffentlichkeitsarbeit für das Projekt ist ausreichend	☐	☐	☐	☐
Ich würde das Projekt Kollegen weiterempfehlen	☐	☐	☐	☐
**… bzw. wie zutreffend bewerten Sie diese Gründe für den Austritt der Patienten aus dem NPPV-Projekt?**				
Untauglichkeit der Patienten wegen kognitiver Einschränkungen, Demenz o. Ä.	☐	☐	☐	☐
Patienten erkennen die Relevanz der Therapieempfehlung nicht	☐	☐	☐	☐
Angst der Patienten sich selbst/sich der Familie/dem sozialen Umfeld zu öffnen	☐	☐	☐	☐
Mangelnde Unterstützung der Familie/des sozialen Umfeldes/ der Arbeitsstätte der Patienten	☐	☐	☐	☐
Schwierigkeiten der Patienten, ihre Situation zu akzeptieren	☐	☐	☐	☐
Ungenügender Zugang zum Versorgernetz oder anderen Therapieeinrichtungen	☐	☐	☐	☐
Mangelndes Wissen über das Projekt hinsichtlich seiner Ziele, Strukturen und Prozesse	☐	☐	☐	☐
Überforderung der Patienten durch Evaluationen	☐	☐	☐	☐
Sonstiges (bitte benennen):				

A7.4 Was sollte aus Ihrer Sicht in Zukunft am Projekt NPPV verbessert werden?

Copyright © Zentralinstitut für die kassenärztliche Versorgung

TEIL B

Angaben zur Person

Zum Abschluss bitten wir Sie um Angaben zu Ihrer Person.
Alle Daten werden anonym erfasst und nur für wissenschaftliche Forschungszwecke ausgewertet.

Copyright © Zentralinstitut für die kassenärztliche Versorgung

Angaben zum Versorger und der Versorgungstätigkeit

B1 **In welchem Jahr sind Sie geboren?** J J J J

B2 **Seit wie vielen Jahren sind Sie als niedergelassener Arzt/Psychotherapeut tätig?** ☐☐ Jahre

B3 **Welches Geschlecht haben Sie?** weiblich ☐ männlich ☐

B4 **Welche waren, bezogen auf den Umsatz, Ihre wichtigsten Zulassungsfachgebiete am Stichtag 31.12.2020?**
Die Kodierung der Zulassungsfachgebiete finden Sie auf Seite 11.

1. Zulassungsfachgebiet	2. Zulassungsfachgebiet	3. Zulassungsfachgebiet
☐☐☐	☐☐☐	☐☐☐

B5 **Welche Facharztbezeichnung gemäß Weiterbildungsordnung hatten Sie am Stichtag 31.12.2020?**
Die Kodierung der Facharztbezeichnung finden Sie auf Seite 11.

1. Facharztbezeichnung	2. Facharztbezeichnung	3. Facharztbezeichnung
☐☐☐	☐☐☐	☐☐☐

B6 **Wie viele ärztliche bzw. psychotherapeutisch tätige Vollzeit-, und Teilzeitbeschäftigte haben am Stichtag 31.12.2020 in Ihrer Praxis gearbeitet - Sie selbst mit eingerechnet?** Bitte machen Sie die Angabe in Stellen.

Vollzeit	☐☐
Teilzeit	☐☐

B7 **Welche Beschäftigungsform traf auf Sie am Stichtag 31.12.2020 zu?**

selbstständig	☐
angestellt	☐

B8 **Wie viele Patienten wurden im 4. Quartal 2020 in Ihrer Praxis versorgt?** ☐☐☐☐

B9 **Wie viele Wochenstunden haben Sie im Jahr 2020 durchschnittlich für die Patientenversorgung aufgewendet und wie verteilen sich diese auf ärztliche/psychotherapeutische Tätigkeiten?**

**Wochenstunden für Patientenversorgung**		**auf ärztliche/psychotherapeutische Tätigkeiten verteilt, entfallen von den Wochenstunden ...**				
☐☐	=	☐☐ Std./Wo.	+	☐☐ Std./Wo.	+	☐☐ Std./Wo.
**ohne Fortbildungen, ohne Praxismanagement**		**mit Patienten** (Beratung, Untersuchung und Behandlung)		**ohne Patienten** (z. B. Dokumentationen und Gutachten)		**Notfalleinsätze** (ohne Zeiten der reinen Bereitschaft)

B10 **Arbeiten Sie derzeit in einem Arztnetz bzw. sind Sie als Psychotherapeut in einem Arztnetz eingebunden?** ja ☐ nein ☐

Copyright © Zentralinstitut für die kassenärztliche Versorgung

B11 **Welche Organisationsform traf auf Ihre Praxis am Stichtag 31.12.2020 zu?**

Einzelpraxis	☐
Örtliche Berufsausübungsgemeinschaft	☐
Überörtliche Berufsausübungsgemeinschaft	☐
Medizinisches Versorgungszentrum	☐
Andere (bitte benennen):	

B12 **An welchen besonderen Versorgungsformen nimmt bzw. nahm Ihre Praxis in der Vergangenheit teil?**

	ja	nein
Strukturierte Behandlungsprogramme (DMP) gemäß § 137f SGB V	☐	☐
Integrierte Versorgung gemäß § 140a – d SGB V *	☐	☐
Besondere Versorgung gemäß § 140a SGB V **	☐	☐
Besondere ambulante ärztliche Versorgung gemäß § 73c SGB V	☐	☐
Ambulante spezialfachärztliche Versorgung (ASV) gemäß § 116b SGB V	☐	☐

*Verträge, die vor Juli 2015 abgeschlossen wurden. **Verträge, die auf Basis der neuen Fassung des § 140a SGB V abgeschlossen wurden.

B13 **Unabhängig des NPPV Projekts - Wie zufrieden sind Sie innerhalb der Regelversorgung mit ...**

	sehr zufrieden	zufrieden	teils, teils oder neutral	unzufrieden	sehr unzufrieden
Ihren momentanen Arbeitsbedingungen?	☐	☐	☐	☐	☐
Ihrer Arbeit im Allgemeinen?	☐	☐	☐	☐	☐
der Zeit, die Ihnen für die Behandlung Ihrer Patienten zur Verfügung steht?	☐	☐	☐	☐	☐
der erfahrenen Anerkennung, die Sie für Ihre Leistungen und Mühen erhalten?	☐	☐	☐	☐	☐
Ihrem monatlichen Einkommen aus Ihrer Arbeit?	☐	☐	☐	☐	☐

B14 **Was möchten Sie uns mitteilen? Hier finden Sie Platz für Anmerkungen und sonstige Kommentare.**

Copyright © Zentralinstitut für die kassenärztliche Versorgung

KODIERUNG

von Zulassungsfachgebiet und Facharztbezeichnung

Fachgebiet	Code
Allgemeine Chirurgie	A01
Allgemeinmedizin	A02
Anästhesiologie	A03
Anästhesiologie und Intensivtherapie	A04
Anatomie	A05
Angiologie	A06
Arbeitshygiene	A07
Arbeitsmedizin	A08
Arzt	A09
Arzt für Kinder- und Jugendmedizin	A10
Audiologie	A11
Augenheilkunde	A12
Blutspende- und Transfusionsmedizin	B01
Chirurgie	C01
Diabetologie	D01
Diagnostische Radiologie	D02
Echokardiologie herznaher Gefäße	E01
Endokrinologie	E02
Endokrinologie und Diabetologie	E03
Experimentelle und diagnostische Mikrobiologie	E04
Fachbiologie der Medizin	F01
Fachwissenschaft Chemie und Labordiagnostik	F02
Fachwissenschaft Genetik	F03
Fachwissenschaft Immunologie	F04
Fachwissenschaft Zytologie/Histologie	F05
Forensische Psychiatrie	F06
Frauenheilkunde und Geburtshilfe	F07
Gastroenterologie	G01
Gefäßchirurgie	G02
Geriatrie	G03
Gynäkologische Endokrinologie und Reproduktionsmedizin	G04
Gynäkologische Onkologie	G05
Hals-Nasen-Ohrenheilkunde	H01
Hämatologie	H02
Hämatologie und internistische Onkologie	H03
Haut- und Geschlechtskrankheiten	H04
Herz- und Gefäßchirurgie	H05
Herzchirurgie	H06
Humangenetik	H07
Hygiene	H08
Hygiene und Umweltmedizin	H09
Immunologie	I01
Infektiologie	I02
Infektions- und Tropenmedizin	I03
Innere Medizin	I04
Innere Medizin: Angiologie	I05
Innere Medizin: Endokrinologie und Diabetologie	I06
Innere Medizin: Gastroenterologie	I07
Innere Medizin: Hämatologie und Onkologie	I08
Innere Medizin: Kardiologie	I09
Innere Medizin: Nephrologie	I10
Innere Medizin: Pneumologie	I11
Innere Medizin: Rheumatologie	I12
Innere und Allgemeinmedizin (Hausarzt)	I13
Kardiologie	K01
Kardiologie und Angiologie	K02
Kieferchirurgie	K03
Kinder- und Jugendlichen-Psychotherapeut	K04
Kinder- und Jugendpsychiatrie	K05
Kinder- und Jugendpsychiatrie und -psychotherapie	K06
Kinderchirurgie	K07
Kinderdiabetologie	K08
Kinderendokrinologie und -diabetologie	K09
Kindergastroenterologie	K10
Kinderhämatologie	K11
Kinderhämatologie und -onkologie	K12
Kinderheilkunde	K13
Kinderheilkunde und Jugendmedizin	K14
Kinderkardiologie	K15
Kinder-Lungen- und Bronchialheilkunde	K16
Kindernephrologie	K17
Kinderneuropsychiatrie	K18
Kinderpneumologie	K19
Kinderradiologie	K20
Kinderrheumatologie	K21
Kinder- und Jugendarzt	K22
Kinder- und Jugendmedizin	K23
Klinische Pharmakologie	K24
Laboratoriumsmedizin	L01
Lungen- und Bronchialheilkunde	L02
Lungenarzt	L03
Magenarzt	M01
Mikrobiologie	M02
Mikrobiologie und Infektionsepidemiologie	M03
Mikrobiologie, Virologie und Infektionsepidemiologie	M04
Mund-Kiefer-Gesichtschirurgie	M05
Neonatologie	N01
Nephrologie	N02
Nervenheilkunde	N03
Neurochirurgie	N04
Neurologie	N05
Neurologie und Psychiatrie	N06
Neurologie, Psychiatrie und Psychotherapie	N07
Neuropädiatrie	N08
Neuropathologie	N09
Neuroradiologie	N10
Nuklearmedizin	N11
Orthopädie	O01
Orthopädie und Unfallchirurgie	O02
Pathologie	P01
Pathologische Anatomie	P02
Pharmakologie und Toxikologie	P03
Phoniatrie	P04
Phoniatrie und Pädaudiologie	P05
Physikalische und Rehabilitative Medizin	P06
Physiologie	P07
Physiotherapie	P08
Plastische Chirurgie	P09
Plastische und Ästhetische Chirurgie	P10
Pneumologie	P11
Praktischer Arzt	P12
Psychiatrie	P13
Psychiatrie und Psychotherapie	P14
Psychologischer Psychotherapeut	P15
Psychosomatische Medizin und Psychotherapie	P16
Psychotherapeutisch tätiger Arzt	P17
Psychotherapeutische Medizin	P18
Psychotherapie	P19
Radiologie	R01
Radiologische Diagnostik	R02
Rechtsmedizin	R03
Rheumatologie	R04
Spezielle Geburtshilfe und Perinatalmedizin	S01
Sozialhygiene	S02
Sportmedizin	S03
Sprach-, Stimm- und kindliche Hörstörungen	S04
Strahlentherapie	S05
Strahlentherapie und Radiologische Diagnostik	S06
Thorax- und Kardiovascularchirurgie	T01
Thoraxchirurgie	T02
Transfusionsmedizin	T03
Unfallchirurgie	U01
Urologie	U02
Visceralchirurgie	V01

Copyright © Zentralinstitut für die kassenärztliche Versorgung

Herzlichen Dank für Ihre Teilnahme und wertvolle Unterstützung.
