# Supplementary material for: A Stepped Health Services Intervention to Improve Care for Mental and Neurological Diseases: Protocol for a Prospective Cohort Trial
Source: JMIR Res Protoc. 2023 Jan 17;12:e37569. doi: 10.2196/37569 (PMC9890347; doi:10.2196/37569)
Supplement: Multimedia Appendix 3 [file resprot_v12i1e37569_app3.pdf]

Gemeinsamer
Bundesausschuss
Innovationsausschuss

Geschäftsstelle

Besuchsadresse:
Gutenbergstraße 13
10587 Berlin

Ansprechpartner/in:
Dr. Annelie Brendel

E-Mail:
Annelie.brendel@if.g-ba.de

Internet:
www.if.g-ba.de

Unser Zeichen:
01NVF16020

Datum:
22. Februar 2022

Innovationsausschuss beim G-BA, Postfach 12 06 06, 10596 Berlin

Herr Dr. med Karlheinz Großgarten
Kassenärztliche Vereinigung Nordrhein
Tersteegenstr. 9
40474 Düsseldorf

Confirmation government funding and assessment corresponding to a peer-review

Dear Dr. Großgarten,

we hereby confirm that the project „NPPV – Verbesserte Versorgung psychischer und neurologischer Erkrankungen“ is funded by the German Innovation Fund with the funding number: 01NVF16020.

Projects funded by the German Innovation Fund run through an assessment procedure that correspond to a peer-review.

Details of the assessment procedere are described in § 9 of the Code of procedure of the council of the German Innovation Fund: https://innovationsfonds.g-ba.de/downloads/media/6/Verfahrensordnung_Innovationsausschuss_2015-12-14.pdf

The application for „NPPV“ was assessed by an council of experts and finally (under consideration of the results of the experts) of the council of the German Innovation Fund (both councils existing of 10 members).

The council of the German Innovation Fund is located at the Federal Joint Committee (G-BA)[1]. The Federal Joint Committee is the highest decision-making body of the joint self-government of

[1] in German: Innovationsausschuss beim Gemeinsamen Bundesausschuss.

physicians, dentists, hospitals and health insurance funds in Germany. It issues directives for the benefit catalogue of the statutory health insurance funds (GKV) for more than 73 million insured persons and thus specifies which services in medical care reimbursed by the GKV.

The documentation of the NPPV project is accessible on the website of the funding body: NPPV – Verbesserte Versorgung psychischer und neurologischer Erkrankungen - G-BA Innovationsfonds

Please contact us if additional questions arise.

Sincerely yours,

Brendel

Dr. Annelie Brendel
